# Network Pharmacology-Assisted Multi-Omics Analysis of Gut Microbiota–Host Metabolic Remodeling and Hepatic HPD/GSTM3-Associated Redox Responses Following Shanqijing Treatment in STZ-Induced Diabetic Mice

**DOI:** 10.3390/ph19101602

**Published:** 2026-10-09

**Authors:** Xiangpeng Kong, Haiqin Ren, Jiani He, Ke Pei, Hui Zhang, Haixin Liu, Zhuqing Song, Yujia Liu, Zhuoyang Wei, Yiwei Chen, Kaifan Yang, Wenhui Deng, Yuxia Guo, Huifeng Li, Yuanbiao Qiao, Miaorong Pei

**Affiliations:** 1 Institute of Pharmaceutical & Food Engineering, Shanxi University of Chinese Medicine, Jinzhong 030619, China; kong_xiangpeng@sxtcm.edu.cn (X.K.); hejiani@sxtcm.edu.cn (J.H.); peike_pk@126.com (K.P.); liuhaixin@sxtcm.edu.cn (H.L.); 19581931302@163.com (Z.S.); 13327593366@163.com (Y.C.); 13134649391@163.com (K.Y.); guoyuxia139@163.com (Y.G.); peimr602@163.com (M.P.); 2 School of Pharmacy, Harbin University of Commerce, Harbin 150076, China; haiqinren@163.com; 3 School of Pharmacy, Faculty of Medicine, Macau University of Science and Technology, Macau SAR, China; 3240002660@student.must.edu.mo; 4 School of Life Sciences and Medical Engineering, Anhui University, Hefei 230601, China; 17835682056@163.com; 5 The Third Clinical College, Shanxi University of Chinese Medicine, Jinzhong 030619, China; 18734553156@163.com; 6 Shanxi Administration for Market Regulation, Taiyuan 030012, China; dengwenhui06404@126.com; 7 Shanxi Laboratory for the Development of New TCM Products, Shanxi University of Chinese Medicine, Jinzhong 030619, China

**Keywords:** Shanqijing, natural products, network pharmacology, multi-omics, type 1 diabetes mellitus (T1DM), gut microbiota, serum metabolomics, short-chain fatty acids (SCFAs), HPD/GSTM3, hepatic redox response

## Abstract

**Background/Objectives:** Shanqijing (SQJ) is a multibotanical aqueous extract prepared from an equal-ratio blend of Astragali Radix, Dioscoreae Rhizoma, Corni Fructus, Polygonati Rhizoma, and Polygonati Odorati Rhizoma. This study investigated the pharmacological effects and systems-level response pattern of SQJ in streptozotocin-induced diabetic mice. **Methods:** The study integrated chemical profiling, multi-omics analyses, and network pharmacology-assisted analysis with targeted assessment of hepatic HPD/GSTM3 expression and oxidative stress. **Results:** UHPLC-Orbitrap MS/MS putatively annotated 250 constituents in SQJ. SQJ lowered fasting blood glucose, improved oral glucose tolerance and serum insulin, reduced inflammatory and hepatic injury indices, and was accompanied by qualitatively improved pancreatic, hepatic, and intestinal histological appearances in representative blinded assessments. Serum metabolomics revealed the partial normalization of aromatic amino acid metabolism, lysophospholipid remodeling, α-linolenic acid metabolism, and polyunsaturated fatty acid-derived oxylipins. SQJ also reshaped the cecal microbiota and partially restored colonic short-chain fatty acids, although the SCFA response was non-monotonic across doses. Integrated metabolite–gene mapping and network pharmacology-assisted compound–target analysis prioritized metabolic and redox-associated host response nodes. Targeted hepatic assessment showed SQJ-responsive normalization of HPD and GSTM3 expression, lower MDA, and recovery of SOD activity and GSH. **Conclusions:** Collectively, SQJ treatment improved glucose-related and tissue-injury endpoints and was accompanied by coordinated intestinal microbial, circulating metabolic, and hepatic redox responses. These findings are consistent with a distributed systems-level response pattern rather than a single linear gut-to-liver mechanism and provide a basis for further evaluation of SQJ in diabetic metabolic disturbance. The causal contribution of microbial metabolites and the functional requirement for HPD/GSTM3 remain to be established.

## 1. Introduction

Type 1 diabetes mellitus (T1DM) is characterized by pancreatic β-cell injury and persistent insulin deficiency, but the resulting metabolic disturbance extends beyond hyperglycemia [1]. Chronic inflammation, intestinal microbial disturbance, hepatic oxidative stress, and altered lipid and amino acid metabolism contribute to diabetes-associated tissue injury and systemic dysfunction [2,3]. Insulin replacement is indispensable for glycemic control, yet it does not necessarily normalize these broader inflammatory and metabolic abnormalities [4,5]. Interest has therefore grown in natural product and botanical interventions that may complement glycemic management by modulating systemic metabolism and gut–host interactions [6,7].

Against this background, Shanqijing (SQJ) is prepared from an equal-ratio blend of Astragali Radix, Dioscoreae Rhizoma, Corni Fructus, Polygonati Rhizoma, and Polygonati Odorati Rhizoma. These five botanicals have established edible and traditional medicinal backgrounds in China and are included in national listings of substances traditionally used as both food and medicinal materials [8,9]. Reported constituents include diverse polysaccharides, iridoids, saponins, flavonoids, phenolic acids, and other small-molecule constituents [10,11,12,13,14]. Related edible-medicinal botanical materials have also been incorporated into health-related preparations [15]. In the present study, the blended materials underwent aqueous extraction, chitosan-assisted clarification, reduced-pressure concentration, and low-temperature drying to produce the SQJ botanical extract. Compositional characterization is important for interpreting multicomponent botanical extracts [16,17]. However, the composition of SQJ and the relationship between its metabolic effects and microbial and host responses have not been systematically characterized.

The gut microbiota is closely involved in diabetes-related metabolic inflammation [18,19]. Microbial dysbiosis can alter short-chain fatty acid (SCFA) profiles, intestinal homeostasis, immune-metabolic status, and host lipid metabolism [20,21]. In parallel, circulating metabolomic signatures in diabetes frequently involve aromatic amino acid metabolism, glycerophospholipid turnover, polyunsaturated fatty acid (PUFA)-derived oxylipins, mitochondrial fatty acid oxidation, and oxidative stress-related pathways [22,23]. For SQJ, a key unanswered question is how cecal microbial remodeling, SCFA changes, circulating metabolic responses, and hepatic stress adaptation relate within the same intervention context. Integration of circulating metabolites with disease-associated transcriptomic and biochemical network information may help prioritize host metabolic response nodes for targeted evaluation [24,25].

We therefore hypothesized that SQJ would improve glucose homeostasis in STZ-induced diabetic mice together with coordinated gut microbiota–SCFA, circulating metabolic, and hepatic redox responses. To test this hypothesis in a natural product pharmacology framework, we first chemically characterized the SQJ aqueous botanical extract by UHPLC-Orbitrap MS/MS and assessed glycemic control, insulin secretion, inflammatory status, and tissue injury. We then combined untargeted serum metabolomics, 16S rRNA gene sequencing of cecal contents, SCFA quantification in colonic contents, public T1DM-related transcriptomic analysis, metabolite–enzyme–gene integration, network pharmacology-assisted compound–target analysis, and targeted hepatic assessment of HPD/GSTM3 and oxidative stress. This study was designed to define the coordinated microbial and host metabolic responses associated with SQJ intervention and to evaluate whether they form a biologically coherent systems pharmacology response pattern.

## 2. Results

### 2.1. Chemical Characterization of the SQJ Botanical Extract

**Figure 1:**
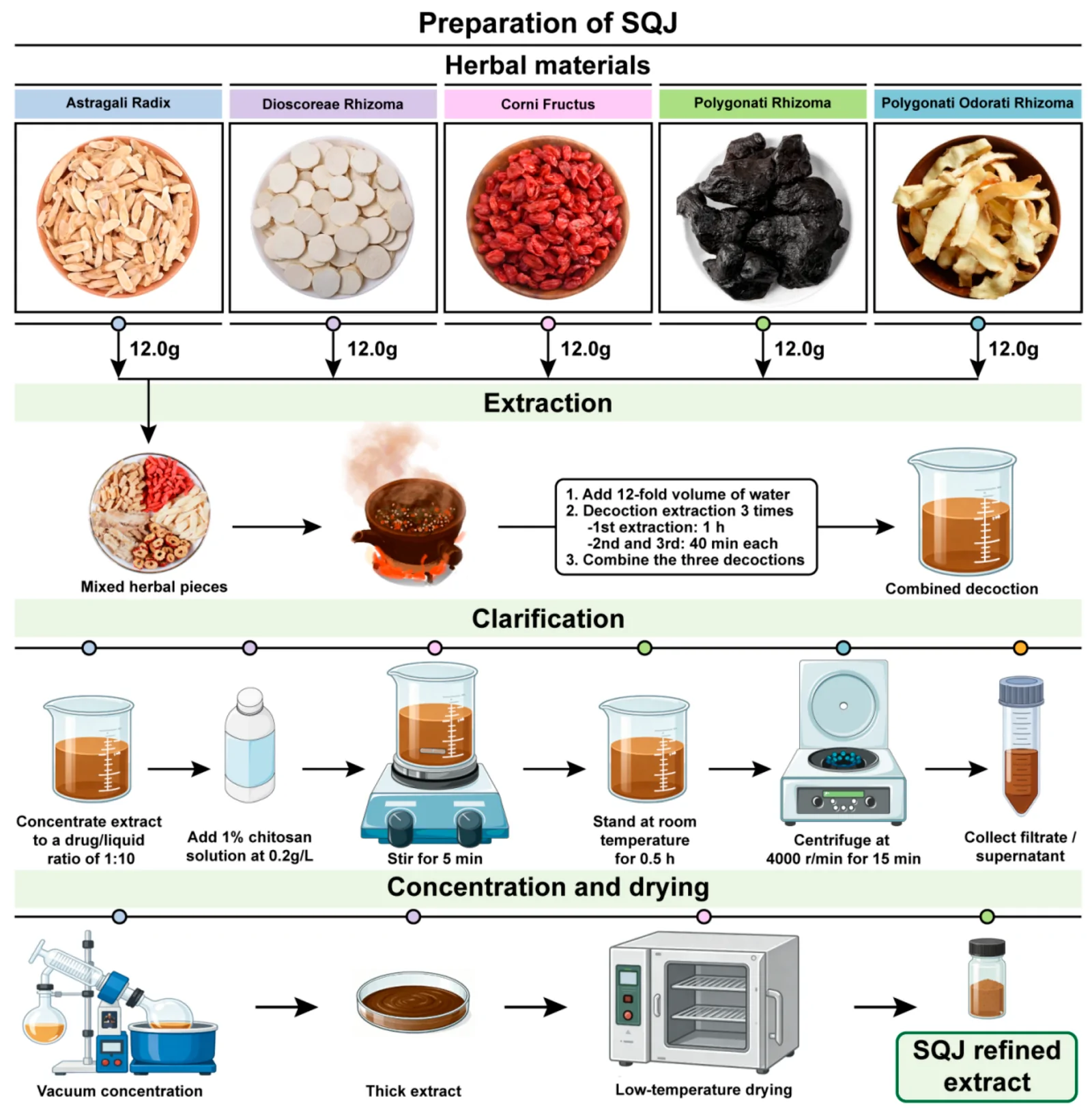
Preparation workflow of the SQJ botanical extract.

The preparation workflow of the SQJ botanical extract is shown in Figure 1.

The UHPLC-Orbitrap MS/MS analysis was performed in positive and negative electrospray ionization modes to characterize the chemical matrix (Figure S1). The total ion chromatograms contained abundant peaks across the acquisition window, indicating substantial chemical complexity. Based on accurate mass, retention behavior, fragment ions, adduct ions, and comparison with the in-house SQJ database and literature-reported MS/MS information [26,27,28,29,30,31,32], 250 constituents derived from the five botanical materials were putatively annotated (Table S1). Of the 250 retained constituents, 249 were assigned putative MSI Level 2 annotations based on integrated evidence from accurate mass, precursor/adduct assignment, diagnostic MS/MS fragmentation, retention behavior, botanical-source information, and literature-reported mass spectrometric data. One constituent (No. 38, 4-guanidinobutanoate) lacked diagnostic MS/MS fragment information and was therefore conservatively retained as an MSI Level 3 tentative assignment (Table S1). The putatively annotated constituents represented a chemically diverse set of structural classes, including saccharide- and oligosaccharide-related compounds, amino acids and nucleosides, organic and phenolic acids, flavonoids and isoflavonoids, iridoid glycosides, triterpenoid and steroidal saponins, and other small-molecule constituents (Table S1). Representative examples included morroniside and loganin from Corni Fructus, calycosin and calycosin-7-O-glucoside from Astragali Radix, multiple astragalosides and soyasaponins from Astragali Radix, and diosgenin/dioscin-related constituents associated with the Dioscorea and Polygonatum components. This broad chemical distribution is consistent with the multibotanical origin of SQJ and provides a compositional context for interpreting its distributed biological responses.

**Figure 2:**
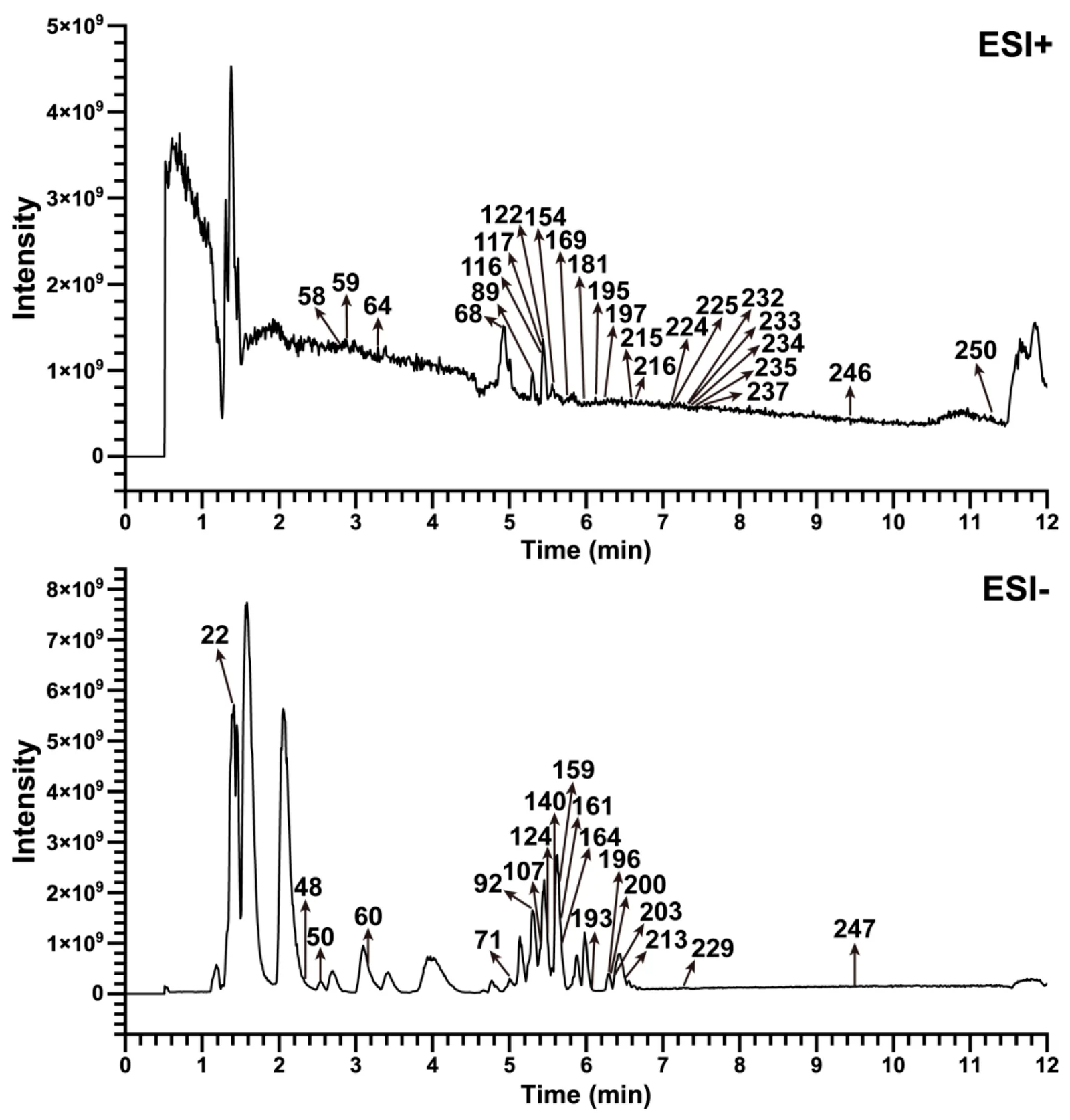
Chemical profile and representative marker annotation of the SQJ botanical extract by UHPLC-Orbitrap MS/MS. Representative total ion chromatograms (TICs) of the SQJ botanical extract acquired in positive (ESI+) and negative (ESI−) electrospray ionization modes are shown. A total of 250 chemical constituents derived from the five botanical materials of SQJ were putatively annotated by matching accurate mass, retention behavior, fragment ions, adduct ions, and literature-reported MS/MS information with the in-house SQJ constituent database, as detailed in Table S1. The numbered peaks in the TICs indicate representative literature-supported marker constituents selected as recognizable chemical reference features for the five botanical components; they were not interpreted as evidence of direct bioactivity.

To provide a composition-linked reference for the biological study, representative constituents previously reported for the five botanical components were highlighted in the total ion chromatograms (Figure 2). These numbered features were selected from the putatively annotated constituents in Table S1 on the basis of literature-supported botanical-source attribution and marker relevance. They were used as recognizable chemical reference features for the multicomponent SQJ matrix and as the chemical input set for the subsequent hypothesis-generating network pharmacology-assisted analysis, rather than as evidence that individual compounds were absorbed, directly bioactive, or responsible for the observed metabolic effects. To further strengthen the quantitative standardization of the administered SQJ extract, four representative quality-control marker constituents were quantified using authentic reference standards. Across three SQJ batches, the contents of morroniside, loganin, calycosin-7-O-glucoside, and astragaloside IV were 2.169 ± 0.105, 1.569 ± 0.084, 0.218 ± 0.030, and 0.1816 ± 0.0023 mg/g dried extract, respectively (mean ± SD, n = 3 batches). Morroniside, loganin, and calycosin-7-O-glucoside were quantified by UPLC-UV, whereas astragaloside IV was determined by HPLC-ELSD. Representative chromatograms and detailed calibration and batch-level quantitative results are provided in Figure S2 and Table S2.

### 2.2. SQJ Improves Glycemic Homeostasis, Inflammatory Status, and Tissue Integrity in Diabetic Mice

**Figure 3:**
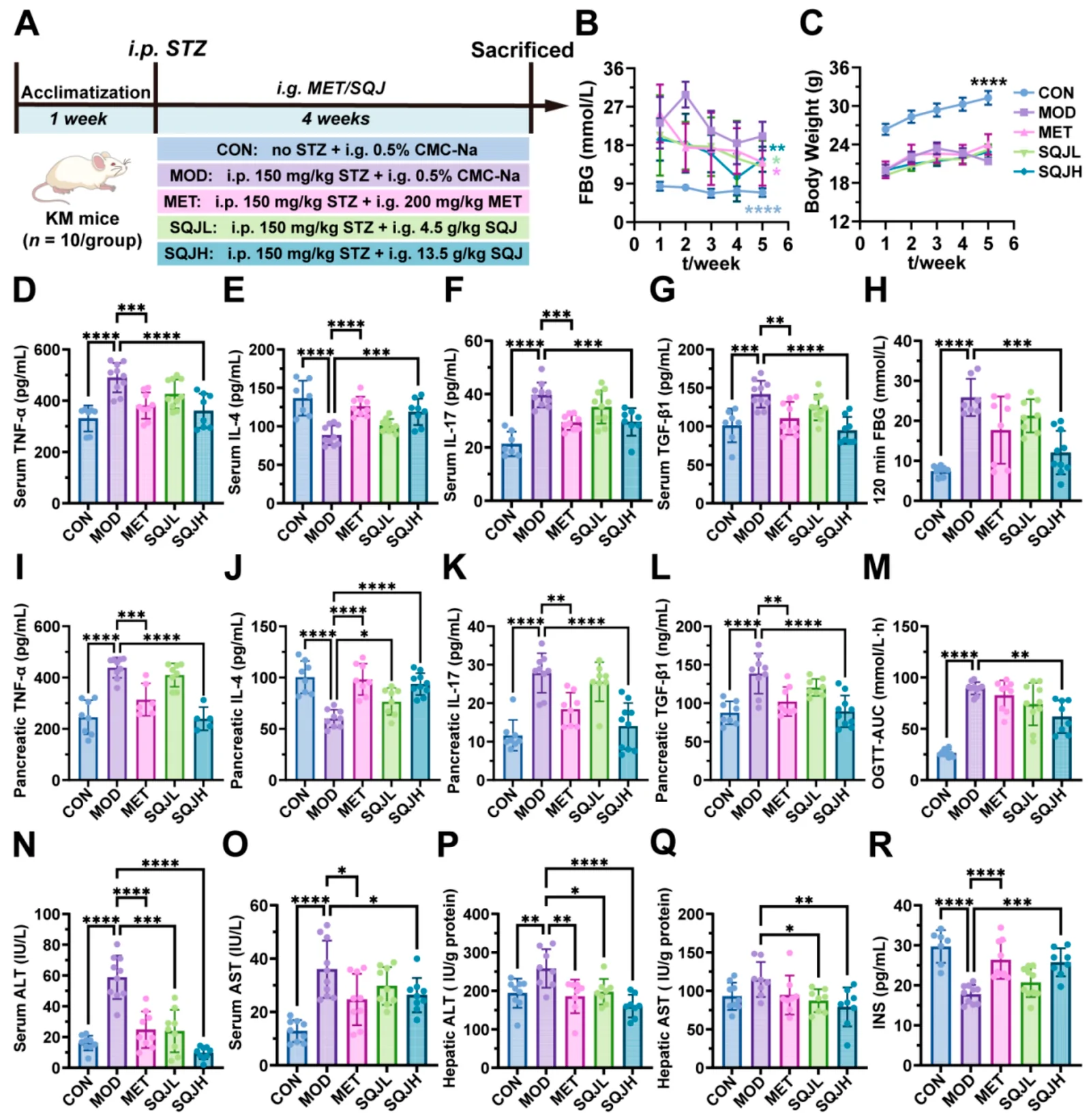
Animal experiment flowchart and efficacy assessment of SQJ botanical extract. (**A**) Schematic of the experimental design and treatment schedule. (**B**) Fasting blood glucose (FBG) levels. (**C**) Body weight changes. (**D**–**G**) Serum levels of inflammatory cytokines, including TNF-α (**D**), IL-4 (**E**), IL-17 (**F**), and TGF-β1 (**G**). (**H**) Blood glucose level at 120 min after the oral glucose tolerance test (OGTT). (**I**–**L**) Pancreatic levels of inflammatory cytokines, including TNF-α (**I**), IL-4 (**J**), IL-17 (**K**), and TGF-β1 (**L**). Pancreatic cytokine levels were measured in standardized pancreatic homogenate supernatants prepared using the same tissue-to-buffer ratio and expressed as pg/mL. (**M**) OGTT-derived area under the curve (OGTT-AUC). (**N**,**O**) Serum levels of liver function markers, including ALT (**N**) and AST (**O**). (**P**,**Q**) Hepatic levels of ALT (**P**) and AST (**Q**). (**R**) Serum insulin (INS) levels. Longitudinal fasting blood glucose and body-weight data in panels B and C were analyzed using two-way repeated-measures ANOVA followed by Dunnett’s multiple-comparison test. Other single-endpoint data were analyzed using one-way ANOVA followed by Dunnett’s post hoc test, unless otherwise indicated. * *p* < 0.05, ** *p* < 0.01, *** *p* < 0.001, **** *p* < 0.0001 vs. MOD.

The experimental design is summarized in Figure 3A. After one week of acclimatization, STZ-induced diabetic mice were established and then orally treated with metformin or SQJ botanical extract for four weeks. Longitudinal analysis of fasting blood glucose and body weight showed significant treatment- and time-dependent changes during the 28-day intervention period (Figure 3B,C). Compared with the CON group, the MOD group exhibited persistent hyperglycemia and reduced body weight during the treatment period, with a significant difference in body weight observed at the endpoint (*p* < 0.0001; Figure 3B,C). Compared with the MOD group, SQJ botanical extract reduced FBG levels at the endpoint, with significant effects observed in the SQJL and SQJH groups (*p* < 0.05 and *p* < 0.01, respectively; Figure 3B). The 120 min post-load glucose level, OGTT-derived AUC, hepatic injury markers, and insulin secretion were also improved after SQJ botanical extract treatment. Compared with the CON group, the MOD group showed an increased blood glucose level at 120 min after oral glucose loading and an elevated OGTT-derived AUC (*p* < 0.0001; Figure 3H,M). Compared with the MOD group, SQJH significantly reduced the 120 min post-glucose blood glucose level and OGTT-derived AUC (Figure 3H,M). In addition, serum ALT and AST levels and hepatic ALT levels were increased after STZ exposure, while hepatic AST showed a similar upward trend (Figure 3N–Q). SQJ botanical extract, particularly SQJH, reduced serum ALT and AST levels as well as hepatic ALT and AST levels compared with the MOD group (Figure 3N–Q). Serum insulin levels were markedly decreased in the MOD group compared with the CON group (*p* < 0.0001), and SQJH significantly restored insulin levels compared with the MOD group (*p* < 0.001; Figure 3R). These results indicate that SQJ botanical extract improved glycemic control, alleviated liver injury, and partially restored insulin secretion in STZ-induced diabetic mice.

STZ-induced diabetes was accompanied by marked inflammatory dysregulation in serum and pancreatic tissues. Compared with the CON group, the MOD group showed increased serum levels of TNF-α, IL-17, and TGF-β1, together with a decreased serum IL-4 level (*p* < 0.001 to *p* < 0.0001; Figure 3D–G). Compared with the MOD group, SQJ botanical extract, particularly SQJH, reduced serum TNF-α, IL-17, and TGF-β1 levels and partially restored serum IL-4 levels (Figure 3D–G). Similar regulatory effects were observed in pancreatic tissues, where SQJH decreased pancreatic TNF-α, IL-17, and TGF-β1 levels and increased IL-4 levels relative to the MOD group (Figure 3I–L).

**Figure 4:**
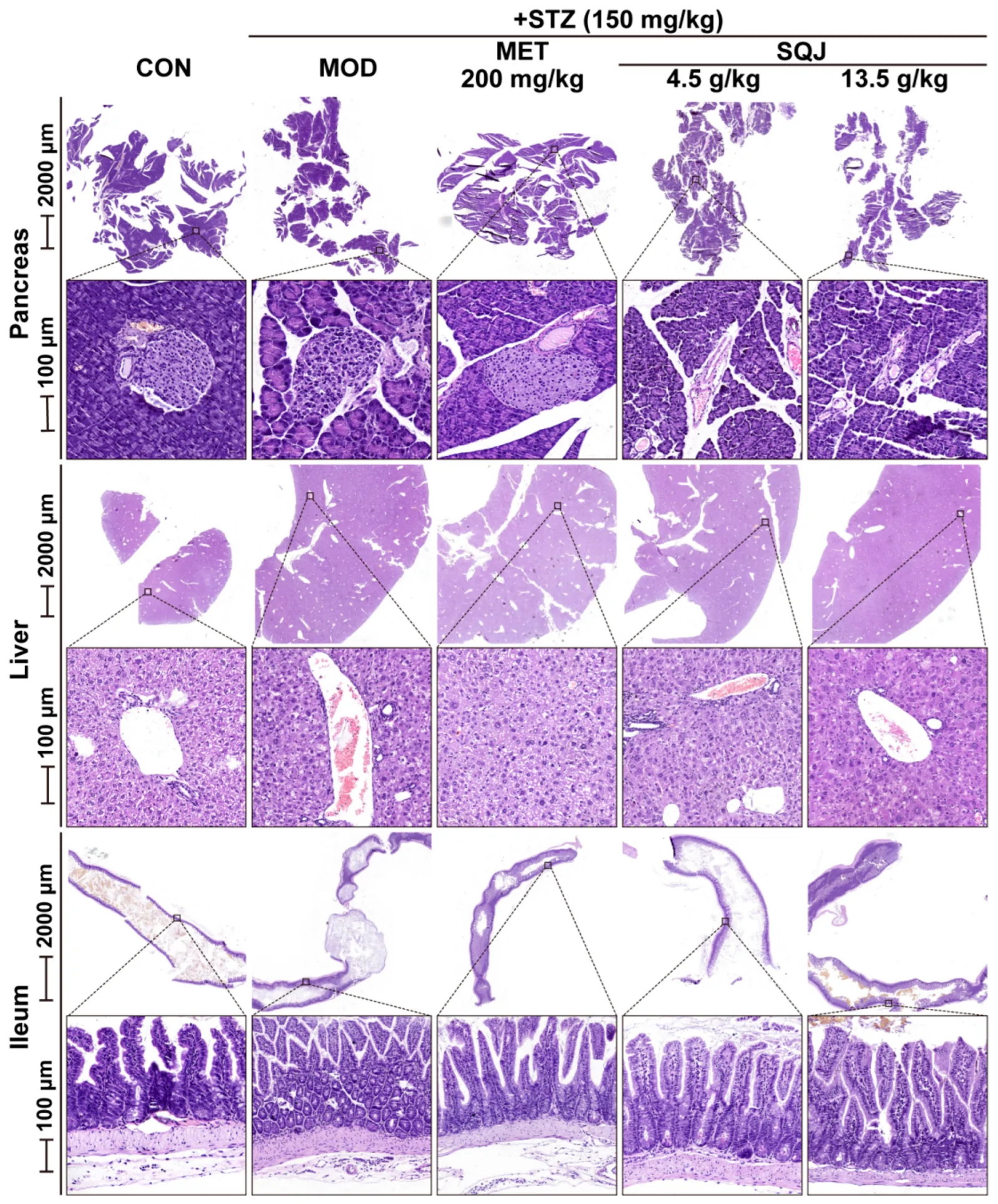
Representative histological appearance after SQJ botanical extract intervention in diabetic mice. Representative hematoxylin and eosin (*H&E*)-stained sections of the pancreas, liver, and ileum from the CON, MOD, MET, SQJL, and SQJH groups are shown. For each tissue, low-magnification overview images and corresponding enlarged regions are presented. Sections were obtained from three randomly selected animals per group, coded, and assessed qualitatively by an evaluator blinded to group allocation. No formal semiquantitative histological score was generated. Scale bars: 2000 μm for overview images and 100 μm for magnified images, as indicated.

*H&E* staining was used for blinded qualitative assessment of pathological changes in the pancreas, liver, ileum, spleen, kidney, and duodenum. Coded sections were evaluated without knowledge of group allocation. As shown in Figure 4, representative sections from control mice displayed relatively intact pancreatic islet morphology, organized hepatic architecture, and regular ileal villi with preserved mucosal structure. In contrast, representative sections from STZ-induced diabetic mice showed pancreatic, hepatic, and intestinal abnormalities, including disrupted pancreatic islet morphology, disordered exocrine pancreatic arrangement, hepatic structural disturbance around the vascular region, and ileal villus deformation with epithelial disorganization. Sections from metformin- and SQJ-treated animals showed qualitatively better-preserved tissue architecture than those from the MOD group, with the SQJH group showing the clearest qualitative improvement among the SQJ groups. Representative spleen, kidney, and duodenum sections showed a similar qualitative pattern (Figure S3). Because only three animals per group were examined and no prespecified semiquantitative scoring system was applied, these images are interpreted as supportive morphological observations rather than quantitative evidence of treatment efficacy. The physiological and inflammatory findings prompted further examination of the systemic metabolic response by serum metabolomics.

### 2.3. SQJ Remodels Diabetes-Associated Circulating Metabolic Disturbances

**Figure 5:**
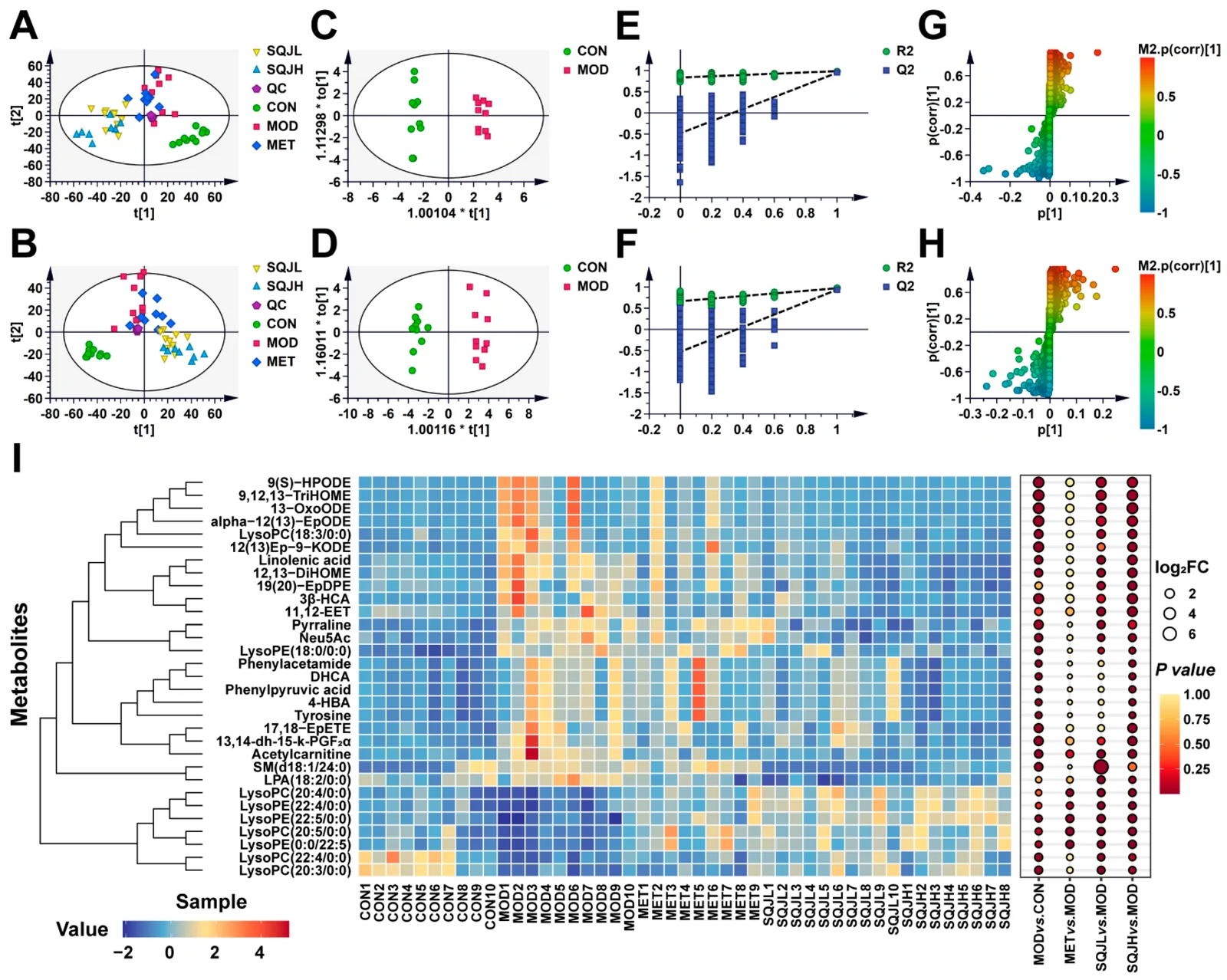
Multivariate analysis and hierarchical clustering of serum metabolomic profiles. (**A**,**B**) Principal component analysis (PCA) score plots of serum metabolomic profiles from CON, MOD, MET, SQJL, SQJH, and quality control (QC) samples in positive- and negative-ion modes, respectively. (**C**,**D**) Orthogonal partial least-squares discriminant analysis (OPLS-DA) score plots comparing the CON and MOD groups. The positive-ion model yielded *R^*2*^X* = 0.487, *R^*2*^Y* = 0.988, and *Q^*2*^* = 0.948, whereas the negative-ion model yielded *R^*2*^X* = 0.422, *R^*2*^Y* = 0.973, and *Q^*2*^* = 0.936. (**E**,**F**) Results of 200-permutation tests. The *R^*2*^* and *Q^*2*^* intercepts were 0.836 and −0.480 in positive-ion mode and 0.666 and −0.529 in negative-ion mode, respectively. (**G**,**H**) S-plots showing variables contributing to group separation, with points colored according to correlation loading values. (**I**) Hierarchical clustering heatmap of the 24 putatively annotated differential serum metabolites that met *VIP* > 1 and Benjamini–Hochberg-adjusted *p* < 0.05 in the CON-versus-MOD comparison. The right panel summarizes pairwise comparisons by log_2_ fold change (log_2_FC) and *p* value. Each biological sample represents serum from one independent animal (*n* = 8–10 per group); pooled QC samples were used to only monitor analytical stability and were not treated as biological replicates.

Untargeted serum metabolomic profiling was performed to investigate metabolic alterations associated with STZ-induced diabetes and SQJ botanical extract intervention. The total ion chromatograms of serum QC samples acquired in positive- and negative-ion modes are shown in Figure S4. PCA score plots showed distinct group-level metabolic distributions among the CON, MOD, MET, SQJL, and SQJH groups in both ionization modes, while QC samples clustered closely, indicating acceptable analytical stability and reproducibility (Figure 5A,B). OPLS-DA further showed separation between the CON and MOD groups (Figure 5C,D). In positive-ion mode, the model yielded *R^*2*^X* = 0.487, *R^*2*^Y* = 0.988, and *Q^*2*^* = 0.948; the *R^*2*^* and *Q^*2*^* intercepts from the 200-permutation test were 0.836 and −0.480, respectively. In negative-ion mode, the corresponding values were *R^*2*^X* = 0.422, *R^*2*^Y* = 0.973, and *Q^*2*^* = 0.936, with permutation-test *R^*2*^* and *Q^*2*^* intercepts of 0.666 and −0.529, respectively (Figure 5E,F). These model parameters and permutation-test results are reported to permit direct assessment of model fit and predictive performance. S-plot analysis was used to visualize variables contributing to group discrimination (Figure 5G,H). After Benjamini–Hochberg correction across all eligible metabolic features, 24 putatively annotated metabolites met the combined screening criteria of *VIP* > 1 and adjusted *p* < 0.05 for the CON-versus-MOD comparison. Hierarchical clustering of these metabolites indicated diabetes-associated remodeling involving aromatic amino acid-related metabolites, lysophospholipids, fatty acids, and PUFA-derived oxylipins (Figure 5I). The MET and SQJ groups showed partial shifts toward the CON-associated pattern, with a more pronounced tendency in the SQJH group for several metabolite clusters. Detailed information and changing trends for the 24 putatively annotated differential metabolites are provided in Table S3.

**Figure 6:**
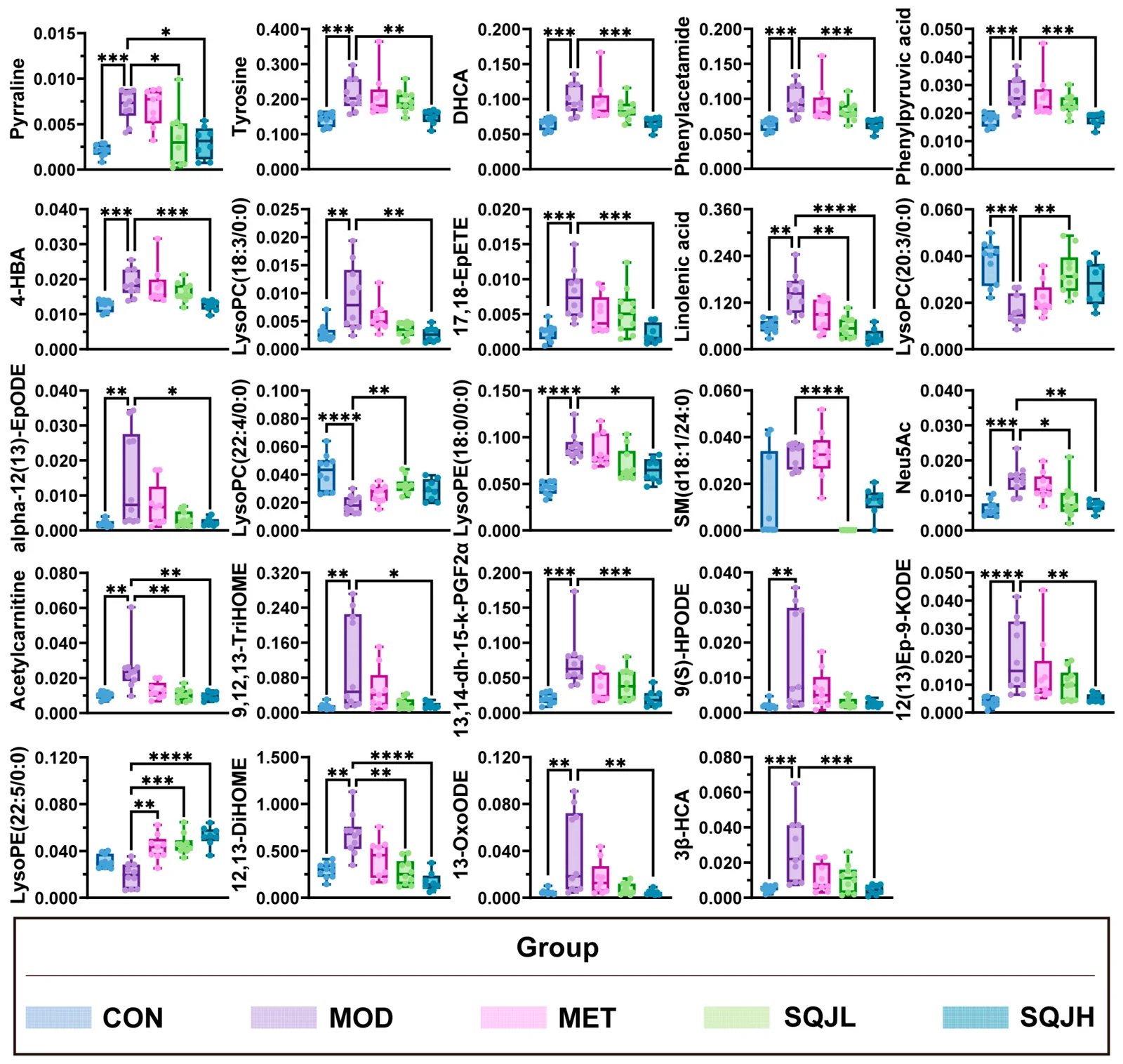
Relative abundance of representative putatively annotated serum differential metabolites after SQJ botanical extract intervention. Box plots show representative members of the 24 serum metabolites that met both *VIP* > 1 and Benjamini–Hochberg-adjusted *p* < 0.05 in the CON-versus-MOD comparison. These features include amino acid-related metabolites, lysophospholipids, oxylipins, bile acid-related metabolites, and other diabetes-associated metabolic features. Data are presented as medians with 25th–75th percentiles and minimum–maximum (*n* = 8–10 per group). The arrows and significance symbols for comparisons among the experimental groups were derived separately using the Kruskal–Wallis test followed by Dunn’s post hoc test. * *p* < 0.05, ** *p* < 0.01, *** *p* < 0.001, and **** *p* < 0.0001 vs. MOD.

Representative members of the 24 putatively annotated differential metabolites were further visualized using box plots (Figure 6). Relative to the CON group, the MOD group showed FDR-significant alterations in metabolites related to aromatic amino acid metabolism, lysophospholipid remodeling, fatty acid metabolism, and oxylipin pathways (Benjamini–Hochberg-adjusted *p* < 0.05; Table S3). Across the treatment groups, Kruskal–Wallis analysis followed by Dunn’s post hoc test indicated changes in several of these metabolites relative to the MOD group, particularly in the SQJH group (Figure 6). These findings indicate partial normalization of diabetes-associated changes in aromatic amino acid-derived metabolites, lysophospholipids, and PUFA-derived lipid mediators after SQJ intervention.

**Figure 7:**
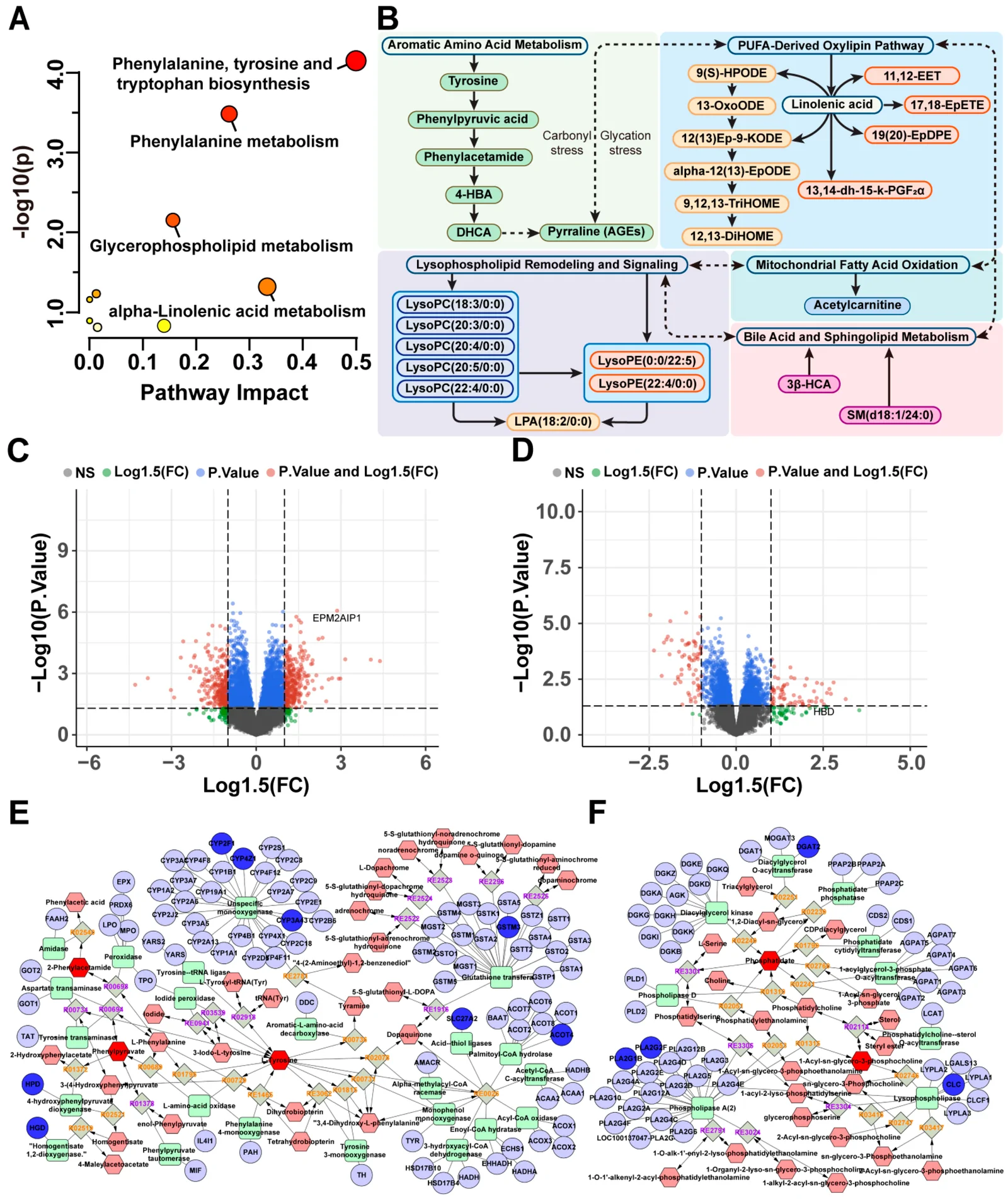
Metabolic pathway enrichment, metabolite interaction network, and network pharmacology-assisted multi-omics analysis with GEO-derived T1DM-related genes. (**A**) Pathway enrichment analysis of SQJ-regulated serum differential metabolites, showing pathway impact and −log10 (*p*-value). (**B**) Metabolic interaction network linking differential metabolites to amino acid metabolism, PUFA-derived oxylipin pathways, lysophospholipid remodeling and signaling, mitochondrial fatty acid oxidation, bile acid metabolism, and sphingolipid metabolism. (**C**,**D**) Volcano plots visualizing gene-level expression differences in the human peripheral-blood datasets GSE55098 and GSE156035. FDR-supported genes were defined by |log_2_FC| > 0.585 and BH-adjusted *p* < 0.05; results meeting only raw *p* < 0.05 and the same fold-change threshold were considered exploratory. (**E**,**F**) Compound–reaction–enzyme–gene (CREG) networks integrating serum metabolomics with GEO-derived diabetes-related targets. Node types include red hexagons for compounds, gray diamonds for reactions, green squares for enzymes, and blue circles for genes. Darker colors highlight key nodes, including deep red for significantly altered metabolites and deep blue for prioritized candidate host response nodes.

To further interpret the biological relevance of these metabolic changes, pathway enrichment and metabolite network analyses were performed. Pathway enrichment analysis identified phenylalanine, tyrosine and tryptophan biosynthesis, phenylalanine metabolism, glycerophospholipid metabolism, and α-linolenic acid metabolism as the most prominently altered pathways (Figure 7A). Network mapping of the differential metabolites further placed these changes within aromatic amino acid metabolism, PUFA-derived oxylipin pathways, lysophospholipid remodeling and signaling, mitochondrial fatty acid oxidation, and bile acid/sphingolipid-related metabolism (Figure 7B). Collectively, these findings suggest that SQJ botanical extract reshaped diabetes-associated serum metabolic disturbances, particularly those related to aromatic amino acid metabolism, lipid remodeling, and PUFA-derived inflammatory lipid mediator pathways. These circulating metabolic changes were next integrated with public T1DM transcriptomic datasets to prioritize associated host response nodes.

### 2.4. Network Pharmacology-Assisted Multi-Omics Analyses Prioritize Metabolic and Redox-Associated Host Nodes

Gene-level differential-expression analysis was performed independently in GSE55098 and GSE156035, with Benjamini–Hochberg (BH) correction applied across all genes tested within each dataset (Figure 7C,D). Using |log_2_FC| > 0.585 and BH-adjusted *p* < 0.05, no genes met the FDR criterion in GSE55098 (0/20,845), whereas 311 genes met this criterion in GSE156035 (311/43,315). At the prespecified exploratory threshold of raw *p* < 0.05 and |log_2_FC| > 0.585, 153 and 1,540 genes were identified, respectively. Among the 19,599 genes measured in both datasets, 12 met the exploratory threshold in both, of which 11 changed in the same direction; no gene met the FDR criterion in both datasets. Accordingly, the two gene lists were not pooled and treated as replicated DEGs. Instead, FDR-supported results from each dataset and cross-dataset directional consistency were retained as separate evidence layers (Table S7). These peripheral-blood transcriptomic data were then used to contextualize, rather than define, the metabolite-centered network. Metscape-based compound–reaction–enzyme–gene mapping linked tyrosine, phenylpyruvic acid, phenylacetamide, 4-HBA, DHCA, pyrraline, and lipid-related metabolites with HPD, HGD, GST-family members, GSTM3, and genes involved in phospholipid conversion and lipid remodeling (Figure 7E,F). Separately, the original exploratory network pharmacology analysis, retained in Supplementary Figure S5 for transparency, comprised 33 genes overlapping between the SQJ-associated putative target set and the nominal-threshold union of the two GEO candidate lists. This supplementary network was interpreted only as a hypothesis-generating topological context and not as FDR-supported or cross-dataset replicated transcriptomic evidence.

### 2.5. SQJ Reshapes the Cecal Microbiota and Partially Restores Colonic SCFA Profiles

**Figure 8:**
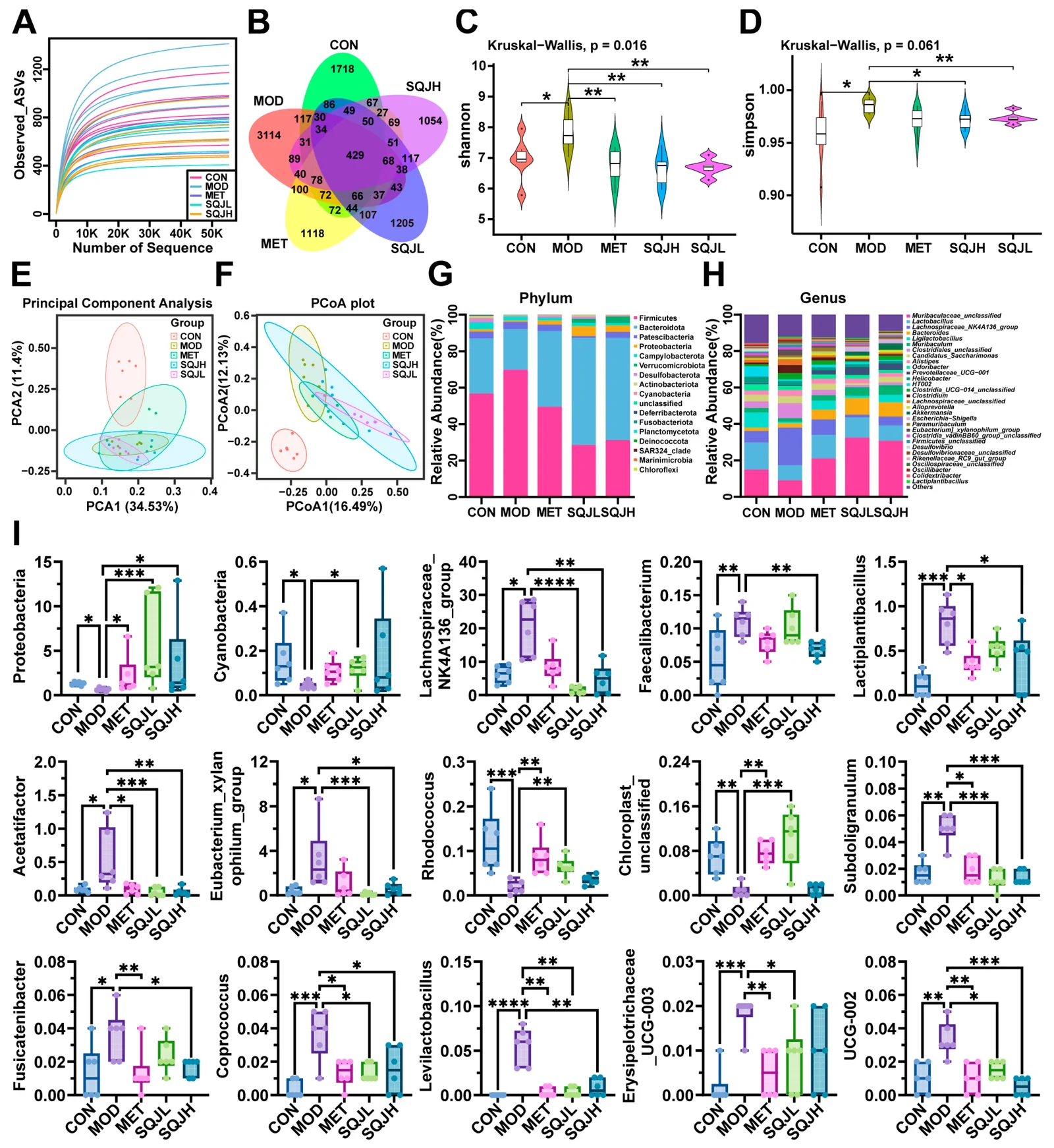
Regulatory effects of SQJ botanical extract on gut microbiota structure and composition in diabetic mice based on 16S rRNA gene sequencing. (**A**) Rarefaction curves based on observed amplicon sequence variants (ASVs). (**B**) Venn diagram showing shared and unique ASVs among groups. (**C**,**D**) Alpha diversity assessed using the Shannon (**C**) and Simpson (**D**) indices. (**E**,**F**) Beta-diversity ordination assessed using principal component analysis (PCA; **E**) and principal coordinate analysis based on the Bray–Curtis distance matrix (PCoA; (**F**)). Each point represents one biological sample (*n* = 6). Differences in overall microbial community composition were formally evaluated using PERMANOVA with 9,999 permutations. A significant overall group effect was observed (*R^*2*^* = 0.314, pseudo-F = 2.863, *p* < 0.001). Pairwise PERMANOVA also identified a significant difference between the CON and MOD groups (*R^*2*^* = 0.290, pseudo-F = 4.089, Benjamini–Hochberg-adjusted *p* = 0.0024). (**G**,**H**) Relative abundance of gut microbiota at the phylum (**G**) and genus (**H**) levels. (**I**) Relative abundance of selected bacterial taxa differing among groups. Data are presented as medians with 25–75th percentiles and minimum–maximum (*n* = 6). Statistical significance was evaluated using the Kruskal–Wallis test followed by Dunn’s post hoc test. * *p <* 0.05, ** *p <* 0.01, *** *p <* 0.001, **** *p <* 0.0001 vs. MOD.

16S rRNA gene sequencing was performed to evaluate the regulatory effects of SQJ botanical extract on gut microbiota composition. Rarefaction curves gradually approached a plateau, indicating that the sequencing depth was sufficient to capture the majority of microbial diversity within the analyzed samples (Figure 8A). The Venn diagram showed both shared and group-specific ASVs among the CON, MOD, MET, SQJL, and SQJH groups (Figure 8B). Alpha-diversity analysis revealed a significant overall difference in the Shannon index among groups (Kruskal–Wallis *p* = 0.016), with significant pairwise differences between the MOD group and the CON or treatment groups (Figure 8C). The Simpson index showed a similar trend, although the overall difference did not reach statistical significance (Kruskal–Wallis *p* = 0.061; Figure 8D).

Beta-diversity ordination indicated differences in microbial community composition among the five groups (Figure 8E,F, Table S6). This observation was formally supported by PERMANOVA based on the Bray–Curtis distance matrix, which identified a significant overall group effect (*R*^2^ = 0.314), pseudo-(F = 2.863), (*p* < 0.001); 9,999 permutations). Pairwise PERMANOVA demonstrated a significant difference between the CON and MOD groups (*R^*2*^* = 0.290), pseudo-(F = 4.089), Benjamini–Hochberg-adjusted (*p* = 0.0024)). Significant differences from the MOD group were also observed for the MET (*R^*2*^*=0.177), pseudo-(F = 2.150), adjusted (*p* = 0.0024)), SQJL (*R^*2*^* = 0.241), pseudo-(F = 3.179), adjusted (*p* = 0.0024)), and SQJH (*R^*2*^* = 0.205), pseudo-(F = 2.572), adjusted (*p* = 0.0024)) groups. Consistent overall differences were obtained using the Jaccard, unweighted UniFrac, and weighted UniFrac distance matrices (all (*p* < 0.001)). Collectively, these findings indicate that STZ-induced diabetes was associated with an altered cecal microbial community structure and that SQJ treatment was associated with a partial shift away from the MOD-associated microbial configuration.

At the phylum level, Firmicutes and Bacteroidota were the dominant bacterial phyla, and their relative abundances differed among groups (Figure 8G). At the genus level, STZ-induced diabetes altered multiple bacterial taxa, including Lachnospiraceae_NK4A136_group, Lactiplantibacillus, Acetatifactor, Eubacterium_xylanophilum_group, Rhodococcus, Subdoligranulum, Coprococcus, and UCG-002. SQJ botanical extract significantly modified several of these taxa compared with the MOD group, indicating that SQJ produced exposure-dependent and taxon-specific shifts in the diabetes-associated cecal microbial community (Figure 8H,I).

**Figure 9:**
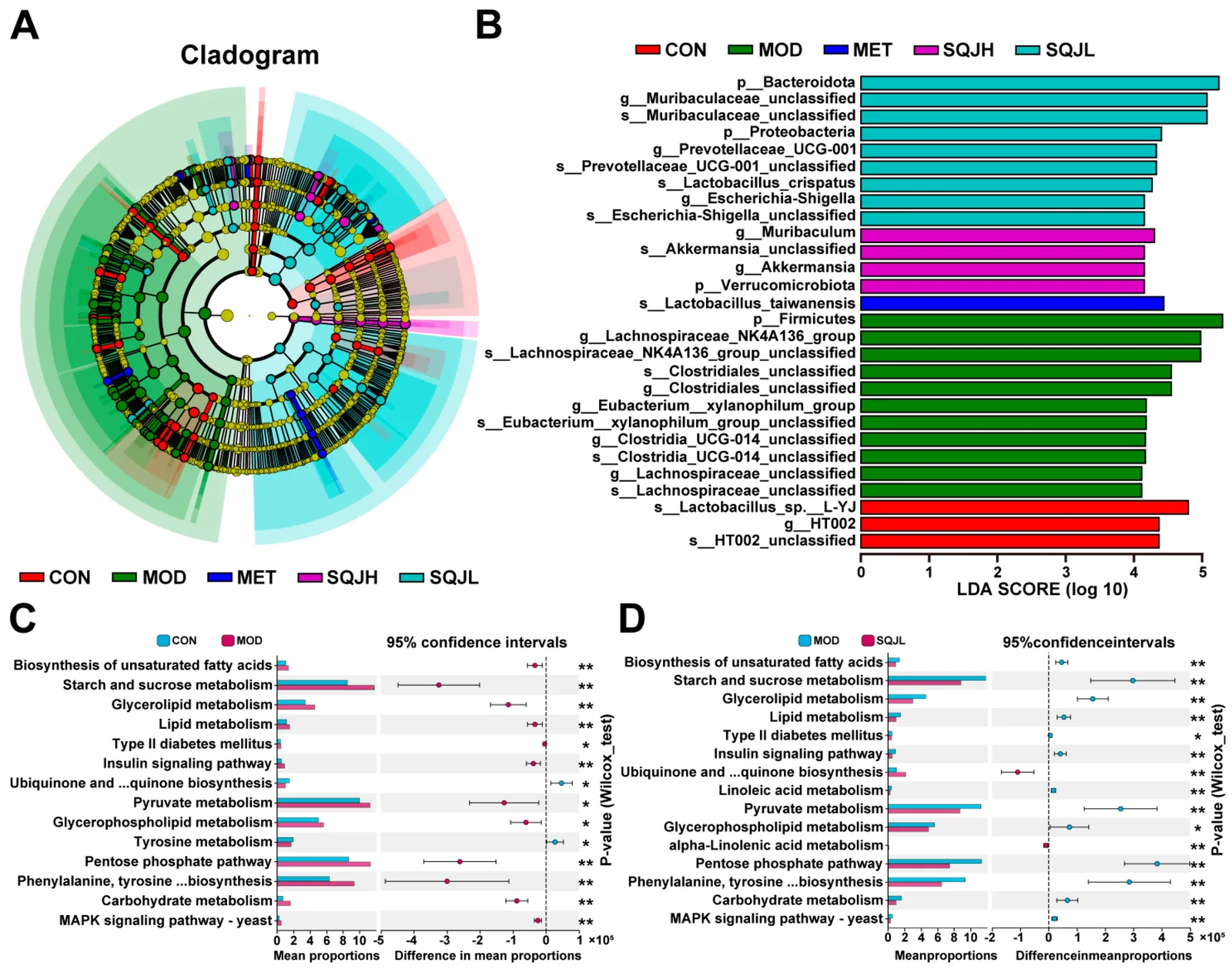
Regulatory effects of SQJ botanical extract on microbial taxonomic biomarkers and predicted functional pathways in diabetic mice. (**A**) LEfSe cladogram showing microbial taxonomic biomarkers enriched in different groups. (**B**) Linear discriminant analysis (LDA) score plot of discriminative bacterial taxa. (**C**) Differences in predicted microbial functional pathways between the CON and MOD groups, shown as mean proportions, differences in mean proportions, 95% confidence intervals, and Wilcoxon test *p* values. (**D**) Differences in predicted microbial functional pathways between the MOD and SQJL groups, shown using the same analytical format as in (**C**). * *p* < 0.05, ** *p* < 0.01.

LEfSe analysis further identified group-specific microbial biomarkers after STZ induction and SQJ intervention (Figure 9A,B). The SQJL group was characterized by enrichment of Bacteroidota-, *Muribaculaceae_unclassified-*, *Prevotellaceae_UCG-001-*, *Lactobacillus_crispatus-*, and *Escherichia-Shigella*-related taxa. The SQJH group was associated with *Muribaculum-*, *Akkermansia-*, Verrucomicrobiota-, and *Lactobacillus taiwanensis*-related signals, whereas the MOD group was mainly characterized by Firmicutes-, *Lachnospiraceae_NK4A136_group-*, *Clostridiales-*, and *Eubacterium_xylanophilum_group*-related taxa.

PICRUSt2 analysis was conducted to explore the predicted functional potential of the gut microbial communities. STZ-induced diabetes was associated with differences in the predicted relative abundance of pathways related primarily to lipid and carbohydrate metabolism, glycerophospholipid metabolism, and aromatic amino acid metabolism (Figure 9C). Compared with the MOD group, the SQJL group showed differences in a similar set of predicted metabolic pathways (Figure 9D). These findings suggest potential differences in the functional capacity encoded by the gut microbial communities. However, because these functional profiles were inferred from 16S rRNA gene data, they should not be interpreted as direct evidence of pathway activation, metabolic flux, or microbial metabolic activity.

**Figure 10:**
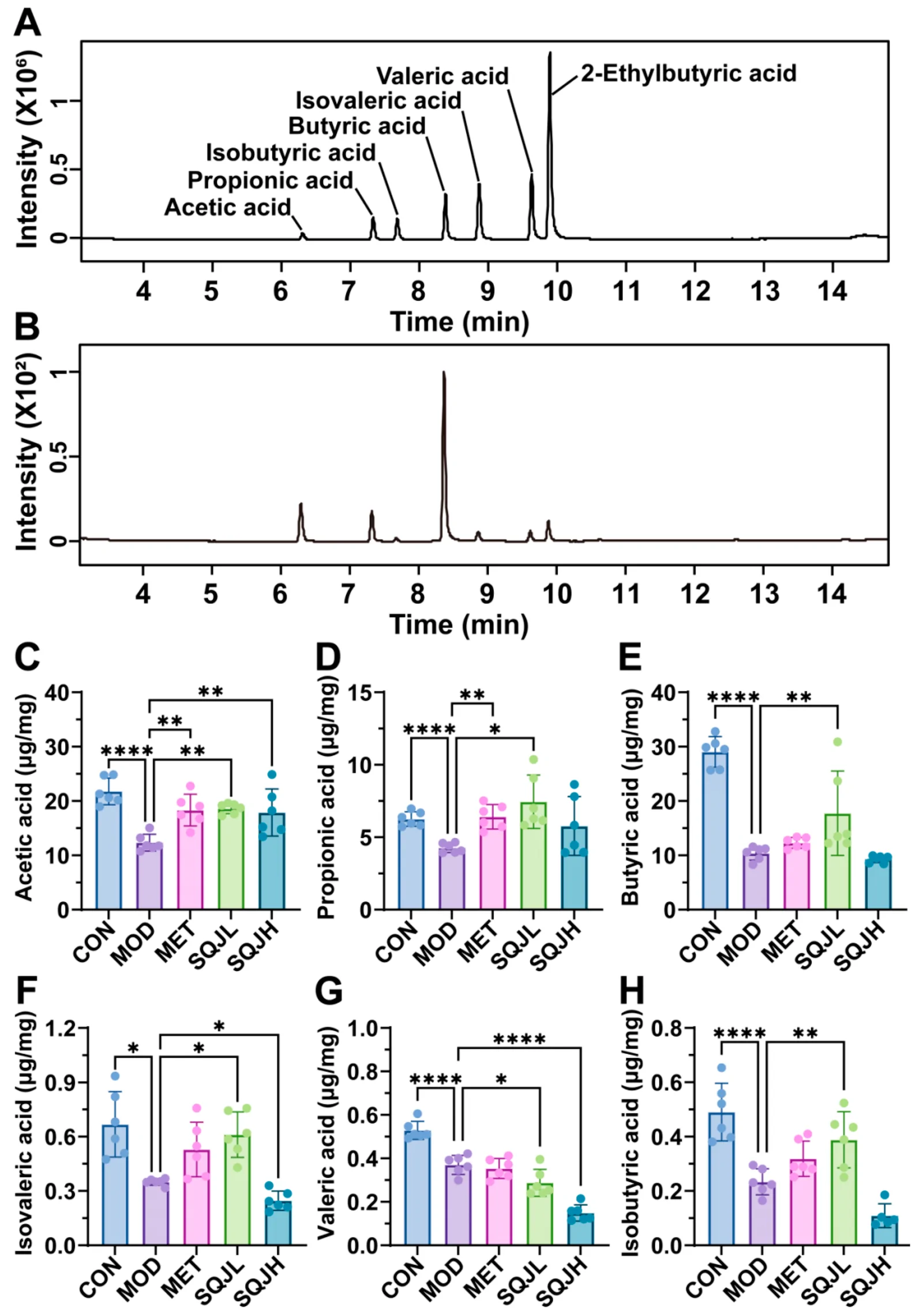
Effects of SQJ botanical extract on SCFA levels in colonic contents of diabetic mice. (**A**) Representative chromatogram of mixed short-chain fatty acid (SCFA) standards, including acetic acid, propionic acid, isobutyric acid, butyric acid, isovaleric acid, valeric acid, with 2-ethylbutyric acid as the internal standard. (**B**) Representative chromatogram of SCFAs in colonic contents. (**C**–**H**) Concentrations of acetic acid (**C**), propionic acid (**D**), butyric acid (**E**), isovaleric acid (**F**), valeric acid (**G**), and isobutyric acid (**H**). Data are presented as mean ± SD (*n* = 6). Normality was assessed using the Shapiro–Wilk test. Statistical comparisons were performed using one-way ANOVA followed by Dunnett’s post hoc test. * *p* < 0.05, ** *p* < 0.01, **** *p* < 0.0001 vs. MOD.

The GC-MS method showed good linearity and acceptable precision, repeatability, and stability for the six SCFAs (Tables S4 and S5). Representative chromatograms of mixed SCFA standards and colonic content samples are shown in Figure 10A,B. Compared with the CON group, the MOD group showed significantly lower acetic acid, propionic acid, butyric acid, isovaleric acid, valeric acid, and isobutyric acid levels (Figure 10C–H)**.** SQJL significantly increased acetic acid, propionic acid, butyric acid, isovaleric acid, and isobutyric acid relative to the MOD group, whereas SQJH significantly increased acetic acid but showed a weaker or distinct pattern for the other SCFAs (Figure 10C–H). Thus, SQJ partially restored colonic SCFA profiles, with a stronger response for several SCFAs at lower exposure. This non-monotonic pattern suggests a dose-sensitive ecological response rather than a simple linear dose–response relationship. HPD and GSTM3, prioritized by the integrated analyses, were subsequently examined in the liver together with oxidative-stress markers.

### 2.6. SQJ Normalizes Hepatic HPD/GSTM3 Responses and Improves Hepatic Redox Status

**Figure 11:**
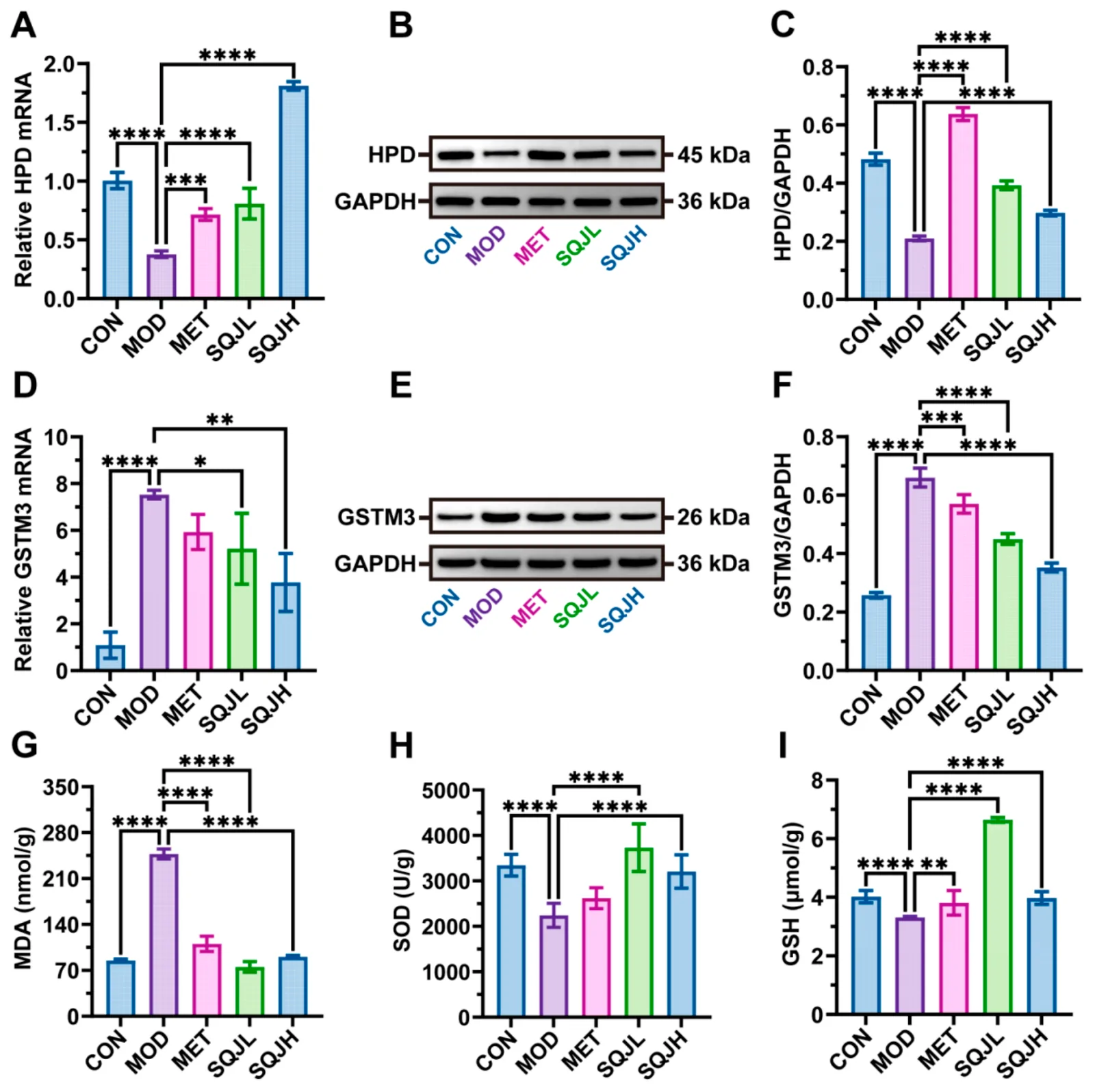
Effects of SQJ botanical extract on hepatic HPD/GSTM3 responses and oxidative stress markers in STZ-induced diabetic mice. (**A**–**C**) Hepatic HPD mRNA and protein expression, including relative HPD mRNA expression normalized to β-actin (**A**), representative Western blot images of HPD and GAPDH (**B**), and quantification of HPD protein expression normalized to GAPDH (**C**). (**D**–**F**) Hepatic GSTM3 mRNA and protein expression, including relative GSTM3 mRNA expression normalized to β-actin (**D**), representative Western blot images of GSTM3 and GAPDH (**E**), and quantification of GSTM3 protein expression normalized to GAPDH (**F**). (**G**–**I**) Hepatic oxidative stress markers, including malondialdehyde level (MDA, (**G)**), superoxide dismutase activity (SOD, (**H)**), and glutathione level (GSH, (**I)**). HPD expression was decreased in the MOD group and partially restored after SQJ treatment, whereas GSTM3 expression was increased in the MOD group and normalized after SQJ treatment. MDA, SOD, and GSH were expressed per gram of liver tissue. Data are presented as mean ± SD. For qPCR analyses (**A**,**D**), *n* = 3 independent biological replicates randomly selected from each group before analysis; for Western blot quantification (**C**,**F**), *n* = 4 independent biological replicates randomly selected from each group before analysis; technical qPCR triplicates were not treated as independent biological replicates; for oxidative stress markers (**G**–**I**), *n* = 10 independent biological replicates. Normality was assessed using the Shapiro–Wilk test. Statistical comparisons were performed using one-way ANOVA followed by Dunnett’s post hoc test. * *p <* 0.05, ** *p <* 0.01, *** *p <* 0.001, **** *p <* 0.0001 vs. MOD.

HPD and GSTM3 were selected for experimental follow-up using predefined, complementary evidence rather than network rank alone. HPD was prioritized because it was directly connected to the altered tyrosine-related metabolites in the CREG network, showed a comparatively large expression change in GSE156035 (log_2_FC = −1.058), and remained significant after BH correction (adjusted *p* = 0.027), although this change was not reproduced in GSE55098. GSTM3 was prioritized as a GST-family/redox-associated network node that was biologically consistent with the hepatic oxidative-stress endpoints measured in this study; however, it was not a significant DEG in either human transcriptomic dataset and was therefore treated as a network-derived candidate rather than transcriptomically validated evidence (Table S7). In the targeted, small-subset follow-up analyses shown in Figure 11A–C, hepatic HPD mRNA and protein levels were decreased in the MOD group compared with the CON group (*p* < 0.0001), and SQJ botanical extract partially restored HPD expression. In the corresponding randomly selected subsets, hepatic GSTM3 mRNA and protein expression was increased in the MOD group and reduced by metformin and SQJ botanical extract (Figure 11D–F). Given the limited biological replicate numbers, these molecular results are interpreted as supportive evidence of directionally consistent treatment-associated responses rather than definitive validation of HPD or GSTM3 as causal mediators.

Hepatic oxidative stress markers were also altered after STZ induction and SQJ botanical extract treatment. Compared with the CON group, the MOD group showed increased MDA levels and decreased SOD activity and GSH levels, indicating enhanced hepatic oxidative stress (*p* < 0.0001; Figure 11G–I). Compared with the MOD group, SQJ botanical extract reduced MDA and increased SOD activity and/or GSH in an indicator-specific pattern, with significant differences marked in Figure 11G–I. Together, targeted hepatic assessment showed SQJ-responsive normalization of HPD/GSTM3 expression and improved hepatic redox status. The omics-prioritization-to-validation sequence supports an HPD/GSTM3-associated hepatic redox adaptation, although these proteins should be interpreted as SQJ-responsive host nodes rather than confirmed causal mediators.

## 3. Discussion

The principal finding of this study is that a chemically characterized SQJ botanical extract derived from five edible-medicinal botanicals improved glucose homeostasis while producing coordinated changes in intestinal microbial and SCFA profiles, circulating metabolites, and hepatic redox status in STZ-induced diabetic mice. UHPLC-Orbitrap MS/MS putatively annotated 250 constituents and established a composition-linked identity for this complex botanical matrix [14,16]. SQJ reduced fasting blood glucose, improved glucose tolerance and insulin levels, attenuated inflammatory dysregulation, and alleviated pancreatic, hepatic, and intestinal injury. These responses are relevant to the systemic nature of diabetes-associated metabolic dysfunction, which involves gut microbial and inflammatory perturbations [33,34] and inter-organ metabolic crosstalk [35,36]. The multi-layer analyses were therefore used to resolve coordinated response patterns across the circulation, gut microbiota, and liver rather than to force a single-target explanation [37,38]. Hepatic oxidative-stress changes were interpreted within this broader metabolic context [39,40,41]. The STZ model remains a model of β-cell injury and hyperglycemic stress and does not reproduce the full autoimmune complexity of human T1DM [42,43].

The chemical composition of SQJ provides an important upstream context for interpreting these distributed responses. The putatively annotated profile comprised multiple constituent classes, including iridoid glycosides, flavonoids and isoflavonoids, triterpenoid and steroidal saponins, phenolic and other organic acids, amino acids, nucleosides, and saccharide- and oligosaccharide-related constituents. Independent quantification of morroniside, loganin, calycosin-7-O-glucoside, and astragaloside IV further provided quantitative reference points for the reproducibility of this multicomponent matrix. Previous studies of individual SQJ component botanicals or their constituents provide relevant pharmacological context. Notably, loganin, morroniside, and ursolic acid isolated from Corni Fructus have been reported to exert combined hypoglycemic effects [10], while Dioscorea polysaccharides have been associated with improved insulin resistance and gut microbial remodeling [11]. Bioactive constituents from Polygonatum and polysaccharide-related fractions from Astragalus have likewise been associated with antidiabetic or metabolic activities [12,13]. Collectively, these observations make it plausible that the biological effects of SQJ reflect additive or interactive contributions from multiple constituent classes rather than the action of a single dominant compound. However, constituent–constituent synergy was not directly tested in the present study and should therefore be regarded as a pharmacological hypothesis rather than a demonstrated mechanism.

At the same time, the chemical profile should not be mapped directly onto individual endogenous metabolic changes. The coexistence of chemically distinct SQJ constituents defines a complex oral exposure capable of interacting with both intestinal and host metabolic systems. In this context, the observed cecal microbial restructuring and SCFA changes, together with partial normalization of aromatic amino acid metabolism, lysophospholipid remodeling, α-linolenic acid metabolism, PUFA-derived oxylipins, and hepatic redox status, are biologically compatible with a multicomponent botanical response. However, the present study did not perform serum pharmacochemistry, absorption profiling, isotope tracing, or constituent-specific intervention experiments. Individual SQJ-derived compounds therefore cannot be assigned as causal drivers of specific endogenous metabolites or downstream biological endpoints. We consequently interpret the relationship between the chemical and biological datasets at the level of a multicomponent exposure–response framework rather than as a direct one-to-one constituent–metabolite mechanism.

At the systemic level, serum metabolomics captured the metabolic response accompanying SQJ intervention [44,45]. The dominant SQJ-responsive patterns involved aromatic amino acid-related metabolites, lysophospholipids, α-linolenic acid metabolism, and PUFA-derived oxylipins, metabolite classes closely linked to oxidative stress, inflammatory lipid signaling, and membrane lipid remodeling [46,47]. Human and experimental studies likewise associate amino acid and phospholipid signatures with diabetes and metabolic risk [48,49]. However, because the serum metabolites were putatively annotated without confirmation using authentic reference standards, individual assignments—particularly those of oxylipins and structurally related lipid species—should be interpreted cautiously. Positional and stereochemical isomers cannot always be distinguished unambiguously using accurate mass and routine MS/MS data alone. Rather than treating individual serum metabolites as definitively identified upstream drivers, we interpret the circulating metabolome as an intermediate systemic readout that integrates endocrine, inflammatory, hepatic, and gut-derived metabolic inputs [50,51]. The partial normalization of these signatures therefore places aromatic amino acid and lipid remodeling within the broader SQJ-associated phenotypic recovery.

Building on the circulating metabolic layer, two public peripheral-blood T1DM transcriptomic datasets were analyzed independently [52,53]. BH correction identified 311 genes in GSE156035 but none in GSE55098 at |log_2_FC| > 0.585 and adjusted *p* < 0.05. Although 12 genes met the exploratory raw-*p*-value and fold-change thresholds in both datasets, only 11 were directionally concordant and none was FDR significant in both. This limited replication likely reflects differences in platform, cohort composition, biological heterogeneity, and statistical power. The blood-derived results were therefore retained as dataset-specific and cross-dataset consistency evidence, rather than merged into a permissive union or interpreted as liver-specific findings. Metscape mapping and the compound–target network were used as supportive, hypothesis-generating layers [24,54]. Such integration can prioritize nodes for experimental follow-up [55,56], but cannot demonstrate direct molecular binding or causal target engagement. The 33-gene supplementary network was retained as an exploratory representation of the original nominal-threshold workflow and was not treated as FDR-level or cross-dataset replicated transcriptomic evidence. Accordingly, the integrated results were used to focus hepatic validation on HPD/GSTM3 and oxidative-stress responses [47,57].

In parallel, the cecal microbiota and colonic SCFAs defined the intestinal component of the SQJ response. Orally consumed botanical matrices can interact with the gut microbial ecosystem, and multicomponent plant interventions frequently alter community structure and microbial metabolic output [58,59]. SQJ shifted the diabetes-associated cecal community and altered several bacterial taxa identified in the present dataset. PICRUSt2 predicted differences in microbial functions related to carbohydrate, lipid, glycerophospholipid, and tyrosine-associated pathways [60]. In parallel, STZ reduced multiple colonic SCFAs, whereas SQJ, particularly at lower exposure, partially restored acetate, propionate, butyrate, isovalerate, and isobutyrate. SCFAs are microbial metabolites with established links to insulin sensitivity, immune regulation, and host metabolism [61,62]. Notably, the broader recovery of several SCFAs at the lower SQJ exposure contrasted with the stronger glucose- and insulin-related responses observed at the higher exposure. This divergence argues against a simple monotonic SCFA-mediated explanation of SQJ efficacy and suggests that the intestinal response represents one component of a broader microbial–host metabolic response. Non-monotonic ecological responses to complex dietary substrates are biologically plausible [63]. Nevertheless, SCFA restoration should be viewed as an accompanying microbial-metabolic response, not proof that SCFAs mediated the glycemic effects of SQJ [64,65]. The concurrence of microbial, SCFA, and systemic phenotypic changes supports biological coherence [66,67], but direct causal testing is required.

The integrated analyses directed targeted hepatic assessment toward HPD and GSTM3 through distinct lines of evidence [68,69,70]. HPD had direct pathway relevance to the altered tyrosine-related metabolites, a large expression decrease in GSE156035, and FDR support in that dataset, but it was not altered in GSE55098; its human blood transcriptomic support should therefore be regarded as dataset specific. HPD catalyzes the conversion of 4-hydroxyphenylpyruvate to homogentisate in tyrosine catabolism. In the present mouse study, hepatic HPD expression was suppressed in diabetic mice and partially restored after SQJ treatment, consistent with improvement of diabetes-associated disturbance in aromatic amino acid handling [71,72]. GSTM3, by contrast, was selected because of its GST-family/redox network position and biological consistency with the measured oxidative-stress indices, not because it was a transcriptomic DEG. GSTM3 showed increased hepatic expression in diabetic mice and reduction after SQJ treatment. Together with lower MDA and recovery of SOD activity and GSH levels, this pattern is consistent with attenuation of hepatic oxidative/electrophilic stress [73,74]. These mouse data provide experimental support for the prioritized candidates, but do not establish that either protein is a direct SQJ target or causally required.

**Figure 12:**
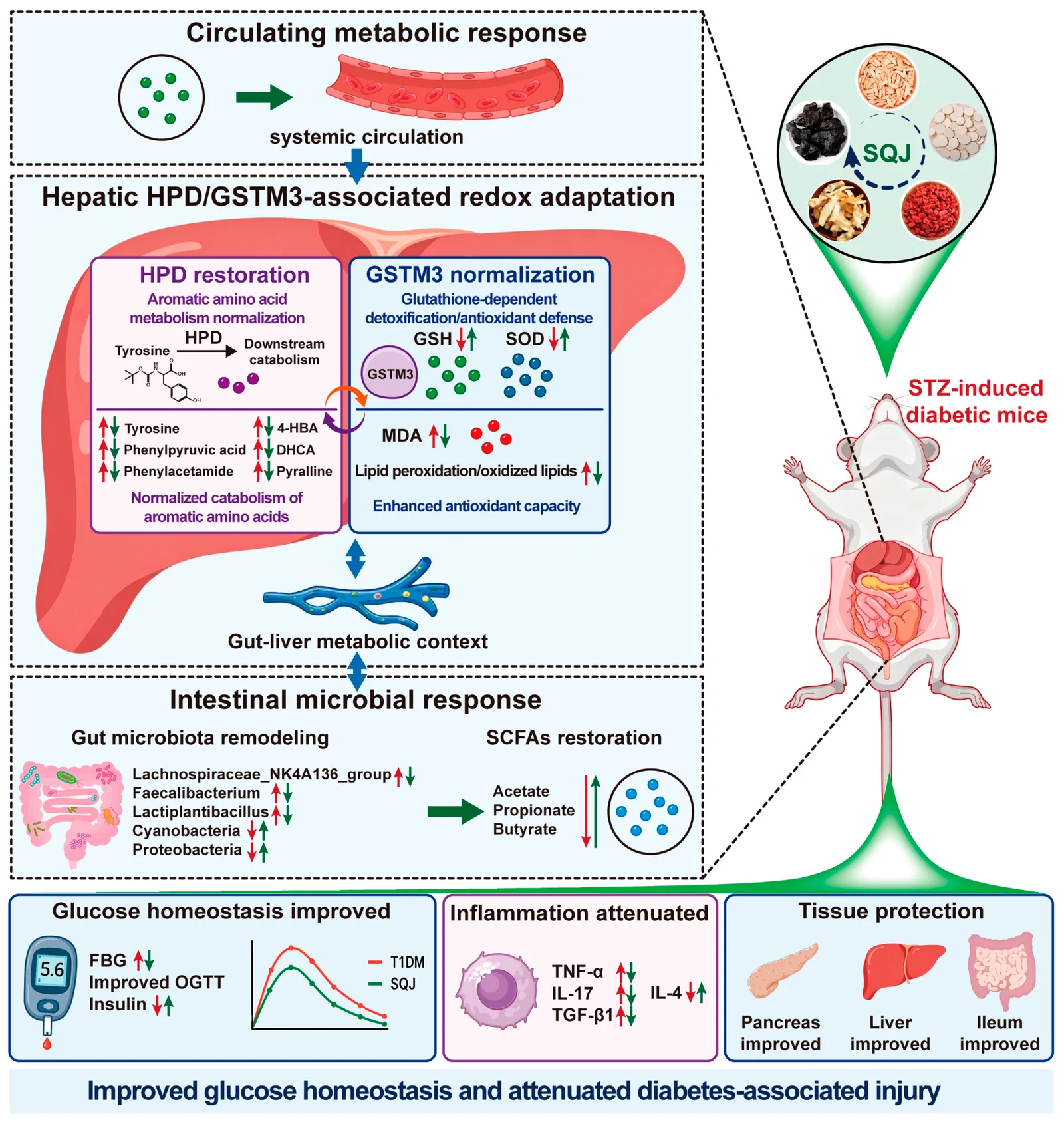
Descriptive integrated microbial and host metabolic response framework associated with SQJ intervention in STZ-induced diabetic mice. The schematic summarizes coordinated intestinal microbial, circulating metabolic, and hepatic redox responses observed after SQJ intervention in STZ-induced diabetic mice. SQJ intervention was accompanied by cecal microbial restructuring, colonic SCFA changes, partial normalization of serum amino acid- and lipid-related metabolites, restoration of hepatic HPD expression, normalization of diabetes-associated GSTM3 upregulation, reduced MDA, and recovery of SOD activity and GSH levels. These responses were associated with improved glucose homeostasis, attenuated inflammatory dysregulation, and reduced pancreatic, hepatic, and ileal tissue injury. Red arrows indicate diabetes-associated changes in the MOD group relative to the CON group, whereas green arrows indicate SQJ-associated reversal or normalization trends relative to the MOD group. This framework is descriptive and does not imply causal directionality among response layers, direct microbial mediation, or a linear gut-to-liver mechanism.

Viewed together, the findings are consistent with a distributed microbial–host metabolic response pattern to the chemically complex SQJ botanical matrix rather than a single-target or linear gut-to-liver mechanism [13,75]. In this framework, cecal microbiota and SCFA changes provide an intestinal metabolic context [76,77], serum metabolomics captures a circulating intermediate phenotype [78,79], and HPD/GSTM3-associated molecular responses together with redox measurements provide a targeted hepatic redox response layer [47,68]. The convergence of these layers indicates that SQJ intervention was accompanied by microbial restructuring, altered SCFA availability, systemic amino acid and lipid remodeling, and hepatic redox adaptation. These coordinated intestinal, circulating metabolic, and hepatic response layers are summarized in the proposed integrated microbial and host metabolic response framework (Figure 12). Importantly, this is an integrated response model; it does not establish a linear gut-to-circulation-to-liver pathway or the direction of information flow among these compartments.

Several limitations define the boundary of this interpretation. First, the STZ-induced mouse model captures β-cell injury and hyperglycemic stress but does not reproduce all immunological features of human T1DM, and only male mice were studied. Second, histopathology and the targeted HPD/GSTM3 analyses used small randomly selected subsets. Histological sections were evaluated blinded to group allocation, but no prespecified quantitative scoring system was applied. Accordingly, the histological findings are qualitative, and the HPD/GSTM3 results should be regarded as supportive, exploratory validation that requires confirmation in larger independent cohorts. Third, the lower SQJ exposure served as the lower HED-based reference exposure, whereas the threefold higher dose was included to examine the upper response range and should not be interpreted as habitual dietary intake. Fourth, residual chitosan in the final extract was not quantitatively determined; because chitosan was used as a clarification aid and the precipitated fraction was removed before drying, a contribution from residual chitosan cannot be completely excluded. Fifth, the gut microbiota analysis included six animals per group and therefore represented a subset of the experimental animals. Although samples were selected before sequencing according to the predefined sample order and not on the basis of downstream outcomes, the relatively small sample size may limit statistical power and generalizability. PICRUSt2 profiles are predictions based on 16S rRNA gene data and do not directly measure microbial gene expression, protein abundance, metabolite production, metabolic flux, or pathway activity. Sixth, serum metabolite annotations were not confirmed using authentic reference standards, limiting definitive positional or stereochemical assignment, particularly for oxylipins and closely related lipid species. Seventh, because serum was collected 1 h after the final administration, acute post-dose effects and potentially SQJ-derived circulating features cannot be completely excluded. Eighth, microbial composition was profiled in cecal contents whereas SCFAs were quantified in colonic contents, so these spatially distinct datasets should not be interpreted as direct taxon–metabolite pairings. Ninth, the two public transcriptomic datasets showed limited FDR-level replication; no gene met the prespecified FDR and fold-change criteria in both datasets. Thus, the transcriptomic and network analyses are association- and prioritization-based, HPD has dataset-specific rather than replicated human transcriptomic support, and GSTM3 remains a network-derived candidate [24]. Tenth, molecular networking was not performed in the present study. Future GNPS-based MS/MS spectral networking could further organize the annotated SQJ features into structurally related molecular families and complement the current literature-curated annotation workflow. Finally, causal requirements for microbiota-derived SCFAs, HPD, and GSTM3 were not tested. Future studies should use larger independent cohorts, direct microbial/metabolite perturbation, and functional testing of HPD/GSTM3 to resolve directionality and causal requirements.

## 4. Materials and Methods

### 4.1. Botanical Materials, Animals, and Ethics

Astragali Radix (AR, the dried root of *Astragalus membranaceus* (Fisch.) Bge. var. *mongholicus* (Bge.) Hsiao), Corni Fructus (CF, the dried ripe sarcocarp of *Cornus officinalis* Sieb. et Zucc.), Dioscoreae Rhizoma (DR, the dried rhizome of *Dioscorea opposita* Thunb.), Polygonati Rhizoma (PR, the dried rhizome of *Polygonatum sibiricum* Red.), and Polygonati Odorati Rhizoma (POR, the dried rhizome of *Polygonatum odoratum* (Mill.) Druce.) were purchased from Anguo Yaoyuan Trading Co., Ltd. (Baoding, China). All botanical materials were authenticated by Prof. Xiangping Pei (Shanxi University of Chinese Medicine). Voucher specimens were deposited in the Herbarium of Shanxi University of Chinese Medicine with accession numbers HQ-20230526 (AR), SZY-20230526 (CF), SY-20230526 (DR), HJ-20230526 (PR), and YZ-20230526 (POR).

Healthy Kunming mice weighing 18–22 g were supplied by SPF (Beijing) Biotechnology Co., Ltd. (Beijing, China; License No.: SCXK (Jing) 2019-0010). The animals were maintained under standard laboratory conditions at 24–26 °C and 55–65% relative humidity, with a 12 h light/dark cycle and ad libitum access to food and water. The experimental protocol was reviewed and approved by the Animal Ethics Committee of Shanxi University of Chinese Medicine (Approval No. 2021DW219). All animal experiments were performed in accordance with the Guide for the Care and Use of Laboratory Animals published by the U.S. National Research Council [80].

### 4.2. Preparation of the SQJ Botanical Extract

The SQJ botanical extract was prepared from AR, DR, CF, PR, and POR. The five botanical materials were combined at an equal weight ratio of 1:1:1:1:1, with 12 g of each material contributing to a 60 g botanical blend; the preparation was proportionally scaled according to experimental requirements. The mixed materials were extracted three times with 12 volumes of distilled water for 60, 40, and 40 min, respectively. The three aqueous extracts were combined and concentrated to a botanical material-to-liquid ratio of 1:10. For clarification, a 1% chitosan solution was prepared by dissolving 1.0 g of chitosan in 100 mL of 1% acetic acid and was added to the concentrated extract at an amount equivalent to 0.2 g chitosan per litre of extract. The mixture was stirred for 5 min, allowed to stand at room temperature for 30 min, and centrifuged at 4000 rpm for 15 min. Chitosan was used only as a clarification aid, and the precipitated fraction was removed by centrifugation before concentration and drying of the clarified supernatant. The clarified supernatant was collected, concentrated under reduced pressure to a thick extract, and dried at low temperature to obtain the SQJ botanical extract. The final extraction yield was approximately 45%, with 60 g of the botanical blend yielding approximately 27 g of dried extract.

### 4.3. Chemical Characterization of the SQJ Botanical Extract

An appropriate amount of SQJ botanical extract was accurately measured and diluted to volume with 50% methanol in a 25 mL volumetric flask. Then, 1 mL of the resulting solution was transferred and diluted with three volumes of 50% methanol. The diluted solution was filtered through a 0.22 μm microporous membrane, and the subsequent filtrate was collected for UHPLC-MS/MS analysis.

Qualitative analysis of SQJ botanical extract was performed using a Vanquish UHPLC system coupled to an Orbitrap Exploris™ 480 high-resolution mass spectrometer (Thermo Fisher Scientific, Germany). Chromatographic separation was carried out on a Hypersil GOLD C18 column (100 mm × 2.1 mm, 1.9 μm; Thermo Fisher Scientific, USA). The column temperature was maintained at 40 °C, the flow rate was set at 0.2 mL/min, and the injection volume was 2 μL. The mobile phase consisted of 0.1% formic acid in water (A) and methanol (B). Gradient elution was performed as follows: 0–1.5 min, 2% B; 1.5–3 min, 2–85% B; 3–10 min, 85–100% B; 10–10.1 min, 100–2% B; and 10.1–12 min, 2% B. Mass spectrometric detection was performed using an electrospray ionization source in both positive and negative ion modes. The full-scan mass range was set at *m/z* 100–1500. Full MS scans were acquired at a resolution of 60,000 FWHM, with an automatic gain control (AGC) target of 3 × 10^6^ and a maximum injection time of 100 ms. Data-dependent acquisition (DDA) was performed using a Top10 method. MS/MS spectra were acquired at a resolution of 15,000 FWHM, with an AGC target of 2 × 10^5^ and a maximum injection time of 25 ms. The precursor isolation width was set to 1.5 *m/z*. Stepped normalized collision energies (NCE) of 20, 40, and 60 were applied for HCD fragmentation. The apex trigger window was 2–12 s, and dynamic exclusion was set to 10.0 s. The S-lens RF level was set to 60, and the autosampler temperature was maintained at 4 °C.

To facilitate chemical characterization of the SQJ botanical extract, a literature-curated in-house reference database was established by collecting previously reported constituents of the five botanical components of SQJ from published LC–MS studies, together with their molecular formulae, theoretical accurate masses, reported precursor/adduct ions, and available MS/MS fragmentation information. This database was used as a literature-derived reference resource rather than as an authentic-standard spectral library. Raw mass spectrometry data were converted to mzXML format using MSConvert 3.0.26057-78b3a0d and processed with MZmine v4.1.7 for peak detection, peak-area integration, and alignment.

Detected features were initially matched against the literature-curated database using a mass-tolerance window of 10 ppm. Candidate annotations were subsequently evaluated using measured accurate mass, plausible precursor/adduct assignment, diagnostic MS/MS fragmentation patterns, retention behavior, reported botanical occurrence, and literature-reported mass-spectrometric evidence. Re-evaluation of the final annotation dataset showed that all retained constituents had absolute precursor-mass errors within 5 ppm. No fixed minimum number of matching fragment ions or numerical matching-score threshold was applied; instead, fragment evidence was assessed according to the consistency of diagnostically informative product ions and overall fragmentation behavior with published MS/MS data.

Authentic reference standards were not analyzed under the same UHPLC-Orbitrap MS/MS profiling conditions; therefore, authentic-standard retention-time matching and reference-standard MS/MS spectral matching were not used for these profiling annotations. Accordingly, 249 constituents supported by diagnostic MS/MS evidence were reported as putative MSI Level 2 annotations according to the Metabolomics Standards Initiative [81]. One constituent (No. 38, 4-guanidinobutanoate), for which diagnostic MS/MS fragment information was not available, was conservatively classified as an MSI Level 3 tentative assignment rather than Level 2. Representative literature-supported marker constituents from the five botanical components were highlighted in the total ion chromatograms as chemical reference features and were not interpreted as evidence of direct bioactivity.

### 4.4. Quantitative Determination of Representative Marker Constituents in SQJ

To further improve the chemical standardization of the SQJ botanical extract, four representative quality-control marker constituents were quantitatively determined using authentic reference standards. Morroniside, loganin, and calycosin-7-O-glucoside were analyzed by UPLC-UV, whereas astragaloside IV was determined separately by HPLC-ELSD.

For UPLC-UV analysis, approximately 2 g of accurately weighed dried SQJ extract was dissolved in 50% methanol and diluted to 50 mL. The solution was filtered through a 0.22-μm membrane, and the subsequent filtrate was used for analysis. Chromatographic separation was performed on an ACQUITY UPLC BEH C18 column (2.1 × 100 mm, 1.7 μm; Waters) maintained at 30 °C. The mobile phases consisted of acetonitrile (A) and 0.2% formic acid in water (B). The gradient program was as follows: initial, 10% A/90% B; 9.0 min, 60% A/40% B; and 10.0 min, 10% A/90% B. The flow rate was 0.2 mL/min, UV detection was performed at 240 nm, and the injection volume was 5 μL. Morroniside, loganin, and calycosin-7-O-glucoside were quantified using external-standard calibration curves.

For HPLC-ELSD determination of astragaloside IV, approximately 2 g of accurately weighed dried SQJ extract was dissolved in 20 mL of water and extracted four times with water-saturated n-butanol (20 mL each). The combined n-butanol fractions were washed twice with ammonia solution (40 mL each). The n-butanol layer was collected and evaporated to dryness in a water bath, and the residue was reconstituted and diluted to 2 mL with 50% methanol. After filtration through a 0.22 μm membrane, the subsequent filtrate was used for analysis. Chromatographic separation was performed on an Agela Venusil C18 column (250 × 4.6 mm, 5 μm) maintained at 30 °C. Acetonitrile–water (36:64, v/v) was used as the mobile phase at a flow rate of 1.0 mL/min, and the injection volume was 10 μL. The ELSD drift-tube temperature was set to 105 °C, the gas flow rate was 2.8 L/min, and the detector gain was set to 1. Because of the nonlinear response characteristics of ELSD, the calibration curve for astragaloside IV was constructed by linear regression of the natural logarithm of peak area against the natural logarithm of concentration, yielding the equation ln A = 1.5723 ln C + 5.5022 (R^2^ = 0.9990). The contents of all four marker constituents were expressed as mg/g dried SQJ extract.

### 4.5. Experimental Design, Treatment Regimen, and Dose Rationale

After one week of acclimatization, 60 healthy male Kunming mice were used for the animal experiment. According to body weight, 10 mice were randomly assigned to the control group (CON) and were not subjected to STZ modeling, while the remaining 50 mice were used to establish the STZ-induced diabetic model. After fasting for 12 h with free access to water, mice in the modeling cohort received a single intraperitoneal injection of freshly prepared 1% streptozotocin (STZ, 150 mg/kg). STZ was dissolved immediately before use in freshly prepared citrate buffer (pH 4.2–4.5). During the modeling period, all mice had free access to food and water. On day 3 after STZ injection, mice were fasted for 10 h, and blood samples were collected from the tail vein to determine fasting blood glucose (FBG) levels using a glucometer. Mice with FBG levels ≥11.1 mmol/L were considered successfully diabetic, and mice that failed to meet this criterion were excluded.

After successful establishment of the diabetic mouse model, 40 modeled mice were randomly divided into four groups (*n* = 10 per group): model group (MOD), metformin group (MET, 200 mg/kg), low-dose SQJ group (SQJL, 4.5 g/kg botanical extract), and high-dose SQJ group (SQJH, 13.5 g/kg botanical extract). All treatments were administered once daily by oral gavage for 28 days; CON and MOD mice received an equal volume of distilled water. Dose translation was evaluated by body-surface-area scaling using HED (g/kg) = mouse dose (g/kg) × 3/37. For a 60 kg adult, the SQJL dose corresponds to approximately 21.9 g/day of dried extract and, based on the 45% extraction yield, approximately 48.6 g/day of the original equal-ratio five-botanical blend (about 9.7 g/day of each material). The SQJH dose corresponds to approximately 65.7 g/day of dried extract or 146.0 g/day of the original blend (about 29.2 g/day of each material). The lower dose served as the lower HED-based reference exposure, whereas the threefold higher dose was included to assess the upper response range and was not intended to represent habitual dietary intake. Randomization was completed before treatment administration. Failure to meet the prespecified FBG threshold was the animal-level exclusion criterion applied before allocation to the diabetic treatment groups; samples used for the subset analyses were not selected on the basis of downstream assay results.

### 4.6. Physiological, Biochemical, and Histopathological Assessments

Body weight and fasting blood glucose (FBG) levels were measured on days 0, 7, 14, 21, and 28 after treatment administration to evaluate general physiological condition and glycemic status. Before each measurement, mice were fasted for 6 h with free access to water. Body weight was recorded first, followed by FBG determination using blood collected from the tail tip and immediately analyzed with a glucometer. After 25 days of continuous treatment, an oral glucose tolerance test (OGTT) was performed. Mice were fasted for 12 h with free access to water and then orally administered glucose solution at a dose of 2.0 g/kg. Blood glucose levels were measured at 0, 0.5, 1, and 2 h after glucose loading. The OGTT-derived area under the curve (OGTT-AUC) was calculated as follows: OGTT-AUC = (0.5C_A_ + C_B_ + C_C_ + 0.5C_D_) / 2, where C_A_, C_B_, C_C_, and C_D_ represent blood glucose concentrations at 0, 0.5, 1, and 2 h after glucose administration, respectively.

At 1 h after the final administration, mice were anesthetized and blood samples were collected from the retro-orbital venous plexus. Serum was separated by centrifugation at 3500 rpm for 15 min and stored at −80 °C. Mice were then euthanized according to the approved protocol, and the pancreas, liver, spleen, kidney, ileum, and duodenum were rapidly collected. Cecal contents were stored at −80 °C for gut microbiota analysis, and colonic contents were stored at −80 °C for SCFA analysis.

Serum insulin and inflammatory cytokines, including TNF-α, IL-4, IL-17, and TGF-β1, were measured using commercial ELISA kits according to the manufacturers’ instructions. Pancreatic tissues were weighed, homogenized in pre-cooled PBS at a fixed tissue-to-buffer ratio, and centrifuged; the supernatants were diluted consistently before measurement of the same cytokines. Pancreatic cytokine levels were expressed as pg/mL of standardized homogenate supernatant. ALT and AST levels were measured in serum and liver homogenates using commercial biochemical assay kits. Hepatic ALT and AST levels were normalized to the total protein concentration of the corresponding liver homogenates.

For histopathological examination, three mice from each group were randomly selected before tissue processing. Pancreatic samples were transversely sectioned at the middle region; one portion was fixed in 4% paraformaldehyde for H&E staining, and the remaining pancreatic tissue was stored at −80 °C for subsequent biochemical assays. Liver samples for H&E staining were collected from the same hepatic lobe and fixed in 4% paraformaldehyde, whereas the remaining liver tissues were snap-frozen and stored at −80 °C for subsequent biochemical and molecular analyses. Representative spleen, kidney, ileum, and duodenum samples were collected from the same anatomical regions across animals and fixed in 4% paraformaldehyde. Fixed tissue samples were dehydrated through a graded ethanol series, cleared in xylene, embedded in paraffin, sectioned, and stained with hematoxylin and eosin (*H&E*) according to standard histological procedures. Histopathological alterations in the pancreas, liver, ileum, duodenum, spleen, and kidney were observed under a light microscope. Slides were coded before evaluation, and the evaluator assessed tissue morphology without knowledge of group allocation. Histological assessment was qualitative; no prespecified semiquantitative scoring system was applied.

### 4.7. Sample Selection for Metabolomics and Gut Microbiota Analyses

The animal experiment initially included 10 animals per group. For serum metabolomic profiling, all available serum samples with sufficient sample volume and meeting the predefined analytical quality-control requirements were included, resulting in 8–10 independent biological replicates per group. Sample inclusion was determined before metabolomic data analysis and was independent of the resulting metabolic profiles or group-separation patterns. For gut microbiota analysis, six eligible samples per group were selected before sequencing according to the predefined sample order and sample availability. Sample selection was performed independently of the metabolomic or microbial profiles and before downstream statistical analysis.

### 4.8. Untargeted Serum Metabolomics

Untargeted metabolomic profiling of serum samples was performed using ultra-high-performance liquid chromatography coupled with high-resolution mass spectrometry (UHPLC-HRMS). Serum samples were thawed in an ice-water bath prior to pretreatment. Briefly, 100 μL of serum was aliquoted and mixed with 300 μL of pre-chilled methanol to precipitate proteins. The mixture was vortexed for 1 min and centrifuged at 13,000 rpm for 15 min at 4 °C. The supernatant was collected and evaporated to dryness under a gentle stream of nitrogen. The dried residue was reconstituted in 200 μL of methanol, vortexed for 3 min, and centrifuged again at 13,000 rpm for 15 min at 4 °C. The resulting supernatant was collected for LC-MS analysis. Pooled quality control (QC) samples were prepared by combining equal aliquots from all serum samples and injected at regular intervals throughout the analytical sequence to monitor system stability and data reproducibility.

The chromatographic separation was performed on an ACQUITY UPLC BEH C18 column (2.1 mm × 100 mm, 1.7 μm). The column temperature was maintained at 40 °C, with a flow rate of 0.3 mL/min and an injection volume of 5 μL. The mobile phase consisted of acetonitrile (MS grade, Thermo Fisher Scientific, Lot No. 226049) as solvent A and 0.1% formic acid in water, prepared using formic acid (HPLC grade, Aladdin, Lot No. 220844), as solvent B. The gradient elution program was as follows: 0–0.5 min, 5% A; 0.5–1.5 min, 5–15% A; 1.5–4.5 min, 15–30% A; 4.5–6.0 min, 30–60% A; 6.0–9.0 min, 60–70% A; 9.0–12.0 min, 70–100% A; 12.0–13.0 min, 100% A; 13.0–13.5 min, 100–5% A; and 13.5–16.0 min, 5% A for column re-equilibration. The mass spectrometric detection was conducted using an electrospray ionization (ESI) source in both positive and negative ion modes. In positive ion mode, the spray voltage was set to 3.2 kV, with a sheath gas flow rate of 40 arb, an auxiliary gas flow rate of 5 arb, and an auxiliary gas heater temperature of 350 °C. In negative ion mode, the spray voltage was set to 2.5 kV, with a sheath gas flow rate of 38 arb, an auxiliary gas flow rate of 10 arb, and an auxiliary gas heater temperature of 300 °C. The ion transfer tube temperature was maintained at 320 °C, and the S-lens RF level was set to 50 V. Full MS and data-dependent MS/MS acquisition were performed in full MS/dd-MS2 mode over an m/z range of 100–1000. The resolutions were set to 70,000 FWHM for full MS and 17,500 FWHM for dd-MS2 acquisition, and the collision energy was set to 30 eV.

Raw LC-MS datasets were analyzed using Compound Discoverer software version 3.3 (Thermo Fisher Scientific, Waltham, MA, USA). The preprocessing workflow comprised peak picking, retention time adjustment, peak alignment, peak area integration, signal normalization, and metabolic feature extraction. The generated feature table was then exported for multivariate and univariate statistical analyses. PCA was performed to examine overall sample distribution, analytical performance, and clustering of QC injections. OPLS-DA was used as a complementary exploratory multivariate approach to characterize group-related metabolic variation rather than as a stand-alone criterion for differential-metabolite selection. Model fit and predictive performance were evaluated using *R^*2*^X*, *R^*2*^Y*, and *Q^*2*^* values together with 200-permutation tests. For the CON-versus-MOD comparison, differences in normalized peak areas were assessed using a two-sided unpaired Student’s t-test. To control the false discovery rate, raw *p*-values were adjusted using the Benjamini–Hochberg procedure across all eligible metabolic features included in the statistical analysis before differential-metabolite selection. Differential metabolites were defined using the combined criteria of *VIP* > 1 and Benjamini–Hochberg-adjusted *p* < 0.05. This procedure retained 24 differential metabolites. For descriptive comparisons of these metabolites across all experimental groups, the Kruskal–Wallis test followed by Dunn’s post hoc test was used. Serum metabolites were putatively annotated on the basis of accurate mass, isotope pattern, retention behavior, and, when available, MS/MS spectral matching against relevant spectral-library and database records, including HMDB. Because authentic reference standards were not used, the annotations were not considered definitive; in particular, positional and stereochemical assignments of oxylipins and closely related lipid species were interpreted cautiously unless supported by diagnostic MS/MS evidence. Pathway enrichment analysis of the putatively annotated differential metabolites was carried out using MetaboAnalyst 5.0 based on pathway information from the Kyoto Encyclopedia of Genes and Genomes (KEGG) database.

### 4.9. Clinical Transcriptomics and Metabolite–Gene Integration

Two T1DM-related human peripheral-blood transcriptomic datasets, GSE55098 and GSE156035, were obtained from the Gene Expression Omnibus (GEO) database to provide systemic disease context rather than liver-specific evidence. GSE55098 included 10 control and 12 T1DM samples and was analyzed from the RMA-normalized expression matrix; GSE156035 included 20 control and 20 T1DM samples and was analyzed from the quantile-normalized expression matrix. Probe identifiers were mapped to official gene symbols using the corresponding platform annotations. Probes with ambiguous gene assignments were excluded, and multiple probes mapping unambiguously to the same gene were summarized by their median expression.

Gene-level differential-expression analysis was performed separately for each dataset using a two-sided Welch’s t-test. Raw p values were adjusted by the Benjamini–Hochberg procedure across all annotated genes tested within each dataset. FDR-supported DEGs were defined by BH-adjusted *p* < 0.05 and |log_2_FC| > 0.585 (fold change > 1.5 or < 0.667). For sensitivity analysis only, genes meeting raw *p* < 0.05 and the same fold-change threshold were labeled nominal exploratory candidates and were not treated as FDR-confirmed DEGs. Cross-dataset consistency was assessed among the 19,599 genes measured in both datasets by evaluating overlap and direction of change. Dataset-specific lists were retained separately; no non-redundant union was used as evidence of replication. Volcano plots were generated to visualize the complete gene-level results.

Differential serum metabolites identified in Section 4.8 were mapped to reactions, enzymes, and genes using Cytoscape v3.8.2 with the Metscape plug-in. Gene identifiers and metabolite names were standardized before construction of the compound–reaction–enzyme–gene (CREG) network. The independently analyzed GEO results were overlaid as contextual evidence: FDR-supported genes from each dataset and directionally concordant exploratory signals were distinguished rather than pooled into a single DEG union. Candidate nodes were prioritized for experimental follow-up using explicit complementary criteria, including direct connectivity to altered metabolites, pathway relevance, transcriptomic effect magnitude and FDR support where present, cross-dataset consistency, and biological agreement with the measured hepatic phenotypes. HPD met the metabolite-connectivity, pathway-relevance, effect-size, and GSE156035 FDR criteria, whereas GSTM3 was retained on the basis of its GST-family/redox network relevance and agreement with oxidative-stress endpoints, not as a transcriptomically confirmed DEG.

### 4.10. Network Pharmacology-Assisted Compound–Target Analysis and Functional Enrichment

Representative constituents highlighted during the UHPLC-Orbitrap MS/MS chemical profiling were used as the chemical input set for the network pharmacology-assisted analysis. The constituent set was restricted to compounds that (i) were included among the putatively annotated constituents listed in Table S1, (ii) had literature-supported occurrence in one or more of the five SQJ botanical components, and (iii) were selected as recognizable chemical reference features on the basis of botanical-source attribution and marker relevance. These compounds were therefore intended to represent the experimentally profiled multicomponent SQJ matrix rather than to constitute a comprehensive list of all 250 annotations. Putative targets of these representative constituents were retrieved from the TCMSP database (https://tcmsp-e.com/, accessed on 6 February 2006) when available and standardized to official gene symbols using UniProt (https://www.uniprot.org/, accessed on 6 February 2006). Constituents for which no putative target information was retrievable did not contribute target entries to the downstream compound–target network. The selected compounds were not assumed to be confirmed absorbed constituents, direct pharmacodynamic compounds, or experimentally validated target ligands. Accordingly, the compound–target analysis was used as a hypothesis-generating prioritization layer rather than as evidence of direct target engagement.

The FDR-controlled transcriptomic analyses described in Section 4.9 were treated as the primary transcriptomic evidence layer and were kept separate by dataset. For transparency, the original exploratory network pharmacology analysis was retained as a supplementary hypothesis-generating analysis. This network was constructed by intersecting the SQJ-associated putative target set with the non-redundant union of genes meeting the original nominal exploratory threshold (raw *p* < 0.05 and |log2FC| > 0.585) in either GSE55098 or GSE156035, yielding 33 overlapping genes. Because this nominal-threshold union is permissive, the resulting network was not interpreted as FDR-supported or cross-dataset replicated transcriptomic evidence and was not used as the primary basis for HPD/GSTM3 prioritization.

The 33 overlapping genes were imported into STRING to construct a protein–protein interaction (PPI) network and were subsequently visualized in Cytoscape v3.8.2. The MCODE plug-in was used to identify densely connected subnetworks. Gene Ontology (GO) functional annotation and Kyoto Encyclopedia of Genes and Genomes (KEGG) pathway enrichment analyses were performed using DAVID, with *p* < 0.05 used as an exploratory enrichment threshold. Representative GO terms and KEGG pathways were selected for visualization based on nominal enrichment significance and biological relevance. Sankey–bubble diagrams were generated using CNSknowall (https://cnsknowall.com, accessed on 6 February 2006) to visualize relationships among the overlapping genes and representative enriched GO terms and KEGG pathways. These enrichment and visualization analyses were considered hypothesis-generating and were not interpreted as FDR-supported transcriptomic evidence or evidence of causal target engagement.

### 4.11. Gut Microbiota Analysis

Gut microbiota composition was analyzed using high-throughput sequencing of the bacterial 16S rRNA gene. Six eligible cecal-content samples per group were selected before sequencing according to the predefined sample order and sample availability, independently of microbial composition or downstream analytical outcomes. Total microbial DNA was extracted from the cecal contents using the cetyltrimethylammonium bromide method as previously described [82]. The V3–V4 hypervariable region of the bacterial 16S rRNA gene was amplified using the universal primers 341F (5′-CCTACGGGNGGCWGCAG-3′) and 805R (5′-GACTACHVGGGTATCTAATCC-3′). Sequencing libraries were prepared according to the manufacturer’s instructions, and paired-end sequencing was performed using an Illumina NovaSeq 6000 platform.

Raw paired-end reads were demultiplexed and subjected to quality filtering. The filtered reads were subsequently denoised, merged, and screened for chimeric sequences using the DADA2 workflow to generate amplicon sequence variants (ASVs). Representative ASV sequences were taxonomically annotated against the SILVA database, and relative abundance profiles were calculated at different taxonomic levels.

Microbial diversity analyses and statistical tests were conducted in R (v4.1.2). Beta diversity was evaluated using Bray–Curtis, Jaccard, unweighted UniFrac, and weighted UniFrac distance matrices and visualized using ordination analysis. Differences in overall microbial community composition among the five groups were formally evaluated using permutational multivariate analysis of variance (PERMANOVA) with 9,999 permutations. The results are reported as the proportion of variation explained by group membership (*R^*2*^*), the pseudo-(F) statistic, and the permutation-based (*p*)-value. Pairwise PERMANOVA was subsequently performed, and the resulting (*p*)-values were adjusted across the 10 pairwise group comparisons within each distance metric using the Benjamini–Hochberg procedure.

Differences among groups were evaluated using the Kruskal–Wallis test followed by Dunn’s post hoc test. Differential taxa were further identified using linear discriminant analysis effect size (LEfSe), with significance thresholds set at a Benjamini–Hochberg corrected *p* value < 0.05 and an LDA score > 4.0. PICRUSt2 was used to infer the potential functional profiles of the microbial communities from the 16S rRNA gene sequencing data. Differences in predicted pathway abundance between groups were evaluated using the Wilcoxon rank-sum test. The resulting PICRUSt2 profiles were interpreted as predictions of microbial functional potential.

### 4.12. SCFA Analysis

Short-chain fatty acids (SCFAs) in colonic contents were quantified using gas chromatography–mass spectrometry (GC-MS) using an internal-standard calibration approach, with slight modifications to a previously reported method [83]. Briefly, 100 mg of each colonic-content sample was mixed with 500 μL of ultrapure water, vortexed for 5 min, and centrifuged at 13,000 rpm for 5 min at 4 °C. A 200 μL aliquot of the supernatant was combined with 5 μL of 2-ethylbutyric acid internal-standard solution, 0.1 g NaCl, and 20 μL of 50% H_2_SO_4_. After vortexing for 1 min, the acidified mixture was extracted with 200 μL of methyl tert-butyl ether (MTBE), shaken for 1 min, and centrifuged at 13,000 rpm for 5 min at 4 °C. The upper organic phase was collected, and the aqueous phase was re-extracted with 100 μL of MTBE. The two organic phases were combined for GC-MS analysis.

Chromatographic separation was performed on an Agilent DB-WAX capillary column (30 m × 0.25 mm i.d., 0.25 μm film thickness). Helium (99.999%) was used as the carrier gas at a constant flow rate of 1.0 mL/min. The oven temperature was initially maintained at 70 °C, increased to 180 °C at 10 °C/min without a hold, and then increased to 200 °C at 5 °C/min. A 1 μL aliquot was injected using a 10:1 split ratio. The inlet, transfer-line, ion-source, and detector temperatures were 175, 280, 230, and 260 °C, respectively. The solvent delay was 3 min and the total run time was 15 min. Mass spectra were acquired under electron-impact ionization at 70 eV in full-scan mode over m/z 40–400. Because data were acquired and processed in full-scan mode rather than selected-ion monitoring mode, no predefined quantifier or qualifier ions were used. Each SCFA was identified by matching its retention time and full EI mass spectrum to those of the corresponding authentic standard, with spectral comparison performed in MassHunter v10.0.707.0. Acetic acid, propionic acid, isobutyric acid, butyric acid, isovaleric acid, and valeric acid were quantified from integrated total-ion chromatogram (TIC) peak areas relative to the TIC peak area of 2-ethylbutyric acid.

Calibration curves were prepared from mixed authentic-standard solutions serially diluted with MTBE. For each analyte, the ratio of analyte concentration to internal-standard concentration was used as the independent variable, and the ratio of analyte peak area to internal-standard peak area was used as the dependent variable. Method validation included linearity, precision, repeatability, and stability. Precision was assessed by six consecutive injections of a mixed SCFA standard solution at an intermediate concentration. Repeatability was evaluated using six independently prepared 100 mg aliquots of a pooled colonic-content quality-control sample. Stability was assessed by reanalyzing one prepared quality-control sample at 0, 1, 6, 12, 24, and 48 h. The corresponding calibration and validation results are presented in Tables S4 and S5. SCFA contents were calculated as (C × V)/M and expressed as μg/mg colonic content, where C is the concentration obtained from the calibration curve, V is the extraction volume, and M is the sample mass.

### 4.13. Hepatic Molecular and Oxidative-Stress Assessments

For targeted molecular follow-up, three animals per group were randomly selected before RT-qPCR analysis and four animals per group were randomly selected before Western blot analysis. Selection was performed independently of the resulting expression values. These assays were secondary, hypothesis-directed analyses of network-prioritized candidates and were therefore interpreted as supportive rather than as fully powered primary efficacy endpoints. RT-qPCR was performed to quantify the mRNA expression levels of HPD and GSTM3 in liver tissues. Total RNA was extracted using TRIzol reagent according to the manufacturer’s instructions. Gene-specific primers were synthesized by Sangon Biotech, and the primer sequences are listed in Table 1.

Reverse transcription was performed using 1 μg of total RNA with PrimeScript™ RT Master Mix. Quantitative PCR was conducted using TB Green™ Premix Ex Taq™ on a StepOnePlus™ Real-Time PCR System (Thermo Fisher Scientific, Waltham, MA, USA). Each 15 μL reaction contained 7.5 μL of TB Green Premix, 0.3 μL of each primer (10 μM), 1 μL of cDNA, and nuclease-free water. The cycling conditions were as follows: 95 °C for 30 s, followed by 40 cycles of 95 °C for 5 s and 60 °C for 30 s. No-template controls were included to monitor nonspecific amplification, and melting curve analysis was performed to verify primer specificity. All samples were analyzed in triplicate, with β-actin used as the internal reference gene. Relative mRNA expression was calculated using the 2^−ΔΔCt^ method. The triplicate reactions were technical replicates; the animal was the unit of biological replication and statistical analysis (*n* = 3).

Western blotting was performed to assess the protein expression levels of HPD and GSTM3 in liver tissues. Total protein was extracted using RIPA buffer supplemented with PMSF, and protein concentrations were determined using a BCA assay kit. Equal amounts of protein were separated by 8–15% SDS-PAGE and transferred onto PVDF membranes. After blocking with 5% skim milk in TBST, the membranes were incubated overnight at 4 °C with primary antibodies against HPD, GSTM3, and GAPDH. After washing with TBST, the membranes were incubated with HRP-conjugated secondary antibodies for 1 h at room temperature. Protein bands were visualized using an enhanced chemiluminescence system (Azure Biosystems C600), with exposure times adjusted to avoid signal saturation. Band intensities were quantified using ImageJ v1.8.0 and normalized to GAPDH. Each quantified lane represented liver tissue from one independent animal (*n* = 4).

Liver tissues stored at −80 °C were used for the determination of oxidative stress indicators. Briefly, liver tissues were accurately weighed, homogenized in pre-cooled buffer, and centrifuged to collect the supernatants. The levels of malondialdehyde (MDA), superoxide dismutase (SOD) activity, and glutathione (GSH) were determined using commercial assay kits according to the manufacturers’ instructions. The results were expressed relative to liver tissue weight as nmol/g tissue for MDA, U/g tissue for SOD, and μmol/g tissue for GSH.

### 4.14. Statistical Analysis

Statistical analyses were performed using GraphPad Prism 10.0. Normality was assessed using the Shapiro–Wilk test. Longitudinal fasting blood glucose and body-weight data were analyzed using two-way repeated-measures analysis of variance (ANOVA) with treatment group and time as factors, followed by Dunnett’s multiple-comparison test where appropriate. Single-endpoint normally distributed data, including OGTT-derived AUC and biochemical or molecular indicators, were analyzed using one-way ANOVA followed by Dunnett’s post hoc test. Non-normally distributed data were analyzed using the Kruskal–Wallis test followed by Dunn’s post hoc test. The metabolomics-specific univariate testing and false-discovery-rate correction procedures are described in Section 4.8. Unless otherwise specified, statistical significance was defined as *p* < 0.05. All *n* values represent independent biological replicates. The smaller histological, RT-qPCR, and Western blot subsets were secondary analyses; their *n* values were reported explicitly and their findings were interpreted as qualitative or supportive, as appropriate.

## 5. Conclusions

This study establishes SQJ as a chemically characterized multibotanical natural product extract and shows that its glucose-regulatory effects in STZ-induced diabetic mice are accompanied by distinct intestinal microbial, circulating metabolic, and hepatic redox responses. The divergent dose patterns of glycemic improvement and SCFA recovery, together with targeted assessment of HPD/GSTM3-associated hepatic responses and redox status, are consistent with a distributed microbial–host metabolic response rather than a single linear mechanism. These findings provide a systems pharmacology basis for further evaluation of SQJ in diabetic metabolic disturbance, while direct microbial mediation and the functional requirement for HPD/GSTM3 remain to be determined.

## Figures and Tables

**Table 1:** Primer sequences for RT-qPCR analysis of target genes in mice.

Gene	Forward Primer (5’-3’)	Reverse Primer (5’-3’)
*β-actin*	AGGTCATCACTATTGGCAACGAG	TTGGCATAGAGGTCTTTACGGAT
*HPD*	CATCAAAATGCCCATCAACGAG	AGAGCGATGTGCTGGACCCC
*GSTM3*	CTGACTCACTCCATCCGCTTGC	CGGTCAAAGTTGGGGGCATC

## Data Availability

The public transcriptomic datasets GSE55098 and GSE156035 are available from the NCBI Gene Expression Omnibus. Raw untargeted serum metabolomics data have been deposited in MetaboLights under accession number MTBLS15155 (https://www.ebi.ac.uk/metabolights/MTBLS15155, accessed on 6 October 2026). Raw 16S rRNA gene sequencing data from cecal content samples have been deposited in the NCBI Sequence Read Archive under BioProject accession number PRJNA1501892. Additional processed data supporting this article are included in the Supplementary Information.

## Supplementary Materials

The following supporting information can be downloaded at: https://www.mdpi.com/article/10.3390/ph19101602/s1. Table S1. Putative annotation of chemical constituents in the SQJ botanical extract by UHPLC-Orbitrap MS/MS; Table S2. Calibration curves and contents of representative marker constituents in the SQJ botanical extract; Table S3. Putatively annotated differential serum metabolites; Table S4. The linear parameters of each SCFA; Table S5. Precision, repeatability and stability of SCFAs (*n* = 6); Table S6. Overall and pairwise PERMANOVA results for gut microbial beta diversity; Table S7. Gene-level differential-expression summary, cross-dataset consistency analysis, and transcriptomic evidence for HPD and GSTM3; Figure S1. Total ion chromatograms of chemical constituents in the SQJ botanical extract; Figure S2. Representative chromatograms for quantitative determination of marker constituents in the SQJ botanical extract; Figure S3. Histopathological evaluation of the spleen, kidney, and duodenum in STZ-induced diabetic mice after SQJ botanical extract intervention; Figure S4. Total ion chromatograms of serum QC samples acquired in positive and negative ion modes; Figure S5. Network pharmacology-assisted compound–target analysis for prioritization of SQJ-associated host response nodes.

## Abbreviations

The following abbreviations are used in this manuscript:

ALT	Alanine aminotransferase
AR	Astragali Radix
AST	Aspartate aminotransferase
CF	Corni Fructus
CREG	Compound–reaction–enzyme–gene
DEG	Differentially expressed gene
DR	Dioscoreae Rhizoma
FBG	Fasting blood glucose
GEO	Gene Expression Omnibus
GSH	Glutathione
GSTM3	Glutathione S-transferase mu 3
HED	Human equivalent dose
HPD	4-Hydroxyphenylpyruvate dioxygenase
IL-4	Interleukin-4
IL-17	Interleukin-17
MDA	Malondialdehyde
OGTT	Oral glucose tolerance test
POR	Polygonati Odorati Rhizoma
PR	Polygonati Rhizoma
PUFA	Polyunsaturated fatty acid
SCFA	Short-chain fatty acid
SOD	Superoxide dismutase
SQJ	Shanqijing
SQJH	High-dose SQJ group
SQJL	Low-dose SQJ group
STZ	Streptozotocin
T1DM	Type 1 diabetes mellitus
TGF-β1	Transforming growth factor-β1
TNF-α	Tumor necrosis factor-α
