# Supplementary material for: Network Pharmacology-Assisted Multi-Omics Analysis of Gut Microbiota–Host Metabolic Remodeling and Hepatic HPD/GSTM3-Associated Redox Responses Following Shanqijing Treatment in STZ-Induced Diabetic Mice
Source: Pharmaceuticals (Basel). 2026 Oct 9;19(10):1602. doi: 10.3390/ph19101602 (PMC13708638; doi:10.3390/ph19101602)
Supplement: Supplementary file 1 [file pharmaceuticals-19-01602-s001.zip › pharmaceuticals-4517687-supplementary.pdf]

Supplementary Tables

Table S1. Putative annotation of chemical constituents in SQJ botanical extract by UHPLC-Orbitrap MS/MS.

No.	Chemical Name	Formula	RT/min	Experimental m/z	Calculated m/z	Error (ppm)	Precursor/adduct ion	Diagnostic MS/MS fragments	Reported botanical source	Annotation level	references
1	Glutaric acid	$C_5H_8O_4$	0.57	131.0351	131.0350	0.76	$[M-H]^-$	131.0348, 113.0247, 87.0451, 85.0295, 73.0294, 71.0138, 57.0346	DR	MSI Level 2	[1]
2	7,4-Dihydroxyflavone	$C_{15}H_{10}O_4$	0.58	255.0656	255.0657	-0.39	$[M+H]^+$	255.0656, 227.0703, 181.0648, 153.0703, 152.0626, 121.0287	DR	MSI Level 2	[1]
3	4-Methylcatechol	$C_7H_8O_2$	0.69	125.0601	125.0602	-0.80	$[M+H]^+$	111.0236, 97.0286, 79.0180, 69.0337, 67.0545	DR	MSI Level 2	[1]
4	Niacin acid	$C_6H_5NO_2$	0.92	122.0249	122.0248	0.82	$[M-H]^-$	122.0247, 94.0298, 78.0351	AR	MSI Level 2	[2]
5	Vanillin	$C_8H_8O_3$	1.09	151.0402	151.0401	0.66	$[M-H]^-$	151.0398, 136.0145, 123.0452, 107.0502, 71.0139	POR	MSI Level 2	[3]
6	Spermidine	$C_7H_{19}N_3$	1.11	146.1653	146.1652	0.68	$[M+H]^+$	146.0603, 129.1388, 112.1123, 84.0809, 72.0809, 58.0654	DR	MSI Level 2	[1]
7	Canavanine	$C_5H_{12}N_4O_3$	1.18	175.0840	175.0837	1.71	$[M-H]^-$	175.0835, 146.9611, 118.0509, 98.0247, 74.0359	AR	MSI Level 2	[2]
8	Lysine	$C_6H_{14}N_2O_2$	1.22	147.1130	147.1128	1.36	$[M+H]^+$	147.0567, 130.0865, 119.0499, 105.0041, 84.0809, 68.9823	PR, DR	MSI Level 2	[1]
9	Histidine	$C_6H_9N_3O_2$	1.26	154.0625	154.0622	1.95	$[M-H]^-$	154.0621, 137.0356, 136.0516, 110.0723, 93.0457	AR, PR, DR	MSI Level 2	[1]
10	Ornithine	$C_5H_{12}N_2O_2$	1.28	131.0827	131.0826	0.76	$[M-H]^-$	131.0825, 114.0196, 113.0356, 95.0250, 70.0298	DR	MSI Level 2	[1]
11	Aspartic acid	$C_4H_7NO_4$	1.31	132.0303	132.0302	0.76	$[M-H]^-$	132.0302, 115.0036, 88.0404, 71.0139	AR, DR	MSI Level 2	[1]
12	Arginine	$C_6H_{14}N_4O_2$	1.31	175.1190	175.1190	0.00	$[M+H]^+$	175.1192, 158.0928, 130.0978, 116.0708, 70.0653, 60.0559	AR, PR, DR	MSI Level 2	[1]
13	Glutamine	$C_5H_{10}N_2O_3$	1.31	145.0620	145.0619	0.69	$[M-H]^-$	145.0619, 128.0352, 127.0513, 125.0357, 109.0406, 84.0454, 74.0248, 58.0297	PR	MSI Level 2	[4]
14	Asparagine	$C_4H_8N_2O_3$	1.32	133.0609	133.0608	0.75	$[M+H]^+$	133.0611, 116.0344, 87.0555, 74.0238	DR	MSI Level 2	[1]
15	Mannitol	$C_6H_{14}O_6$	1.32	181.0720	181.0718	1.10	$[M-H]^-$	181.0717, 163.0614, 119.0348, 101.0243, 96.9599, 89.0244, 85.0294, 71.0138, 59.0138	POR	MSI Level 2	[3]
16	Fructose-6-phosphate	$C_6H_{13}O_9P$	1.35	259.0226	259.0224	0.77	$[M-H]^-$	259.0150, 241.0082, 168.9796, 138.9809, 96.9694, 78.9590	PR	MSI Level 2	[4]
17	Tartaric acid	$C_4H_6O_6$	1.35	149.0091	149.0092	0.00	$[M-H]^-$	149.009, 130.9984, 103.0036, 87.0087, 72.9931, 59.0139	CF	MSI Level 2	[5]

No.	Chemical Name	Formula	RT/min	Experimental m/z	Calculated m/z	Error (ppm)	Precursor/adduct ion	Diagnostic MS/MS fragments	Reported botanical source	Annotation level	references
18	Homoserine	$C_4H_9NO_3$	1.36	118.0510	118.0510	0.00	$[M-H]^-$	118.0510, 98.0247, 72.0454, 55.0188	DR	MSI Level 2	[1]
19	Glutamic acid	$C_5H_9NO_4$	1.36	146.0459	146.0459	0.00	$[M-H]^-$	146.0458, 128.0352, 102.0560	AR, DR, POR	MSI Level 2	[3]
20	D-Fructose	$C_6H_{12}O_6$	1.36	179.0562	179.0561	0.56	$[M-H]^-$	179.0561, 161.0654, 113.0243, 101.0243, 89.0244, 71.0138, 59.0138	PR, DR, CF	MSI Level 2	[1]
21	Threonine	$C_4H_9NO_3$	1.36	120.0657	120.0655	1.67	$[M+H]^+$	120.0656, 102.0552, 84.0446, 74.0601, 56.0498	AR, PR	MSI Level 2	[4]
22*	Allantoin	$C_4H_6N_4O_3$	1.40	157.0366	157.0367	-0.64	$[M-H]^-$	96.9601, 59.0138, 55.0566	AR, DR	MSI Level 2	[1]
23	Quinic acid	$C_7H_{12}O_6$	1.40	191.0563	191.0561	1.05	$[M-H]^-$	191.0560, 127.0400, 93.0345, 85.0295, 57.0345	DR	MSI Level 2	[1]
24	Trigonelline	$C_7H_7NO_2$	1.42	138.0551	138.0550	0.72	$[M+H]^+$	138.0551, 110.0602, 94.0653, 92.0496, 78.0339, 65.0388	DR	MSI Level 2	[1]
25	N-Acetylproline	$C_7H_{11}NO_3$	1.46	158.0812	158.0812	0.00	$[M+H]^+$	158.0814, 114.0552, 112.0759, 86.0602, 70.0654, 68.0496	CF	MSI Level 2	[5]
26	Maltotetraose	$C_{24}H_{42}O_{21}$	1.46	665.2152	665.2146	0.90	$[M-H]^-$	665.2150, 545.1732, 485.1514, 383.1197, 341.1090, 323.0969, 221.0668, 179.0561, 113.0244, 89.0244	POR	MSI Level 2	[3]
27	Lactose	$C_{12}H_{22}O_{11}$	1.48	341.1089	341.1089	0.00	$[M-H]^-$	341.1091, 179.0561, 161.0456, 143.0350, 119.0349, 113.0244, 101.0244, 89.0244, 71.0138, 59.0138	POR	MSI Level 2	[3]
28	Sucrose	$C_{12}H_{22}O_{11}$	1.48	341.1089	341.1089	0.00	$[M-H]^-$	341.1090, 179.0561, 161.0455, 119.0349, 113.0243, 101.0244, 89.0244, 71.0138, 59.0138	AR, CF	MSI Level 2	[2]
29	Fumaric acid	$C_4H_4O_4$	1.51	115.0036	115.0037	-0.87	$[M-H]^-$	115.0035, 97.6751, 71.0138	DR	MSI Level 2	[1]
30	Adenosine 5'-monophosphate	$C_{10}H_{14}N_5O_7P$	1.53	348.0709	348.0704	1.44	$[M+H]^+$	348.1014, 136.0620, 119.0359, 97.0286, 69.0332	PR	MSI Level 2	[4]
31	D-glucosamine	$C_6H_{13}NO_5$	1.56	180.0868	180.0866	1.11	$[M+H]^+$	180.0653, 152.0705, 145.0496, 134.0604, 127.0392, 85.0286, 72.0445, 69.0336	PR	MSI Level 2	[4]
32	Raffinose	$C_{18}H_{32}O_{16}$	1.58	503.1619	503.1618	0.20	$[M-H]^-$	503.1621, 323.0979, 221.0667, 179.0561, 161.0455, 89.0244	POR	MSI Level 2	[3]
33	Adenine	$C_5H_5N_5$	1.61	136.0619	136.0618	0.73	$[M+H]^+$	136.0620, 127.9205, 119.0355, 108.0809, 94.0401, 91.0545	AR, DR, POR	MSI Level 2	[3]

No.	Chemical Name	Formula	RT/min	Experimental m/z	Calculated m/z	Error (ppm)	Precursor/adduct ion	Diagnostic MS/MS fragments	Reported botanical source	Annotation level	references
34	Isomaltotetraose	$C_{24}H_{42}O_{21}$	1.64	665.2153	665.2146	1.05	$[M-H]^-$	665.2155, 485.1523, 383.1191, 179.0561, 113.0244, 89.0244, 59.0138	POR	MSI Level 2	[3]
35	Malic acid	$C_4H_6O_5$	1.66	133.0140	133.0142	-1.50	$[M-H]^-$	133.0142, 115.0036, 89.0244, 72.9930, 71.0138, 62.3429	PR, DR, CF	MSI Level 2	[4]
36	Maleic acid	$C_4H_4O_4$	1.68	115.0037	115.0037	0.00	$[M-H]^-$	115.0035, 97.4921, 89.9393, 71.0138, 68.9958, 54.7278	POR	MSI Level 2	[3]
37	2'-Deoxyguanosine 5'-phosphate	$C_{10}H_{14}N_5O_7P$	1.71	348.0709	348.0709	0.00	$[M+H]^+$	259.4858, 219.1128, 136.0620, 97.0286, 55.5699	DR	MSI Level 2	[1]
38	4-guanidinobutanoate	$C_5H_{11}N_3O_2$	1.77	146.0925	146.0924	0.68	$[M+H]^+$	N.D.	DR	MSI Level 3 (tentative)	[1]
39	1-Methyladenosine	$C_{11}H_{15}N_5O_4$	1.89	282.1187	282.1197	-3.54	$[M+H]^+$	264.1338, 246.1235, 218.1280, 150.0778, 85.0284, 84.0809, 72.0809, 57.0337, 55.0180	DR	MSI Level 2	[1]
40	Proline	$C_5H_9NO_2$	1.95	114.0559	114.0561	-1.75	$[M-H]^-$	114.0559, 86.0328, 71.0138, 68.9956	AR, DR, POR	MSI Level 2	[3]
41	Citric Acid	$C_6H_8O_7$	1.96	191.0199	191.0197	1.05	$[M-H]^-$	191.0560, 173.0455, 127.0400, 111.0087, 87.0087, 85.0295, 57.0344	DR, CF, POR	MSI Level 2	[3]
42	Stachyose	$C_{24}H_{42}O_{21}$	1.99	665.2159	665.2146	1.95	$[M-H]^-$	665.2151, 503.1620, 454.7924, 383.1194, 301.9842, 179.0556, 89.0244	POR	MSI Level 2	[3]
43	Lotaustralin	$C_{11}H_{19}NO_6$	2.02	262.1287	262.1285	0.76	$[M+H]^+$	262.1292, 244.1183, 216.1234, 198.1131, 161.0685, 143.0581, 130.0866, 118.0865, 112.0396, 97.0287, 84.0810, 72.0810	PR	MSI Level 2	[4]
44	trans-Aconitic acid	$C_6H_6O_6$	2.11	173.0092	173.0092	0.00	$[M-H]^-$	173.0094, 129.0193, 111.0087, 85.0295	CF	MSI Level 2	[5]
45	Aconitic Acid	$C_6H_6O_6$	2.11	173.0092	173.0092	0.00	$[M-H]^-$	173.0094, 145.0408, 129.0193, 111.0087, 85.0295, 67.0190	DR	MSI Level 2	[1]
46	Pyroglutamic acid	$C_5H_7NO_3$	2.23	130.0501	130.0499	1.54	$[M+H]^+$	130.0501, 84.0445, 56.0498	AR, PR, DR	MSI Level 2	[1]
47	Cyclic GMP	$C_{10}H_{12}N_5O_7P$	2.30	344.0403	344.0402	0.29	$[M-H]^-$	344.0317, 301.9812, 207.6742, 150.0420, 133.0155, 108.0206, 77.4139, 52.3497	AR	MSI Level 2	[2]
48*	Uridine	$C_9H_{12}N_2O_6$	2.35	243.0624	243.0623	0.41	$[M-H]^-$	243.0622, 200.0566, 152.0351, 140.0354, 122.0247, 110.0247, 96.0456, 82.0299, 66.0349	AR, DR, POR	MSI Level 2	[3]

No.	Chemical Name	Formula	RT/min	Experimental m/z	Calculated m/z	Error (ppm)	Precursor/adduct ion	Diagnostic MS/MS fragments	Reported botanical source	Annotation level	references
49	5-Aminolevulinic acid	$C_5H_9NO_3$	2.36	130.0509	130.0510	-0.77	$[M-H]^-$	130.0511, 112.0404, 88.0404	DR	MSI Level 2	[1]
50*	Arbutin	$C_{12}H_{16}O_7$	2.47	271.0827	271.0823	1.48	$[M-H]^-$	211.0252, 169.0142, 125.0244, 108.0216, 71.0137	DR	MSI Level 2	[1]
51	Tyrosine	$C_9H_{11}NO_3$	2.59	182.0813	182.0812	0.55	$[M+H]^+$	182.0807, 165.0549, 136.0759, 123.0442, 119.0494, 91.0544	AR, DR, POR	MSI Level 2	[3]
52	beta-Glucogallin	$C_{13}H_{16}O_{10}$	2.61	331.0672	331.0671	0.30	$[M-H]^-$	331.0671, 271.0462, 211.0249, 169.0143, 168.0065, 151.0035, 125.0243, 123.0087, 89.0243, 59.0138	DR	MSI Level 2	[1]
53	Glucogallin	$C_{13}H_{16}O_{10}$	2.61	331.0672	331.0671	0.30	$[M-H]^-$	331.0671, 271.0462, 211.0249, 169.0143, 168.0065, 125.0243, 89.0243, 59.0138	DR, CF	MSI Level 2	[1]
54	Dopamine	$C_8H_{11}NO_2$	2.62	136.0759	136.0757	1.47	$[M+H-H2O]^+$	119.0494, 91.0544, 65.0391, 109.0649	DR	MSI Level 2	[1]
55	p-Coumaric acid	$C_9H_8O_3$	2.62	165.0549	165.0552	-1.82	$[M+H]^+$	147.0443, 123.0442, 119.0494, 95.0493, 91.0544, 77.0387	PR, DR	MSI Level 2	[1]
56	2-Methylmalic acid	$C_5H_8O_5$	2.66	147.0299	147.0299	0.00	$[M-H]^-$	147.0288, 129.0186, 115.0036, 103.0401, 87.0087, 71.0138	CF	MSI Level 2	[5]
57	2'-Deoxyguanosine 5'-monophosphate	$C_{10}H_{14}N_5O_7P$	2.67	346.0561	346.0558	0.87	$[M-H]^-$	211.0016, 134.0472, 96.9695, 78.9590	DR	MSI Level 2	[1]
58*	5-Hydroxymaltol	$C_6H_6O_4$	2.80	143.0340	143.0339	0.70	$[M+H]^+$	143.0341, 125.0235, 113.0235, 97.0286, 83.0128, 69.0337, 55.0182	PR	MSI Level 2	[6]
59*	Adenosine	$C_{10}H_{13}N_5O_4$	2.91	268.1042	268.1046	-1.49	$[M+H]^+$	268.1042, 207.6695, 136.0620, 119.0355, 85.0285, 72.5094, 57.0338	AR, PR, DR, POR	MSI Level 2	[3]
60*	Gallic acid	$C_7H_6O_5$	3.19	169.0143	169.0142	0.59	$[M-H]^-$	169.0143, 125.0243, 107.0138, 97.0295, 79.0190	CF	MSI Level 2	[5]
61	Shikimic acid	$C_7H_{10}O_5$	3.30	173.0456	173.0455	0.58	$[M-H]^-$	173.0462, 155.0349, 129.0556, 111.0451, 93.0345, 85.0295, 83.0502, 57.0345	DR	MSI Level 2	[1]
62	Leucine	$C_6H_{13}NO_2$	3.34	132.1021	132.1019	1.51	$[M+H]^+$	132.1026, 104.0577, 86.0966, 69.0700	AR, DR, POR	MSI Level 2	[3]
63	Isoleucine	$C_6H_{13}NO_2$	3.34	132.1021	132.1019	1.51	$[M+H]^+$	104.0577, 86.0966, 69.0700, 55.9308	AR, DR	MSI Level 2	[1]
64*	Guanosine	$C_{10}H_{13}N_5O_5$	3.34	284.0993	284.0989	1.41	$[M+H]^+$	284.0994, 152.0569, 135.0303, 110.0352	AR, DR,	MSI Level 2	[3]

No.	Chemical Name	Formula	RT/min	Experimental m/z	Calculated m/z	Error (ppm)	Precursor/adduct ion	Diagnostic MS/MS fragments	Reported botanical source	Annotation level	references
									POR		
65	Isoguanosine	$C_{10}H_{13}N_5O_5$	3.51	284.0991	284.0989	0.70	$[M+H]^+$	152.0569, 135.0303, 110.0351	POR	MSI Level 2	[3]
66	2'-Deoxyguanosine	$C_{10}H_{13}N_5O_4$	3.94	266.0895	266.0895	0.00	$[M-H]^-$	266.0904, 234.0092, 176.0573, 150.0421, 133.0152, 102.2603	AR	MSI Level 2	[2]
67	7-O-Galloyl-D-sedoheptulose	$C_{14}H_{18}O_{11}$	4.02	361.0775	361.0776	-0.28	$[M-H]^-$	361.0782, 301.0578, 271.0461, 211.0249, 169.0143, 125.0243, 59.0139	CF	MSI Level 2	[5]
68*	5-(Hydroxymethyl)furfural (5-HMF)	$C_6H_6O_3$	4.94	127.0392	127.0390	1.57	$[M+H]^+$	127.0504, 110.0238, 109.0286, 99.0440, 84.0445, 81.0336, 69.0337, 55.0182, 53.0389	PR, CF, POR	MSI Level 2	[3]
69	Phenylalanine	$C_9H_{11}NO_2$	4.96	166.0865	166.0863	1.20	$[M+H]^+$	166.0863, 149.0598, 131.0493, 120.0809, 103.0544, 93.0700, 79.0542	AR, PR, DR, CF	MSI Level 2	[1]
70	5-Formyltetrahydrofolate	$C_{20}H_{23}N_7O_7$	5.05	474.1742	474.1737	1.05	$[M+H]^+$	474.1749, 345.1310, 327.1203, 132.0448	DR	MSI Level 2	[1]
71*	Protocatechuic acid	$C_7H_6O_4$	5.06	153.0194	153.0193	0.65	$[M-H]^-$	153.0193, 109.0294, 81.0345	DR, CF	MSI Level 2	[1]
72	Vanillic acid	$C_8H_8O_4$	5.06	167.0352	167.0350	1.20	$[M-H]^-$	167.0349, 152.0113, 137.0243, 123.0451, 108.0216, 93.0345, 65.0396	AR, PR, DR	MSI Level 2	[2]
73	D-Pantothenic acid	$C_9H_{17}NO_5$	5.07	218.1036	218.1034	0.92	$[M-H]^-$	218.1035, 146.0822, 88.0404, 71.0502	DR	MSI Level 2	[1]
74	Succinyl Adenosine	$C_{14}H_{17}N_5O_8$	5.07	384.1158	384.1150	2.08	$[M+H]^+$	384.1148, 252.0729, 234.0623, 206.0674, 192.0518, 188.0568, 162.0777, 148.0618, 136.0620	AR	MSI Level 2	[2]
75	Secologanin	$C_{17}H_{24}O_{10}$	5.11	389.1450	389.1442	2.06	$[M+H]^+$	389.1711, 357.0686, 247.1804, 227.0917, 209.0811, 195.0655, 139.0391	CF	MSI Level 2	[5]
76	1,2,3-Tri-O-galloyl-β-D-glucose	$C_{27}H_{24}O_{18}$	5.13	635.0890	635.0890	0.00	$[M-H]^-$	635.0880, 465.0679, 313.0566, 169.0143, 125.0243	CF	MSI Level 2	[5]
77	Indole-3-carboxaldehyde	C_9H_7NO	5.16	146.0602	146.0600	1.37	$[M+H]^+$	146.0603, 128.0500, 118.0654, 117.0575, 110.0602, 100.0759, 91.0544, 86.0966, 81.0336, 53.1743	AR, PR, DR	MSI Level 2	[1]
78	Eriobioside	$C_{23}H_{36}O_{16}$	5.17	613.1998	613.1974	3.91	$[M+HCOO]^-$	567.1979, 405.1396, 285.0978, 243.0872, 155.0349, 101.0243	CF	MSI Level 2	[5]
79	1-O,7-O-Digalloyl-D-sedoheptulose	$C_{21}H_{22}O_{15}$	5.19	513.0882	513.0886	-0.78	$[M-H]^-$	513.0886, 423.0555, 361.0776, 271.0461, 211.0450, 169.0143, 125.0243	CF	MSI Level 2	[5]
80	5-Methylthioadenosine	$C_{11}H_{15}N_5O_3S$	5.20	298.0974	298.0974	0.00	$[M+H]^+$	234.1124, 193.1059, 163.0428, 136.0620, 61.0109	DR	MSI Level 2	[1]
81	5-Deoxy-5-methylthioadenosine	$C_{11}H_{15}N_5O_3S$	5.20	298.0974	298.0968	2.01	$[M+H]^+$	298.0968, 136.0620, 97.0285, 75.0264, 61.0109	PR	MSI Level 2	[4]

No.	Chemical Name	Formula	RT/min	Experimental m/z	Calculated m/z	Error (ppm)	Precursor/adduct ion	Diagnostic MS/MS fragments	Reported botanical source	Annotation level	references
82	2,3-Di-O-Galloyl-D-glucose	$C_{20}H_{20}O_{14}$	5.21	483.0788	483.0780	1.66	$[M-H]^-$	483.1102, 439.0881, 331.0672, 313.0565, 287.0775, 271.0460, 169.0143, 125.0242	CF	MSI Level 2	[5]
83	Tryptophan	$C_{11}H_{12}N_2O_2$	5.24	205.0974	205.0972	0.98	$[M+H]^+$	205.1338, 188.0708, 146.0602, 118.0653, 85.0286, 58.0654	AR, PR, DR	MSI Level 2	[1]
84	trans-3-Indoleacrylic acid	$C_{11}H_9NO_2$	5.24	188.0708	188.0706	1.06	$[M+H]^+$	170.0607, 146.0602, 144.0810, 143.0732, 142.0655, 118.0653, 117.0700, 115.0545, 91.0545	PR	MSI Level 2	[4]
85	Chlorogenic acid	$C_{16}H_{18}O_9$	5.25	353.0881	353.0878	0.85	$[M-H]^-$	353.0894, 285.0614, 191.0561, 179.0352, 173.0455, 135.0453, 85.0295	AR	MSI Level 2	[2]
86	Polygonatine A	$C_9H_{11}NO_2$	5.27	166.0865	166.0863	1.20	$[M+H]^+$	166.0863, 136.0759, 120.0810, 80.0495	PR	MSI Level 2	[6]
87	Cinnamic acid	$C_9H_8O_2$	5.28	149.0599	149.0602	-2.01	$[M+H]^+$	149.0598, 121.0650, 103.0544, 91.0544, 77.0387, 53.0390	PR, DR	MSI Level 2	[1]
88	4-Methoxycoumarine	$C_{10}H_8O_3$	5.29	177.0543	177.0546	-1.69	$[M+H]^+$	177.0547, 149.0598, 133.0650, 131.0494, 121.0650, 109.0286, 105.0710	AR	MSI Level 2	[2]
89*	Cornin	$C_{17}H_{24}O_{10}$	5.29	389.1447	389.1442	1.28	$[M+H]^+$	357.1226, 285.0766, 227.0914, 209.0803, 195.0655, 181.0860, 177.0546, 149.0600, 139.0391	CF, POR	MSI Level 2	[5]
90	Paeonol	$C_9H_{10}O_3$	5.30	167.0705	167.0703	1.20	$[M+H]^+$	167.0706, 149.0599, 135.0807, 121.0287, 91.0544, 77.0387, 65.0387	PR	MSI Level 2	[4]
91	Loganic acid	$C_{16}H_{24}O_{10}$	5.31	375.1300	375.1297	0.80	$[M-H]^-$	375.1297, 213.0770, 169.0872, 151.0763, 113.0243, 69.0345, 59.0138	CF	MSI Level 2	[5]
92*	Morroniside	$C_{17}H_{26}O_{11}$	5.31	405.1411	405.1402	2.22	$[M-H]^-$	405.1384, 243.0873, 155.0349, 141.0557, 101.0244, 89.0244, 71.0138, 68.9982, 59.0138	CF	MSI Level 2	[5]
93	Apigenin-7-glucoside	$C_{21}H_{20}O_{10}$	5.31	433.1136	433.1134	0.46	$[M+H]^+$	271.0602, 187.0604, 127.0391	DR	MSI Level 2	[1]
94	Dihydrocaffeic acid	$C_9H_{10}O_4$	5.31	183.0653	183.0652	0.55	$[M+H]^+$	183.0658, 165.0548, 155.0340, 141.0548, 123.0442, 111.0443, 109.0285, 95.0493, 91.0544, 81.0336, 79.0538	PR	MSI Level 2	[6]
95	Secologanoside	$C_{16}H_{22}O_{11}$	5.33	389.1096	389.1089	1.80	$[M-H]^-$	389.1072, 345.1194, 227.0924, 165.0558, 121.0658, 101.0243, 69.0346	CF	MSI Level 2	[5]
96	Cafeoyltartaric acid dimethyl ester	$C_{15}H_{16}O_9$	5.33	339.0722	339.0722	0.00	$[M-H]^-$	339.0724, 227.0730, 179.0350, 161.0244, 135.0452,	CF	MSI Level 2	[5]
97	Tellimagrandin I	$C_{34}H_{26}O_{22}$	5.34	785.0865	785.0843	2.80	$[M-H]^-$	785.0854, 506.1564, 483.0776, 419.0632, 300.9989, 275.0200, 249.0401, 169.0142, 125.0243	CF	MSI Level 2	[5]
98	Coumarin	$C_9H_6O_2$	5.36	147.0442	147.0441	0.68	$[M+H]^+$	147.0443, 119.0494, 91.0544, 65.0389	AR	MSI Level 2	[2]
99	6-C-beta-xylopyranosyl-8-C-beta-galactopyrano-side	$C_{26}H_{28}O_{14}$	5.37	563.1407	563.1400	1.24	$[M-H]^-$	563.1392, 443.0993, 383.0771, 353.0669, 227.0925, 173.0456, 127.0400, 111.0087	DR	MSI Level 2	[1]
100	7-Dehydrologanin	$C_{17}H_{24}O_{10}$	5.37	389.1434	389.1442	-2.06	$[M+H]^+$	389.1410, 327.1418, 227.0913, 195.0652, 139.0392, 86.0966	CF	MSI Level 2	[5]

No.	Chemical Name	Formula	RT/min	Experimental m/z	Calculated m/z	Error (ppm)	Precursor/adduct ion	Diagnostic MS/MS fragments	Reported botanical source	Annotation level	references
101	5-(9H-β-carbolin-1-yl)-pentane -1,2,5-triol	$C_{16}H_{18}N_2O_3$	5.38	287.1395	287.1390	1.74	$[M+H]^+$	287.1719, 287.1388, 269.1290, 251.1185, 209.1080, 195.1608, 182.1542, 167.0706, 158.1292, 107.0493, 60.0559	PR	MSI Level 2	[4]
102	Caftaric acid	$C_{13}H_{12}O_9$	5.39	311.0413	311.0409	1.29	$[M-H]^-$	311.0412, 179.0349, 149.0091, 135.0452, 87.0087, 59.0137	CF	MSI Level 2	[5]
103	3-O-(E)-Feruloylquinic acid	$C_{17}H_{20}O_9$	5.39	367.1038	367.1035	0.82	$[M-H]^-$	367.1600, 299.0770, 193.0504, 191.0561, 173.0455, 137.0244, 134.0372, 93.0345	DR	MSI Level 2	[1]
104	Pratensein	$C_{16}H_{12}O_6$	5.39	301.0711	301.0707	1.33	$[M+H]^+$	301.0699, 283.1067, 269.0448, 241.0498, 213.0548, 137.0236	PR	MSI Level 2	[6]
105	Swertiamarine	$C_{16}H_{22}O_{10}$	5.39	433.1352	433.1341	2.54	$[M+CH3COO]^-$	433.1071, 365.1447, 271.0613, 225.0768, 101.0242	CF	MSI Level 2	[5]
106	7-β-O-dimethyl butanedioate morroniside	$C_{23}H_{34}O_{15}$	5.39	609.2046	609.2045	0.16	$[M+CH3COO]^-$	609.0964, 389.1440, 301.0355, 227.0925, 173.0456, 127.0400, 101.0243	CF	MSI Level 2	[5]
107*	Syringic acid	$C_9H_{10}O_5$	5.40	197.0458	197.0455	1.52	$[M-H]^-$	197.0456, 182.0220, 166.9986, 153.0556, 138.0322, 123.0087, 121.0294, 106.0059	DR	MSI Level 2	[1]
108	4-Methylumbelliferyl mannoside	$C_{16}H_{18}O_8$	5.40	337.0935	337.0929	1.78	$[M-H]^-$	337.0935, 191.0561, 173.0457, 163.0402, 119.0501, 93.0345	DR	MSI Level 2	[1]
109	Ferulaldehyde	$C_{10}H_{10}O_3$	5.40	179.0705	179.0703	1.12	$[M+H]^+$	179.0705, 161.0599, 151.0756, 147.0443, 133.0651, 119.0495, 109.0650, 105.0702, 91.0544, 81.0700	POR	MSI Level 2	[3]
110	7-O-Methylmorroniside	$C_{18}H_{28}O_{11}$	5.4	419.1567	419.1559	1.91	$[M-H]^-$	419.1032, 389.1617, 373.1513, 257.0822, 211.0974, 181.0505, 151.0403, 101.0243, 59.0138	CF	MSI Level 2	[5]
111	Esculetin	$C_9H_6O_4$	5.42	177.0194	177.0193	0.56	$[M-H]^-$	162.0322, 133.0295, 129.0192, 99.0087, 71.0138	DR	MSI Level 2	[1]
112	Sarracenin	$C_{11}H_{14}O_5$	5.42	225.0767	225.0768	-0.44	$[M-H]^-$	225.0782, 179.0561, 123.0451, 101.0243, 89.0244, 68.9982	CF	MSI Level 2	[5]
113	2,3,4,6-tetrahydro-1H-β-carboline-3-carboxylic acid	$C_{12}H_{12}N_2O_2$	5.42	217.0975	217.0972	1.38	$[M+H]^+$	217.0986, 171.0922, 144.0810, 143.0730, 74.0238	PR	MSI Level 2	[4]
114	6-Methylcoumarin	$C_{10}H_8O_2$	5.42	161.0599	161.0597	1.24	$[M+H]^+$	161.0599, 133.0650, 115.0545, 105.0701, 103.0544, 91.0544	AR	MSI Level 2	[2]
115	Cornuside III	$C_{21}H_{30}O_{14}$	5.42	505.1562	505.1563	-0.20	$[M-H]^-$	505.1568, 389.1467, 227.0925, 127.0400, 101.0243, 71.0136	CF	MSI Level 2	[5]
116*	Sweroside	$C_{16}H_{22}O_9$	5.43	381.1164	381.1156	2.10	$[M+Na]^+$	359.1338, 197.0811, 179.0704, 151.0756, 137.0603, 127.0392, 111.0807, 86.0966	CF	MSI Level 2	[5]
117*	Quercetin	$C_{15}H_{10}O_7$	5.43	303.0502	303.0499	0.99	$[M+H]^+$	303.0502, 285.0399, 274.0468, 257.0447, 247.0605, 229.0496, 201.0548, 165.0185, 153.0184, 137.0235, 121.0286	AR, PR, DR, CF	MSI Level 2	[1]

No.	Chemical Name	Formula	RT/min	Experimental m/z	Calculated m/z	Error (ppm)	Precursor/adduct ion	Diagnostic MS/MS fragments	Reported botanical source	Annotation level	references
118	Texasin	$C_{16}H_{12}O_5$	5.44	283.0617	283.0612	1.77	$[M-H]^-$	283.0612, 268.0379, 239.0352, 211.0403, 184.0529, 148.0164	CF	MSI Level 2	[5]
119	Caffeate	$C_9H_8O_4$	5.46	179.0351	179.0350	0.56	$[M-H]^-$	135.0451, 107.0503, 89.0244, 71.0138, 59.0138	AR, DR	MSI Level 2	[1]
120	Oroxylin A-7-O-glucoside	$C_{22}H_{22}O_{10}$	5.46	447.1293	447.1286	1.57	$[M+H]^+$	447.1255, 285.0758, 270.0524, 253.0499, 225.0547, 137.0235, 71.2703	AR	MSI Level 2	[2]
121	2-Isopropylmalic acid	$C_7H_{12}O_5$	5.46	175.0613	175.0612	0.57	$[M-H]^-$	175.0612, 157.0507, 131.0713, 115.0400, 113.0608, 85.0659	DR	MSI Level 2	[1]
122*	Calycosin-7-glucoside	$C_{22}H_{22}O_{10}$	5.46	447.1293	447.1291	0.45	$[M+H]^+$	447.1255, 285.0758, 270.0524, 253.0499, 225.0547, 137.0235	AR	MSI Level 2	[2]
123	Loganetin	$C_{11}H_{16}O_5$	5.47	227.0928	227.0925	1.32	$[M-H]^-$	227.0926, 127.0400, 101.0244, 68.9982, 67.0180	CF	MSI Level 2	[5]
124*	Loganin	$C_{17}H_{26}O_{10}$	5.47	435.1511	435.1497	3.22	$[M+HCOO]^-$	435.1508, 227.0924, 207.6797, 127.0399, 101.0246, 94.5131, 67.0189	CF	MSI Level 2	[5]
125	N-Feruloyloctopamine	$C_{18}H_{19}NO_5$	5.48	328.1197	328.1190	2.13	$[M-H]^-$	328.1181, 310.1085, 295.0854, 252.0792, 175.0405, 161.0245, 149.0610, 134.0373, 133.0533, 85.0295	PR, POR	MSI Level 2	[3]
126	Schaftoside	$C_{26}H_{28}O_{14}$	5.48	565.1554	565.1557	-0.53	$[M+H]^+$	511.1228, 391.0833, 367.0815, 283.0602	DR	MSI Level 2	[1]
127	Roseoside	$C_{19}H_{30}O_8$	5.49	431.1925	431.1912	3.01	$[M+HCOO]^-$	431.1158, 385.1872, 311.0557, 152.0114, 108.0217, 59.0139	PR	MSI Level 2	[6]
128	Naringin	$C_{27}H_{32}O_{14}$	5.49	597.1826	597.1825	0.17	$[M-H+H2O]^-$	597.1835, 579.1692, 477.1406, 387.1086, 357.0983, 315.0873, 227.0926, 209.0457, 167.0351, 127.0399, 101.0243, 71.0138	AR	MSI Level 2	[2]
129	Naringenin-7-O-glucopyranoside	$C_{21}H_{22}O_{10}$	5.49	433.1149	433.1140	2.08	$[M-H]^-$	433.1160, 271.0615, 225.0769, 151.0035, 101.0243	DR	MSI Level 2	[1]
130	N-trans-p-coumaroyl octopamine	$C_{17}H_{17}NO_4$	5.50	298.1088	298.1085	1.01	$[M-H]^-$	298.1069, 280.0980, 160.0407, 145.0295, 119.0501, 100.0404	PR	MSI Level 2	[4]
131	Taxifolin	$C_{15}H_{12}O_7$	5.51	303.0514	303.0510	1.32	$[M-H]^-$	303.0139, 285.0388, 259.0253, 183.0298, 165.0194, 139.0400, 137.0244, 125.0242, 97.0294, 95.0503, 93.0346, 71.0502	PR, CF	MSI Level 2	[6]
132	5-(2-methylpropyl)-3, 6-dioxo-2-piperazinepropanoic acid	$C_{11}H_{18}N_2O_4$	5.51	241.1197	241.1194	1.24	$[M-H]^-$	241.1207, 241.0599, 197.1327, 149.0608, 141.1032, 91.0553, 82.0298	PR	MSI Level 2	[4]
133	Catechin	$C_{15}H_{14}O_6$	5.52	291.0864	291.0863	0.34	$[M+H]^+$	291.0871, 245.0809, 189.0913, 147.0442	DR, POR	MSI Level 2	[3]
134	1,2,4,6-Tetra-O-galloyl-β-D-glucose	$C_{34}H_{28}O_{22}$	5.52	787.1021	787.0999	2.80	$[M-H]^-$	787.0983, 635.0884, 617.0795, 465.0662, 313.0563, 169.0144, 125.0243	CF	MSI Level 2	[5]

No.	Chemical Name	Formula	RT/min	Experimental m/z	Calculated m/z	Error (ppm)	Precursor/adduct ion	Diagnostic MS/MS fragments	Reported botanical source	Annotation level	references
135	p-Hydroxycinnamic acid	$C_9H_8O_3$	5.53	165.0546	165.0546	0.00	$[M+H]^+$	165.0549, 147.0443, 137.0600, 119.0495, 105.0701, 91.0544, 77.0387	DR	MSI Level 2	[1]
136	Berberine	$C_{20}H_{18}NO_4^+$	5.53	336.1236	336.1236	0.00	$[M]^+$	336.1231, 321.0999, 320.0921, 306.0765, 304.0971, 292.0969, 278.0813, 234.0917, 207.6790	POR	MSI Level 2	[3]
137	8-Hydroxychromone	$C_9H_8O_3$	5.53	165.0549	165.0546	1.82	$[M+H]^+$	165.0549, 147.0443, 137.0600, 133.0286, 123.0443, 122.0365, 119.0495, 109.0650, 105.0701, 91.0544, 88.9686, 81.0700, 79.0543, 77.0387	PR	MSI Level 2	[4]
138	Isoquercetin	$C_{21}H_{20}O_{12}$	5.54	463.0891	463.0882	1.94	$[M-H]^-$	463.0882, 300.0276, 271.0251, 255.0301, 243.0298, 178.9986, 151.0036	PR, DR, CF, POR	MSI Level 2	[3]
139	Quercetin 3-O-neohesperidoside	$C_{27}H_{30}O_{16}$	5.54	609.1466	609.1461	0.82	$[M-H]^-$	609.1466, 447.0930, 301.0351, 300.0274, 271.0250, 255.0301, 243.0298, 178.9984, 151.0036	PR	MSI Level 2	[6]
140*	Rutin	$C_{27}H_{30}O_{16}$	5.54	609.1472	609.1461	1.81	$[M-H]^-$	609.1466, 541.1562, 301.0351, 300.0276, 271.0250, 255.0301, 243.0298, 178.9984, 151.0036	PR, DR	MSI Level 2	[1]
141	Hyperoside	$C_{21}H_{20}O_{12}$	5.54	465.1036	465.1028	1.72	$[M+H]^+$	465.1736, 303.0501, 285.0392, 257.0457	AR	MSI Level 2	[2]
142	Citrusin B	$C_{27}H_{36}O_{13}$	5.55	567.2095	567.2083	2.12	$[M-H]^-$	567.2278, 499.0500, 449.5014, 341.1408, 326.1159, 311.0918, 301.0350, 283.0979, 273.0775, 243.1235, 225.1136, 179.0560, 89.0244, 59.0138	CF	MSI Level 2	[5]
143	Eleutheroside e1	$C_{28}H_{36}O_{13}$	5.55	603.2064	603.2048	2.65	$[M+Na]^+$	603.2051, 503.0859, 441.1549, 440.1449, 301.0539, 150.8067, 67.0237	AR	MSI Level 2	[2]
144	Quercetin 3-O-robibioside	$C_{27}H_{30}O_{16}$	5.55	611.1616	611.1607	1.47	$[M+H]^+$	611.2318, 303.0504, 229.0495, 153.0183, 85.0284, 71.0493	AR	MSI Level 2	[2]
145	N-Acetyl-DL-tryptophan	$C_{13}H_{14}N_2O_3$	5.56	245.0935	245.0932	1.22	$[M-H]^-$	245.0929, 203.0827, 142.0664, 116.0503, 98.0247, 74.0247	AR	MSI Level 2	[2]
146	Ellagic acid	$C_{14}H_6O_8$	5.58	300.9993	300.9990	1.00	$[M-H]^-$	300.9988, 257.0097, 229.0142, 178.9987, 151.0036, 121.0294	CF	MSI Level 2	[5]
147	Rosmarinate	$C_{18}H_{16}O_8$	5.59	359.0778	359.0772	1.67	$[M-H]^-$	359.0767, 197.0457, 179.0352, 161.0245, 135.0452	DR	MSI Level 2	[1]
148	3-Methoxycatechol	$C_7H_8O_3$	5.59	141.0548	141.0546	1.42	$[M+H]^+$	141.0548, 109.0286, 95.0494, 81.0337, 53.0390	DR	MSI Level 2	[1]
149	5,7,3′-Trihydroxy-6,4′,5′-trimethoxyflavone	$C_{18}H_{16}O_8$	5.59	359.0778	359.0772	1.67	$[M-H]^-$	359.0767, 197.0457, 179.0352, 161.0245, 135.0452	DR	MSI Level 2	[1]
150	Secoxyloganin	$C_{17}H_{24}O_{11}$	5.60	403.1251	403.1246	1.24	$[M-H]^-$	403.1273, 371.0984, 223.0612, 165.0557, 121.0295, 101.0243, 95.0503, 89.0243	CF	MSI Level 2	[5]

No.	Chemical Name	Formula	RT/min	Experimental m/z	Calculated m/z	Error (ppm)	Precursor/adduct ion	Diagnostic MS/MS fragments	Reported botanical source	Annotation level	references
151	Swertiapunimarin	$C_{22}H_{32}O_{14}$	5.60	543.1707	543.1684	4.23	$[M+Na]^+$	543.2048, 270.0845, 269.0811, 211.0970, 153.0185, 141.0550, 70.0653	CF	MSI Level 2	[5]
152	Luteolin-7-rutinoside	$C_{27}H_{30}O_{15}$	5.60	593.1517	593.1512	0.84	$[M-H]^-$	593.1505, 505.1720, 417.1017, 284.0329, 255.0302, 152.0113, 108.0216	AR	MSI Level 2	[2]
153	Kaempferol 3,4'-diglucoside	$C_{27}H_{30}O_{16}$	5.60	609.1453	609.1461	-1.31	$[M-H]^-$	609.1461, 541.1562, 300.0276, 271.0251, 255.0300, 169.0143, 125.0243	PR	MSI Level 2	[6]
154*	Kaempferol	$C_{15}H_{10}O_6$	5.62	287.0554	287.0550	1.39	$[M+H]^+$	287.0552, 258.0528, 213.0548, 153.0549	AR, PR, CF	MSI Level 2	[2]
155	6-Demethoxycapillarisin	$C_{15}H_{10}O_6$	5.62	287.0554	287.0550	1.39	$[M+H]^+$	287.0552, 258.0528, 213.0548, 177.0549, 165.0185, 153.0549, 93.0335, 71.7603, 65.0388	PR	MSI Level 2	[6]
156	Narcissin	$C_{28}H_{32}O_{16}$	5.63	623.1629	623.1618	1.77	$[M-H]^-$	623.1611, 417.1044, 315.0513, 299.0193, 152.0113, 108.0217	AR	MSI Level 2	[2]
157	N-p-trans-Coumaroyltyramine	$C_{17}H_{17}NO_3$	5.63	284.1283	284.1281	0.70	$[M+H]^+$	284.1254, 147.0442, 121.0650, 119.0494, 91.0544	PR	MSI Level 2	[4]
158	Astragalin	$C_{21}H_{20}O_{11}$	5.64	449.1085	449.1078	1.56	$[M+H]^+$	449.1788, 317.0296, 287.0550, 285.0034, 257.0088, 153.0184	AR, CF, POR	MSI Level 2	[3]
159*	Cornuside	$C_{24}H_{30}O_{14}$	5.64	541.1558	541.1563	-0.92	$[M-H]^-$	541.1562, 169.0143, 125.0243	CF	MSI Level 2	[5]
160	N-trans-Feruloyltyramine	$C_{18}H_{19}NO_4$	5.64	314.1390	314.1387	0.95	$[M+H]^+$	314.1392, 254.1756, 177.0549, 145.0287, 121.0650, 117.0337	PR	MSI Level 2	[4]
161*	Neoliquiritin	$C_{21}H_{22}O_9$	5.67	463.1235	463.1235	0.00	$[M+HCOO]^-$	300.0275, 255.0301, 135.0452, 125.0243, 109.0294, 71.0138	PR	MSI Level 2	[6]
162	Querciturone	$C_{21}H_{18}O_{13}$	5.67	477.0681	477.0675	1.26	$[M-H]^-$	477.0651, 361.2254, 301.0353, 255.0299, 178.9987, 151.0036	CF,POR	MSI Level 2	[5]
163	Anisaldehyde	$C_8H_8O_2$	5.69	135.0453	135.0452	0.74	$[M-H]^-$	135.0449, 107.0501, 92.0506	DR	MSI Level 2	[1]
164*	Myricetin	$C_{15}H_{10}O_8$	5.70	317.0308	317.0303	1.58	$[M-H]^-$	317.0301, 316.9935, 289.0716, 243.1236, 225.1133, 207.1028, 181.1234, 151.0035, 137.0243, 125.0243, 99.0087	PR	MSI Level 2	[6]
165	3,4-Dihydroxybenzaldehyde	$C_7H_6O_3$	5.73	137.0245	137.0244	0.73	$[M-H]^-$	109.0295, 93.0345	DR	MSI Level 2	[1]
166	Sinapic acid	$C_{11}H_{12}O_5$	5.74	223.0617	223.0612	2.24	$[M-H]^-$	223.0614, 193.0845, 163.0402, 161.0608, 133.0659, 121.0295, 91.0553	DR	MSI Level 2	[1]

No.	Chemical Name	Formula	RT/min	Experimental m/z	Calculated m/z	Error (ppm)	Precursor/adduct ion	Diagnostic MS/MS fragments	Reported botanical source	Annotation level	references
167	Ferulic acid	$C_{10}H_{10}O_4$	5.75	193.0508	193.0506	1.04	$[M-H]^-$	193.0508, 178.0272, 149.0607, 134.0373, 79.9574	AR, DR	MSI Level 2	[1]
168	Formononetin 7-O-glucoside-6''-O-malonate	$C_{25}H_{24}O_{12}$	5.76	517.1358	517.1341	3.29	$[M+H]^+$	517.2280, 355.1731, 269.0810, 254.0574, 237.0550, 213.0909, 118.0405	AR	MSI Level 2	[2]
169*	Ononin	$C_{22}H_{22}O_9$	5.77	431.1348	431.1337	2.55	$[M+H]^+$	431.1279, 269.0810, 254.0576, 237.0547, 213.0912, 197.0603	AR	MSI Level 2	[2]
170	Astraisoflavan-7-O-β-D-glucoside	$C_{23}H_{26}O_{10}$	5.77	507.1516	507.1497	3.75	$[M+HCOO]^-$	507.1458, 299.0922, 284.0689, 269.0453, 241.0499	AR	MSI Level 2	[2]
171	Genistin	$C_{21}H_{20}O_{10}$	5.78	431.0993	431.0984	2.09	$[M-H]^-$	431.0992, 285.0406, 284.0328, 269.0458, 268.0382, 255.0302, 240.0427, 227.0351	AR	MSI Level 2	[2]
172	Pinocembrin	$C_{15}H_{12}O_4$	5.83	257.0815	257.0808	2.72	$[M+H]^+$	257.0810, 179.1057, 147.0443, 137.0235, 131.0857, 123.0440, 119.0490, 107.0493, 95.0859	PR	MSI Level 2	[6]
173	(3R)-Isomucronulatol 7-O-glucoside	$C_{23}H_{28}O_{10}$	5.85	463.1619	463.1610	1.94	$[M-H]^-$	463.2209, 301.1081, 286.0847, 271.0614, 135.0451, 121.0294, 109.0295	AR	MSI Level 2	[2]
174	Methylnissolin	$C_{17}H_{16}O_5$	5.88	301.1074	301.1071	1.00	$[M+H]^+$	301.1075, 269.0809, 241.0856, 191.0704, 167.0704, 152.0470, 147.0443, 134.0364, 123.0442	AR, PR	MSI Level 2	[2]
175	Farrerol	$C_{17}H_{16}O_5$	5.88	301.1074	301.1071	1.00	$[M+H]^+$	301.1075, 283.0997, 241.0856, 191.0704, 179.0706, 167.0704, 152.0470, 147.0443, 134.0364, 123.0442, 119.0492, 107.0492, 91.0543	PR	MSI Level 2	[6]
176	Kumatakenin	$C_{17}H_{14}O_6$	5.88	315.0866	315.0863	0.95	$[M+H]^+$	315.0866, 300.0632, 283.0596, 255.0650, 243.0656, 209.0811, 183.0445, 167.0705, 107.0493	AR	MSI Level 2	[2]
177	Pectolinarigenin	$C_{17}H_{14}O_6$	5.90	313.0722	313.0718	1.28	$[M-H]^-$	313.0711, 298.0478, 283.0248, 255.0301, 207.0663, 191.0347, 147.0451	DR	MSI Level 2	[1]
178	Azelaic acid	$C_9H_{16}O_4$	5.91	187.0977	187.0976	0.53	$[M-H]^-$	187.0974, 169.0871, 143.1074, 125.0970, 97.0658, 57.0345	AR, DR, POR	MSI Level 2	[3]
179	Disporopsin	$C_{16}H_{14}O_6$	5.92	301.0722	301.0718	1.33	$[M-H]^-$	301.0344, 271.0613, 191.0349, 179.0350, 151.0035, 135.0451, 125.0243, 121.0294, 57.0345	PR, POR	MSI Level 2	[4]
180	Salicylic acid	$C_7H_6O_3$	5.94	137.0246	137.0244	1.46	$[M-H]^-$	137.0244, 109.0290, 108.0219, 94.0377, 93.0345, 65.0397	PR	MSI Level 2	[6]
181*	Calycosin	$C_{16}H_{12}O_5$	5.94	285.0761	285.0763	-0.70	$[M+H]^+$	285.0759, 271.1765, 253.0499, 225.0548, 137.0236	AR	MSI Level 2	[2]
182	Naringenin	$C_{15}H_{12}O_5$	5.94	273.0763	273.0763	0.00	$[M+H]^+$	273.0766, 153.0184, 147.0442, 119.0494, 91.0545	DR, POR	MSI Level 2	[3]
183	Medicarpin	$C_{16}H_{14}O_4$	5.95	271.0966	271.0965	0.37	$[M+H]^+$	271.1783, 211.0753, 161.0600, 137.0600, 123.0444, 121.0681	AR	MSI Level 2	[2]
184	Alpinetin	$C_{16}H_{14}O_4$	5.96	271.0966	271.0965	0.37	$[M+H]^+$	271.0963, 253.1965, 229.0864, 211.0753, 161.0600, 137.0600, 121.0286	PR	MSI Level 2	[6]

No.	Chemical Name	Formula	RT/min	Experimental m/z	Calculated m/z	Error (ppm)	Precursor/adduct ion	Diagnostic MS/MS fragments	Reported botanical source	Annotation level	references
185	6″-O-Acetylononin	$C_{24}H_{24}O_{10}$	5.96	473.1444	473.1442	0.42	$[M+H]^+$	473.2788, 350.1953, 269.0809, 254.0578, 213.0911, 118.0417, 67.2715	AR	MSI Level 2	[2]
186	Acacetin	$C_{16}H_{12}O_5$	5.97	283.0617	283.0612	1.77	$[M-H]^-$	283.0612, 268.0379, 239.0352, 211.0403, 184.0529, 148.0164	DR	MSI Level 2	[1]
187	Sebacic acid	$C_{10}H_{18}O_4$	6.04	201.1136	201.1132	1.99	$[M-H]^-$	201.1133, 183.1026, 139.1128, 111.0816, 57.0346	DR, CF	MSI Level 2	[1]
188	2,3-Dihydroxy-9,10-DiMOPTC	$C_{17}H_{16}O_6$	6.07	315.0881	315.0874	2.22	$[M-H]^-$	315.0876, 297.0417, 285.0412, 209.0457, 193.0505, 181.0506, 175.0398, 165.0196, 149.0608, 139.0401, 121.0294, 109.0294, 65.0395	AR	MSI Level 2	[2]
189	5, 7-dihydroxy-3-(2′-hydroxy-4′-methoxybenzyl)-chroman-4-one	$C_{17}H_{16}O_6$	6.07	315.0881	315.0874	2.22	$[M-H]^-$	315.0876, 209.0457, 205.0508, 193.0505, 181.0506, 139.0401, 121.0294, 109.0294, 71.0501	PR	MSI Level 2	[4]
190	2-hydroxyoctanoic acid	$C_8H_{16}O_3$	6.09	159.1028	159.1027	0.63	$[M-H]^-$	159.1026, 130.9836, 114.9887, 113.0972, 86.9938, 59.0138	CF	MSI Level 2	[5]
191	Hydroxygenkwanin	$C_{16}H_{12}O_6$	6.09	299.0567	299.0561	2.01	$[M-H]^-$	299.0562, 284.0329, 241.0508, 227.0349, 109.0293	DR	MSI Level 2	[1]
192	Dinatin	$C_{16}H_{12}O_6$	6.09	299.0567	299.0561	2.01	$[M-H]^-$	299.0562, 284.0329, 269.0457, 256.0378, 227.0349, 148.0169, 109.0293	PR	MSI Level 2	[6]
193*	Liquiritigenin	$C_{15}H_{12}O_4$	6.10	255.0668	255.0663	1.96	$[M-H]^-$	255.0669, 153.0192, 135.0087, 119.0502, 91.0189	PR, DR	MSI Level 2	[1]
194	Apigenin-5-glucoside	$C_{21}H_{20}O_{10}$	6.14	431.0990	431.0984	1.39	$[M-H]^-$	431.0977, 269.0457, 268.0369, 225.0555, 159.1024, 96.9600	DR	MSI Level 2	[1]
195*	7,2'-Dihydroxy-3',4'-dimethoxy isoflavan	$C_{17}H_{18}O_5$	6.17	303.1233	303.1232	0.33	$[M+H]^+$	271.1782, 193.0862, 167.0705, 161.0599, 133.0650, 123.0442	AR	MSI Level 2	[2]
196*	Apigenin	$C_{15}H_{10}O_5$	6.23	269.0461	269.0455	2.23	$[M-H]^-$	269.0452, 254.0587, 241.0508, 240.0429, 239.0349, 225.1496, 211.0403, 151.0034, 135.0089, 117.0344	AR, PR	MSI Level 2	[2]
197*	Diosgenin	$C_{27}H_{42}O_3$	6.24	415.3211	415.3207	0.96	$[M+H]^+$	415.2327, 317.2091, 271.2056, 189.1124, 125.0963, 97.1014, 55.0546	PR, DR, POR	MSI Level 2	[3]
198	Picfeltarraenin IB	$C_{42}H_{64}O_{14}$	6.26	793.4365	793.4374	-1.13	$[M+H]^+$	461.2866, 207.6829, 88.1609	DR	MSI Level 2	[1]
199	Dalbergin	$C_{16}H_{12}O_4$	6.26	269.0812	269.0808	1.49	$[M+H]^+$	269.0810, 254.0577, 253.0499, 237.0547, 226.0626, 213.0913, 209.0590, 197.0599, 181.0656, 136.0156, 118.0415	PR	MSI Level 2	[6]
200*	Formononetin	$C_{16}H_{12}O_4$	6.27	267.0667	267.0663	1.50	$[M-H]^-$	267.0664, 252.0430, 223.0400, 132.0216, 91.0194	AR	MSI Level 2	[2]

No.	Chemical Name	Formula	RT/min	Experimental m/z	Calculated m/z	Error (ppm)	Precursor/adduct ion	Diagnostic MS/MS fragments	Reported botanical source	Annotation level	references
201	3,7-Dimethylgalangin	$C_{17}H_{14}O_5$	6.29	299.0916	299.0914	0.67	$[M+H]^+$	299.0919, 284.0681, 256.0730, 187.0754	AR	MSI Level 2	[2]
202	Chonglou Saponin VI	$C_{39}H_{62}O_{13}$	6.31	739.4278	739.4263	2.03	$[M+H]^+$	739.4359, 577.3749, 433.2594, 271.2059, 253.1955, 157.1015, 85.0286, 69.0701	PR	MSI Level 2	[4]
203*	Batatasin Ⅳ	$C_{15}H_{16}O_3$	6.34	289.1062	289.1071	-3.11	$[M+HCOO]^-$	243.1002, 221.1184, 177.1282, 151.1129, 149.0973, 123.0815, 77.5414	DR	MSI Level 2	[1]
204	Neocnidilide	$C_{12}H_{18}O_2$	6.38	195.1381	195.1385	-2.05	$[M+H]^+$	149.1327, 109.1012, 95.0856, 81.0701, 75.0261, 57.0702	DR	MSI Level 2	[1]
205	Arjunglucoside II	$C_{36}H_{58}O_{10}$	6.41	695.4031	695.4001	4.31	$[M+HCOO]^-$	695.4048, 649.3961, 487.3432	CF	MSI Level 2	[5]
206	Odoratumone B	$C_{17}H_{16}O_6$	6.42	315.0883	315.0874	2.86	$[M-H]^-$	315.0864, 149.0239, 139.0399, 125.0243	PR	MSI Level 2	[6]
207	5,7-dihydroxy-3-(4′-methoxybenzyl)-chroman-4-one	$C_{17}H_{16}O_5$	6.43	299.0927	299.0925	0.67	$[M-H]^-$	299.0924, 193.0505, 149.0607, 107.0501, 79.0190	PR	MSI Level 2	[4]
208	9,12,13-trihydroxy-10-octadecenoic acid	$C_{18}H_{34}O_5$	6.45	329.2335	329.2333	0.61	$[M-H]^-$	329.2334, 293.2123, 229.1442, 211.1341, 183.1385, 171.1027, 139.1128, 99.0815	PR	MSI Level 2	[4]
209	4',5,7-Trihydroxy-6-methyl-8-methoxylhomoisoflavanones	$C_{18}H_{18}O_6$	6.46	329.1036	329.1031	1.52	$[M-H]^-$	329.2335, 311.2231, 293.2116, 299.1445, 211.1342, 171.1027, 139.1128, 127.1128, 99.0816, 82.3068	PR	MSI Level 2	[6]
210	5,7-dihydroxy-6-methyl-3-(4′-methoxybenzyl)-chroman-4-one	$C_{18}H_{18}O_5$	6.46	313.1087	313.1081	1.92	$[M-H]^-$	313.1082, 207.0663, 192.0428, 179.0714, 163.0765, 135.0451	PR	MSI Level 2	[4]
211	Nandrolone	$C_{18}H_{26}O_2$	6.47	275.2009	275.2006	1.09	$[M+H]^+$	275.2015, 257.1904, 243.1499, 133.1014, 105.0701, 91.0545	AR	MSI Level 2	[2]
212	Astragaloside VI	$C_{47}H_{78}O_{19}$	6.49	969.5042	969.5030	1.24	$[M+Na]^+$	969.5038, 789.4445, 551.1650, 335.0964, 207.6691, 107.7167	AR	MSI Level 2	[2]
213*	Isorhamnetin	$C_{16}H_{12}O_7$	6.53	315.0516	315.0510	1.90	$[M-H]^-$	315.0506, 300.0274, 272.0329, 209.0719	AR, PR, DR	MSI Level 2	[1]
214	Diosgenin glucoside	$C_{33}H_{52}O_8$	6.54	577.3743	577.3735	1.39	$[M+H]^+$	577.3594, 433.2595, 415.3214, 271.2059, 253.1955, 157.1014, 145.1015, 121.1012, 85.0285	PR	MSI Level 2	[4]
215*	Astragaloside Ⅲ	$C_{41}H_{68}O_{14}$	6.58	807.4508	807.4501	0.87	$[M+Na]^+$	807.4510, 259.2297, 207.6624, 203.0527, 201.8524, 197.4682, 152.6141, 125.5979, 89.7120, 64.0713	AR	MSI Level 2	[2]
216*	Batatasin I	$C_{17}H_{16}O_4$	6.61	285.1122	285.1121	0.35	$[M+H]^+$	285.1124, 270.0888, 254.0942, 227.0705, 225.0912, 211.0759, 199.0751, 175.0393, 165.0700, 151.0392, 123.0443	DR	MSI Level 2	[1]
217	Gingerol	$C_{17}H_{26}O_4$	6.74	293.1765	293.1758	2.39	$[M-H]^-$	293.1755, 249.1864, 236.1054, 221.1548, 205.1235, 193.1598, 177.0927, 136.0893	PR	MSI Level 2	[4]

No.	Chemical Name	Formula	RT/min	Experimental m/z	Calculated m/z	Error (ppm)	Precursor/adduct ion	Diagnostic MS/MS fragments	Reported botanical source	Annotation level	references
218	Phytosphingosine	$C_{18}H_{39}NO_3$	6.86	318.3005	318.3003	0.63	$[M+H]^+$	318.3007, 300.2911, 256.2637, 102.0914, 88.0759, 70.0652	PR	MSI Level 2	[4]
219	12-Oxo-phytodienoic acid	$C_{18}H_{28}O_3$	6.93	293.2111	293.2111	0.00	$[M+H]^+$	293.2157, 275.2017, 219.1744, 131.0497, 81.0698, 67.0543, 55.0546	CF	MSI Level 2	[5]
220	Soyasaponin Bb	$C_{48}H_{78}O_{18}$	7.01	941.5115	941.5115	0.00	$[M-H]^-$	941.5127, 615.3962, 143.0349, 113.0244, 85.0295, 71.0138	AR	MSI Level 2	[2]
221	Astragaloside VII	$C_{47}H_{78}O_{19}$	7.02	969.5045	969.5030	1.55	$[M+Na]^+$	969.5046, 789.4380, 657.4015, 465.4420, 335.0952, 107.7170	AR	MSI Level 2	[2]
222	Danthron	$C_{14}H_8O_4$	7.03	239.0353	239.0350	1.26	$[M-H]^-$	223.0282, 211.0402, 193.0717, 161.0459, 102.9566	DR	MSI Level 2	[1]
223	Soyasaponin II	$C_{47}H_{76}O_{17}$	7.03	911.5053	911.5010	4.72	$[M-H]^-$	911.5026, 718.6078, 301.9798, 207.6695, 143.0353, 113.0242, 99.0087, 85.0295, 71.0137	AR	MSI Level 2	[2]
224*	Astragaloside Ⅱ	$C_{43}H_{70}O_{15}$	7.13	849.4624	849.4607	2.00	$[M+Na]^+$	849.4615, 669.3974, 477.3329, 302.0021, 203.0528, 94.3794	AR	MSI Level 2	[2]
225*	Astragaloside IV	$C_{41}H_{68}O_{14}$	7.14	807.4508	807.4501	0.87	$[M+Na]^+$	807.4507, 627.3893, 335.0946, 301.9680, 201.8519, 89.7118	AR	MSI Level 2	[2]
226	Gentrogenin	$C_{27}H_{40}O_4$	7.16	429.3006	429.3000	1.40	$[M+H]^+$	429.3056, 429.1371, 411.2911, 393.2807, 297.2226, 279.2107, 145.1014, 69.0699	PR	MSI Level 2	[4]
227	Cyrtonemoside A	$C_{51}H_{80}O_{24}$	7.17	1075.4982	1075.4967	1.39	$[M-H]^-$	1075.4969, 913.4456, 685.3594, 589.3414, 302.0045, 113.0245, 89.0243	PR	MSI Level 2	[4]
228	9, 10-dihydroxy-12(Z)-octadecenoic acid	$C_{18}H_{34}O_4$	7.21	313.2388	313.2384	1.28	$[M-H]^-$	313.2384, 295.2277, 195.1387, 183.1389, 129.0920, 99.0816	PR	MSI Level 2	[4]
229*	Methylophiopogonanone B	$C_{19}H_{20}O_5$	7.28	327.1243	327.1238	1.53	$[M-H]^-$	327.1237, 265.1811, 239.2017, 206.0586, 205.0505, 178.0636, 146.9385, 102.9487	PR, POR	MSI Level 2	[3]
230	Spiculisporid acid	$C_{17}H_{28}O_6$	7.31	327.1817	327.1813	1.22	$[M-H]^-$	327.1236, 283.1909, 265.1809, 239.2014, 206.0586, 190.9282, 178.0636, 146.9385, 102.9487	POR	MSI Level 2	[3]
231	3,5-Di-tert-butyl-4-hydroxybenzaldehyde	$C_{15}H_{22}O_2$	7.35	235.1696	235.1693	1.28	$[M+H]^+$	235.1706, 179.1069, 123.0443, 98.0968, 57.0703	CF	MSI Level 2	[5]
232*	Astragaloside Ⅰ	$C_{45}H_{72}O_{16}$	7.36	891.4728	891.4713	1.68	$[M+Na]^+$	831.4576, 711.4092, 651.3882, 544.4019, 477.3315, 301.9845, 145.1019, 90.0471	AR	MSI Level 2	[2]
233*	Dioscin	$C_{45}H_{72}O_{16}$	7.36	891.4728	891.4713	1.68	$[M+Na]^+$	891.4716, 831.4576, 711.4092, 651.3882, 477.3315, 301.9845, 203.0531, 99.0471	PR, DR, POR	MSI Level 2	[3]
234*	Higenamine	$C_{16}H_{17}NO_3$	7.38	289.1544	289.1547	-1.04	$[M+NH4]^+$	271.1789, 233.0922, 220.9348, 148.0762, 127.0390, 90.9767	DR	MSI Level 2	[1]

No.	Chemical Name	Formula	RT/min	Experimental m/z	Calculated m/z	Error (ppm)	Precursor/adduct ion	Diagnostic MS/MS fragments	Reported botanical source	Annotation level	references
235*	Ruscogenin	$C_{27}H_{42}O_4$	7.38	431.3165	431.3156	2.09	$[M+H]^+$	431.3188, 413.3091, 269.1903, 251.1804, 227.1156, 158.9640, 157.1013, 115.0763, 90.9768, 69.0701	POR	MSI Level 2	[3]
236	4-Hydroxycoumarin	$C_9H_6O_3$	7.48	163.0392	163.0390	1.23	$[M+H]^+$	163.0392, 135.0443, 133.0287, 95.0493, 92.0258, 91.0545	AR	MSI Level 2	[2]
237*	Isoastragaloside I	$C_{45}H_{72}O_{16}$	7.53	891.4727	891.4713	1.57	$[M+Na]^+$	891.4723, 764.5696, 711.4114, 581.4014, 477.3341, 402.8921, 207.6687, 99.0473	AR	MSI Level 2	[2]
238	Methyl palmitate	$C_{17}H_{34}O_2$	7.58	315.2537	315.2530	2.22	$[M+HCOO]^-$	315.2537, 301.9925, 297.2448, 178.9638, 110.9756	POR	MSI Level 2	[3]
239	Dibutyl phthalate	$C_{16}H_{22}O_4$	7.59	279.1592	279.1591	0.36	$[M+H]^+$	279.2051, 261.1602, 205.0861, 201.0472, 166.1230, 149.0236, 121.0287, 80.9479, 57.0702	PR	MSI Level 2	[6]
240	Linolenic acid	$C_{18}H_{30}O_2$	7.79	279.2320	279.2319	1.45	$[M+H]^+$	279.2325, 261.1607, 209.1539, 149.0236, 137.1328, 123.1170, 109.1014, 95.0857, 81.0700, 67.0544	CF	MSI Level 2	[5]
241	Emodin	$C_{15}H_{10}O_5$	7.82	269.0460	269.0455	1.86	$[M-H]^-$	269.0457, 241.0506, 225.0557, 213.0558, 197.0609, 67.2604	AR	MSI Level 2	[2]
242	Acetylastragaloside I	$C_{47}H_{74}O_{17}$	7.88	933.4837	933.4818	2.04	$[M+Na]^+$	933.4821, 873.4496, 753.4193, 693.3937, 459.1089, 301.9937, 207.6768	AR	MSI Level 2	[7]
243	Maslinic acid	$C_{30}H_{48}O_4$	8.37	471.3483	471.3480	1.45	$[M-H]^-$	471.3480, 397.5546, 301.9891, 185.1739	CF	MSI Level 2	[5]
244	4-Dodecylbenzenesulfonic acid	$C_{18}H_{30}O_3S$	8.49	325.1847	325.1843	1.23	$[M-H]^-$	325.1836, 301.9936, 253.2661, 197.0271, 184.0187, 183.0119, 144.9428, 121.9438, 101.8262	DR	MSI Level 2	[1]
245	Corosolic acid	$C_{30}H_{48}O_4$	8.54	471.3485	471.3480	1.45	$[M-H]^-$	471.3480, 177.8443, 52.3721	CF	MSI Level 2	[5]
246*	Ursolic acid	$C_{30}H_{48}O_3$	9.45	439.3571	439.3576	-1.14	$[M+H-H2O]^+$	439.3594, 393.3523, 295.0271, 249.1848, 203.1798, 191.1796, 147.1168, 119.0856, 95.0857	AR, CF	MSI Level 2	[2]
247*	Oleanolic acid	$C_{30}H_{48}O_3$	9.52	501.3589	501.3575	2.79	$[M+HCOO]^-$	455.3551, 301.9714, 209.4798, 160.9747, 114.2099, 102.9565, 92.9279, 66.5211, 50.6706	CF	MSI Level 2	[5]
248	12-Ketodeoxycholic acid	$C_{24}H_{38}O_4$	10.33	391.2842	391.2848	-1.53	$[M+H]^+$	253.8356, 149.0236, 71.0856	DR	MSI Level 2	[1]
249	Erucamide	$C_{22}H_{43}NO$	11.22	338.3416	338.3417	0.35	$[M+H]^+$	338.1384, 322.1070, 307.1199, 306.1125, 304.0971, 292.0970	CF	MSI Level 2	[5]
250*	Stigmasterol	$C_{29}H_{48}O$	11.33	395.3675	395.3672	0.76	$[M+H-H2O]^+$	395.3681, 255.2119, 207.6613, 159.1172, 145.1014, 95.0858, 81.0702, 69.0701, 57.0703	DR	MSI Level 2	[1]

Note: Asterisks (*) denote representative literature-supported marker constituents highlighted in Fig. 2. Table S1 now explicitly reports molecular formula, retention time, experimental and calculated m/z, mass error, precursor/adduct ion, diagnostic MS/MS fragments, reported botanical source, annotation level, and supporting reference(s). Among the 250 retained constituents, 249 were reported as putative MSI Level 2 annotations according to the Metabolomics Standards Initiative [8]. No. 38 (4-guanidinobutanoate) lacked diagnostic MS/MS fragment information and was therefore conservatively retained as an MSI Level 3 tentative assignment. An initial mass

tolerance of 10 ppm was used only as a candidate-search window, whereas all retained annotations showed absolute precursor-mass errors within 5 ppm. Final Level 2 annotations were evaluated using integrated evidence from accurate mass, precursor/adduct assignment, diagnostic MS/MS fragmentation patterns, retention behavior, botanical-source information, and literature-reported mass-spectrometric evidence. No fixed minimum number of matching fragment ions or numerical matching-score threshold was applied, and authentic-standard retention-time or MS/MS matching was not used for the UHPLC-Orbitrap profiling annotations. For isomeric constituents that could not be distinguished unambiguously by the available retention and MS/MS evidence, the reported compound names should be interpreted as putative structural assignments rather than definitive identifications. Botanical-source abbreviations: AR, Astragali Radix; DR, Dioscoreae Rhizoma; CF, Corni Fructus; PR, Polygonati Rhizoma; POR, Polygonati Odorati Rhizoma. Supporting reference numbers correspond to the references listed below.

Table S2. Calibration curves and contents of representative marker constituents in the SQJ botanical extract.

Marker constituent	Calibration equation	R^2	Linear range (μg/mL)	Content (mg/g)
Morroniside	A = 52407.36C − 19623.96	0.9997	1.8602–238.1	2.169 ± 0.105
Loganin	A = 37909.61C + 22248.72	0.9992	1.8602–238.1	1.569 ± 0.084
Calycosin-7-O-glucoside	A = 67956.70C − 7116.11	0.9996	0.1860–23.81	0.218 ± 0.030
Astragaloside IV	ln A = 1.5723 ln C + 5.5022	0.9990	48.125–770	0.1816 ± 0.0023

Note: For morroniside, loganin, and calycosin-7-O-glucoside, calibration curves were constructed by linear regression of peak area (A) against concentration (C) using eight concentration levels. For astragaloside IV, the HPLC-ELSD response was evaluated by linear regression of ln(peak area) against ln(concentration) using six concentration levels. Contents are expressed as mean ± SD from three SQJ batches (n = 3). Morroniside, loganin, and calycosin-7-O-glucoside were quantified by UPLC-UV, whereas astragaloside IV was quantified by HPLC-ELSD.

Table S3. Putatively annotated differential serum metabolites.

No.	HMDB	Metabolites	Formula	t_R/min	Adduct ion	m/z	Changing trends				*VIP*	Raw *P* value (CON vs MOD)	BH-adjusted *P* value
							MOD vs CON	MET vs MOD	SQJL vs MOD	SQJH vs MOD			
1	HMDB0000230	Neu5Ac	$C_{11}H_{19}NO_9$	0.801	$[M-H]^-$	308.09899	↑**	↓	↓*	↓*	1.10275	1.654575E-04	1.407403E-03
2	HMDB0033143	Pyrraline	$C_{12}H_{18}N_2O_4$	0.811	$[M+Na]^+$	277.1158	↑*	↓	↓*	↓	1.11469	4.628523E-03	3.052387E-02
3	HMDB0000158	Tyrosine	$C_9H_{11}NO_3$	1.149	$[M+H]^+$	182.08129	↑**	↓	↓	↓*	3.94937	2.934300E-04	3.151710E-03
4	HMDB0000423	DHCA	$C_9H_{10}O_4$	1.149	$[M+H-H2O]^+$	165.05476	↑**	↓	↓	↓**	2.7307	1.692289E-04	1.981135E-03
5	HMDB0010715	Phenylacetamide	C_8H_9NO	1.149	$[M+H]^+$	136.0758	↑**	↓	↓	↓**	2.68926	1.732501E-04	2.006557E-03
6	HMDB0000205	Phenylpyruvic acid	$C_9H_8O_3$	1.149	$[M+H]^+$	147.04416	↑**	↓	↓	↓**	1.39829	2.461400E-04	2.715466E-03
7	HMDB0011718	4-HBA	$C_7H_6O_2$	1.149	$[M+H]^+$	123.04425	↑**	↓	↓	↓**	1.18129	1.764661E-04	2.032952E-03
8	HMDB0000201	Acetylcarnitine	$C_9H_{17}NO_4$	4.959	$[M-H]^-$	202.10779	↑***	↓	↓***	↓***	1.32017	2.073200E-05	2.379381E-04
9	HMDB0004708	9,12,13-TriHOME	$C_{18}H_{34}O_5$	7.452	$[M-H]^-$	329.23367	↑**	↓	↓	↓*	3.41602	4.733400E-04	3.396266E-03
10	HMDB0010388	LysoPC(18:3/0:0)	$C_{26}H_{48}NO_7P$	7.921	$[M+H]^+$	518.32439	↑**	↓	↓	↓**	1.03735	1.148746E-03	9.563920E-03
11	HMDB0004685	13,14-dh-15-k-PGF2α	$C_{20}H_{34}O_5$	8.117	$[M-H]^-$	353.23377	↑***	↓	↓	↓***	2.44733	4.150215E-05	4.277913E-04
12	HMDB0010212	17,18-EpETE	$C_{20}H_{30}O_3$	8.13	$[M+H]^+$	319.22658	↑**	↓	↓	↓**	1.05489	3.687722E-04	3.828291E-03
13	HMDB0006940	9(S)-HPODE	$C_{18}H_{32}O_4$	8.555	$[M-H]^-$	311.22318	↑**	↓	↓*	↓	1.22473	7.271182E-04	4.857820E-03
14	HMDB0013623	12(13)Ep-9-KODE	$C_{17}H_{28}O_2$	9.151	$[M+FA-H]^-$	309.20746	↑***	↓	↓	↓*	1.49102	5.710835E-06	8.043841E-05
15	HMDB0011525	LysoPE(22:5/0:0)	$C_{27}H_{46}NO_7P$	9.765	$[M-H]^-$	526.2947	↓*	↑**	↑***	↑***	1.13072	2.034499E-03	1.171550E-02
16	HMDB0004705	12,13-DiHOME	$C_{18}H_{34}O_4$	10.018	$[M-H]^-$	295.22794	↑*	↓	↓**	↓***	7.38743	2.509207E-03	1.412105E-02
17	HMDB0001388	Linolenic acid	$C_{18}H_{30}O_2$	10.039	$[M+H]^+$	279.23183	↑**	↓	↓**	↓***	4.2155	8.973117E-04	7.935600E-03
18	HMDB0004668	13-OxoODE	$C_{18}H_{30}O_3$	10.364	$[M-H]^-$	293.21248	↑**	↓	↓*	↓**	1.95728	6.119218E-04	4.249171E-03
19	HMDB0010393	LysoPC(20:3/0:0)	$C_{28}H_{52}NO_7P$	10.501	$[M+H]^+$	546.35486	↓***	↑	↑**	↑	2.1791	4.873717E-06	1.063873E-04
20	HMDB0010200	alpha-12(13)-EpODE	$C_{18}H_{30}O_3$	10.635	$[M+H]^+$	295.22702	↑**	↓	↓	↓*	1.41548	9.905523E-04	8.483559E-03

21	HMDB0010401	LysoPC(22:4/0:0)	$C_{30}H_{54}NO_7P$	11.009	$[M+H]^+$	572.3711	↓***	↑	↑**	↑	2.39134	8.445095E-07	2.410927E-05
22	HMDB0011130	LysoPE(18:0/0:0)	$C_{23}H_{48}NO_7P$	11.751	$[M+H]^+$	482.32416	↑***	↓	↓	↓*	3.22798	6.998206E-09	4.179020E-07
23	HMDB0000308	3β-HCA	$C_{24}H_{38}O_3$	11.842	$[M-H]^-$	373.2751	↑**	↓	↓	↓**	1.62156	1.199463E-03	7.471028E-03
24	HMDB0011697	SM(d18:1/24:0)	$C_{47}H_{95}N_2O_6P$	15.128	$[M+H]^+$	815.69809	↑**	↑	↓***	↓	2.01868	7.984144E-05	1.073735E-03

Note: Differential metabolites between CON and MOD were defined using the combined criteria of *VIP* > 1.0 and Benjamini-Hochberg (BH)-adjusted $p < 0.05$. *VIP* values were obtained from the OPLS-DA model. Raw *p* values were calculated using two-sided unpaired Student's t-tests, and BH correction was applied across all eligible metabolic features included in the CON-versus-MOD statistical analysis before differential-metabolite selection. In the changing-trend columns, ↑ and ↓ indicate higher and lower normalized peak areas, respectively, for the comparison shown. Asterisks indicate exploratory pairwise differences evaluated using the Kruskal-Wallis test followed by uncorrected Dunn's post hoc test (*$p < 0.05$, **$p < 0.01$, ***$p < 0.001$, and ****$p < 0.0001$); these asterisks were not used for differential-metabolite selection. Metabolites were putatively annotated (MSI level 2) based on accurate mass, retention time, isotope pattern, and MS/MS spectral matching. Positional and stereochemical assignments of oxylipins and closely related lipid species should be interpreted cautiously unless confirmed using authentic reference standards.

Table S4. The linear parameters of each SCFA.

SCFAs	Linear equation	R^2	Linearity range (μg/mL)
Acetic acid	y=0.0529x+0.0091	0.9967	35.18-2251.76
Propionic acid	y=0.1475x+0.0434	0.9921	43.40-2777.86
Isobutyric acid	y=0.2124x-0.0001	0.9978	3.23-206.50
Butyric acid	y=0.2345x+0.0538	0.9936	51.62-3303.96
Isovaleric acid	y=1.052x+0.0181	0.9931	3.74-239.36
Valeric acid	y=0.9319x+0.0003	0.9986	3.74-239.36

Note: Each component is related linearly in the corresponding concentration range, with R^2 values greater than 0.99.

Table S5. Precision, repeatability and stability of SCFAs (n = 6).

SCFAs	Precision (RSD/%, n = 6)	Stability (RSD/%, n = 6)	Repeatability (RSD/%, n = 6)
Acetic acid	0.65	1.67	2.89
Propionic acid	1.26	2.41	2.54
Isobutyric acid	1.84	1.33	3.71
Butyric acid	0.97	2.05	2.36
Isovaleric acid	2.13	1.79	3.92
Valeric acid	1.11	2.28	4.18

Note: Each compound's precision relative standard deviation (RSD) was determined to be less than 3.0%. Repeatability experiments conducted on various components resulted in an RSD below 5.0%. Moreover, all components demonstrated excellent stability over 48 hours, with RSD values below 3.0%.

Table S6. Overall and pairwise PERMANOVA results for gut microbial beta diversity.

Distance metric	Group comparison	Sample size	R^2	pseudo-F	Raw *P*	BH-adjusted *P*	No. of permutations
**Bray-Curtis**	**Overall five-group comparison**	**30 (6/group)**	**0.314**	**2.863**	**<0.001**	**NA**	***9999***
Bray-Curtis	CON vs MOD	6 vs 6	0.290	4.089	0.0017	0.0024	*9999*
Bray-Curtis	CON vs MET	6 vs 6	0.252	3.375	0.0017	0.0024	*9999*
Bray-Curtis	CON vs SQJL	6 vs 6	0.332	4.959	0.0017	0.0024	*9999*
Bray-Curtis	CON vs SQJH	6 vs 6	0.313	4.559	0.0017	0.0024	*9999*
Bray-Curtis	MOD vs MET	6 vs 6	0.177	2.150	0.0017	0.0024	*9999*
Bray-Curtis	MOD vs SQJL	6 vs 6	0.241	3.179	0.0017	0.0024	*9999*
Bray-Curtis	MOD vs SQJH	6 vs 6	0.205	2.572	0.0017	0.0024	*9999*
Bray-Curtis	MET vs SQJL	6 vs 6	0.133	1.534	0.0266	0.0296	*9999*
Bray-Curtis	MET vs SQJH	6 vs 6	0.143	1.667	0.0235	0.0294	*9999*
Bray-Curtis	SQJL vs SQJH	6 vs 6	0.121	1.371	0.1646	0.1646	*9999*
**Jaccard**	**Overall five-group comparison**	**30 (6/group)**	**0.204**	**1.599**	**<0.001**	**NA**	***9999***
Jaccard	CON vs MOD	6 vs 6	0.147	1.725	0.0017	0.0024	*9999*
Jaccard	CON vs MET	6 vs 6	0.153	1.808	0.0017	0.0024	*9999*
Jaccard	CON vs SQJL	6 vs 6	0.173	2.095	0.0017	0.0024	*9999*
Jaccard	CON vs SQJH	6 vs 6	0.169	2.030	0.0017	0.0024	*9999*
Jaccard	MOD vs MET	6 vs 6	0.120	1.369	0.0017	0.0024	*9999*
Jaccard	MOD vs SQJL	6 vs 6	0.144	1.676	0.0017	0.0024	*9999*
Jaccard	MOD vs SQJH	6 vs 6	0.131	1.505	0.0017	0.0024	*9999*
Jaccard	MET vs SQJL	6 vs 6	0.117	1.325	0.0105	0.0117	*9999*
Jaccard	MET vs SQJH	6 vs 6	0.114	1.281	0.0061	0.0076	*9999*
Jaccard	SQJL vs SQJH	6 vs 6	0.109	1.220	0.0505	0.0505	*9999*
**Unweighted UniFrac**	**Overall five-group comparison**	**30 (6/group)**	**0.244**	**2.012**	**<0.001**	**NA**	***9999***
Unweighted UniFrac	CON vs MOD	6 vs 6	0.172	2.083	0.0017	0.0042	*9999*
Unweighted UniFrac	CON vs MET	6 vs 6	0.182	2.230	0.0017	0.0042	*9999*
Unweighted UniFrac	CON vs SQJL	6 vs 6	0.220	2.823	0.0017	0.0042	*9999*
Unweighted UniFrac	CON vs SQJH	6 vs 6	0.205	2.580	0.0017	0.0042	*9999*
Unweighted UniFrac	MOD vs MET	6 vs 6	0.165	1.972	0.0037	0.0069	*9999*
Unweighted UniFrac	MOD vs SQJL	6 vs 6	0.202	2.527	0.0048	0.0069	*9999*
Unweighted UniFrac	MOD vs SQJH	6 vs 6	0.170	2.050	0.0048	0.0069	*9999*
Unweighted UniFrac	MET vs SQJL	6 vs 6	0.120	1.360	0.0406	0.0507	*9999*

Unweighted UniFrac	MET vs SQJH	6 vs 6	0.112	1.261	0.0555	0.0617	*9999*
Unweighted UniFrac	SQJL vs SQJH	6 vs 6	0.105	1.174	0.1750	0.1750	*9999*
**Weighted UniFrac**	**Overall five-group comparison**	**30 (6/group)**	**0.503**	**6.317**	**<0.001**	**NA**	***9999***
Weighted UniFrac	CON vs MOD	6 vs 6	0.319	4.679	0.0077	0.0110	*9999*
Weighted UniFrac	CON vs MET	6 vs 6	0.227	2.941	0.0059	0.0098	*9999*
Weighted UniFrac	CON vs SQJL	6 vs 6	0.561	12.777	0.0017	0.0042	*9999*
Weighted UniFrac	CON vs SQJH	6 vs 6	0.495	9.809	0.0017	0.0042	*9999*
Weighted UniFrac	MOD vs MET	6 vs 6	0.319	4.685	0.0048	0.0096	*9999*
Weighted UniFrac	MOD vs SQJL	6 vs 6	0.583	13.986	0.0017	0.0042	*9999*
Weighted UniFrac	MOD vs SQJH	6 vs 6	0.502	10.096	0.0017	0.0042	*9999*
Weighted UniFrac	MET vs SQJL	6 vs 6	0.262	3.557	0.0183	0.0229	*9999*
Weighted UniFrac	MET vs SQJH	6 vs 6	0.196	2.438	0.0335	0.0372	*9999*
Weighted UniFrac	SQJL vs SQJH	6 vs 6	0.073	0.784	0.5802	0.5802	*9999*

Note: PERMANOVA was performed with *9,999* permutations. For each distance metric, Benjamini-Hochberg correction was applied across the 10 pairwise group comparisons. BH adjustment was not applicable to the overall five-group test (NA).

Table S7. Gene-level differential-expression summary, cross-dataset consistency analysis, and transcriptomic evidence for HPD and GSTM3.

Panel A. Dataset-level summary

Dataset	Control/T1DM, *n*	Genes tested	Nominal candidates, *n*	FDR DEGs, *n*
GSE55098	10/12	20,845	153	0
GSE156035	20/20	43,315	1,540	311
Cross-dataset	—	19,599 common	12 shared (11 same direction)	0 shared

Panel B. Genes meeting the nominal exploratory threshold in both datasets

Gene	log_2FC GSE55098	Raw *p* GSE55098	BH *p* GSE55098	log_2FC GSE156035	Raw *p* GSE156035	BH *p* GSE156035	Direction
ANP32A-IT1	0.590	6.66e-05	0.0913	0.594	7.67e-05	0.0195	Same
DUSP2	-1.273	0.00359	0.238	-1.064	0.00831	0.134	Same
STEAP4	0.771	0.0105	0.316	0.688	0.014	0.171	Same
DDIT4	-0.956	0.0108	0.317	-1.276	0.000577	0.0401	Same
KCNJ2	1.038	0.0121	0.326	0.793	0.0123	0.16	Same
DHRS9	0.856	0.0153	0.346	1.174	0.00417	0.0979	Same
BTNL8	0.703	0.0205	0.362	0.713	0.00254	0.0776	Same
CD24	1.080	0.0231	0.372	0.798	0.0109	0.152	Same
CEACAM8	1.833	0.0321	0.405	1.739	0.00289	0.0819	Same
NAP1L5	-0.698	0.0359	0.419	-0.803	0.00292	0.0823	Same
LTF	1.390	0.0362	0.419	2.566	0.000236	0.0265	Same
NLRP2	-0.661	0.000373	0.142	0.795	0.0326	0.246	Opposite

Panel C. Transcriptomic evidence for the experimentally evaluated candidates

Gene	Dataset	log_2FC	Raw *p*	BH-adjusted *p*	FDR DEG
GSTM3	GSE156035	0.626	0.0703	0.339	No
GSTM3	GSE55098	-0.325	0.215	0.662	No
HPD	GSE156035	-1.058	0.000248	0.027	Yes
HPD	GSE55098	-0.034	0.524	0.853	No

Note: Gene-level differences between T1DM and control samples were evaluated independently in each dataset using two-sided Welch's t-tests. Benjamini–Hochberg correction was applied across all annotated genes tested within each dataset. FDR-supported DEGs were defined by BH-adjusted $p < 0.05$ and $|log_2FC| > 0.585$. The nominal threshold (raw $p < 0.05$ and $|log_2FC| > 0.585$) was used only for exploratory cross-dataset consistency analysis. Of the 12 shared nominal candidates, 11 were directionally concordant; NLRP2 changed in opposite directions. No gene met the FDR criterion in both datasets.

Supplementary Figures

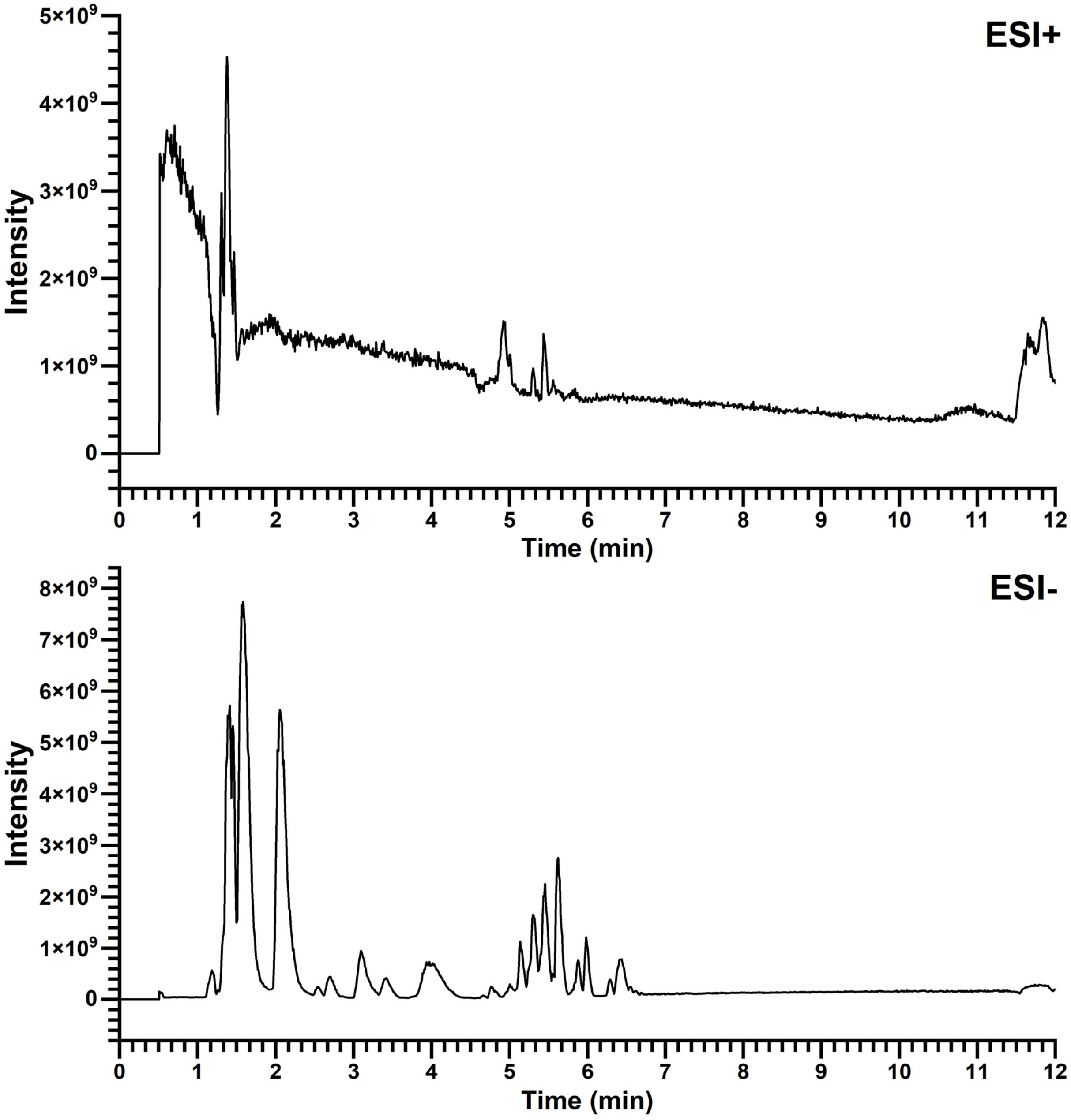

Figure S1. Total ion chromatograms of chemical constituents in SQJ botanical extract.

Upper panel: total ion chromatogram (TIC) acquired in positive electrospray ionization mode (ESI+). Lower panel: TIC acquired in negative electrospray ionization mode (ESI-).

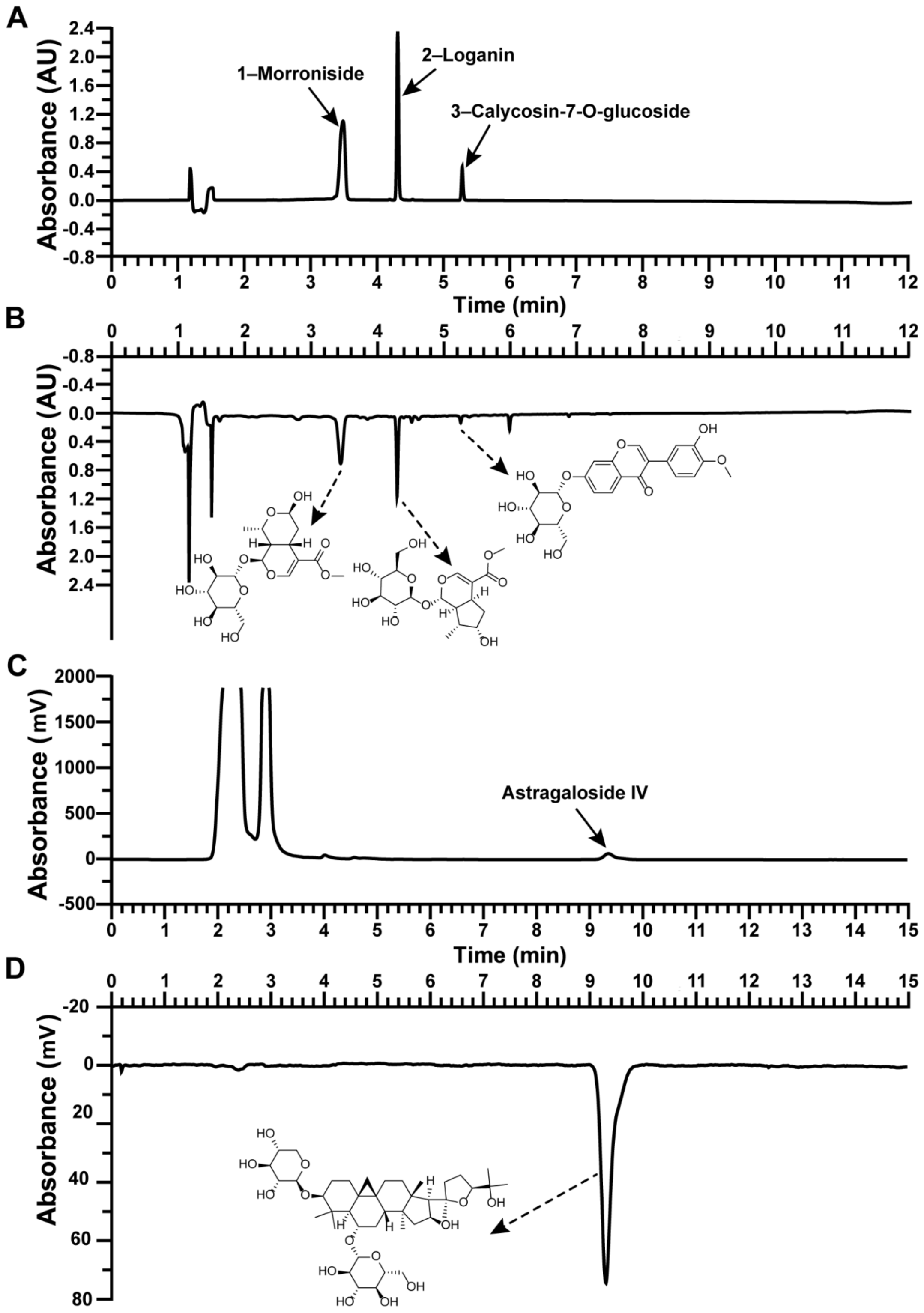

Figure S2. Representative chromatograms for quantitative determination of marker constituents in SQJ botanical extract.

(A) UPLC-UV chromatogram of mixed reference standards containing morroniside, loganin, and calycosin-7-O-glucoside. (B) UPLC-UV chromatogram of the SQJ sample, showing the corresponding peaks assigned to morroniside, loganin, and calycosin-7-O-glucoside. (C) HPLC-ELSD chromatogram of the SQJ sample, showing the corresponding peak assigned to astragaloside IV. (D) HPLC-ELSD chromatogram of the astragaloside IV reference standard.

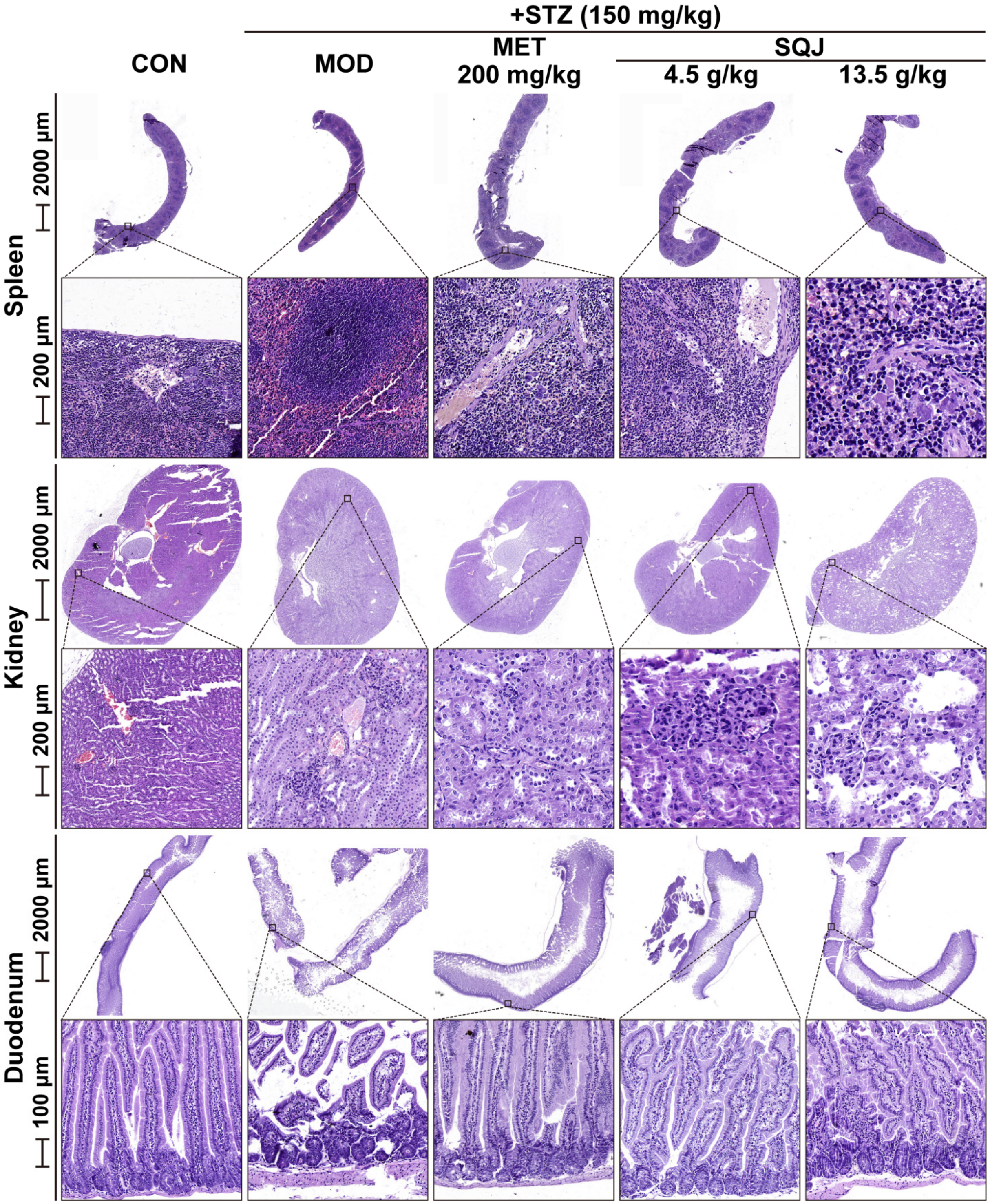

Figure S3. Histopathological evaluation of the spleen, kidney, and duodenum in STZ-induced diabetic mice after SQJ botanical extract intervention.

Representative hematoxylin and eosin (*H&E*)-stained sections of the spleen, kidney, and duodenum from the CON, MOD, MET, SQJL, and SQJH groups. For each tissue, low-magnification overview images and corresponding enlarged regions are shown. Scale bars: 2000 μm for overview images and 100 or 200 μm for magnified images, as indicated. Histopathological observations were performed with $n = 3$ per group.

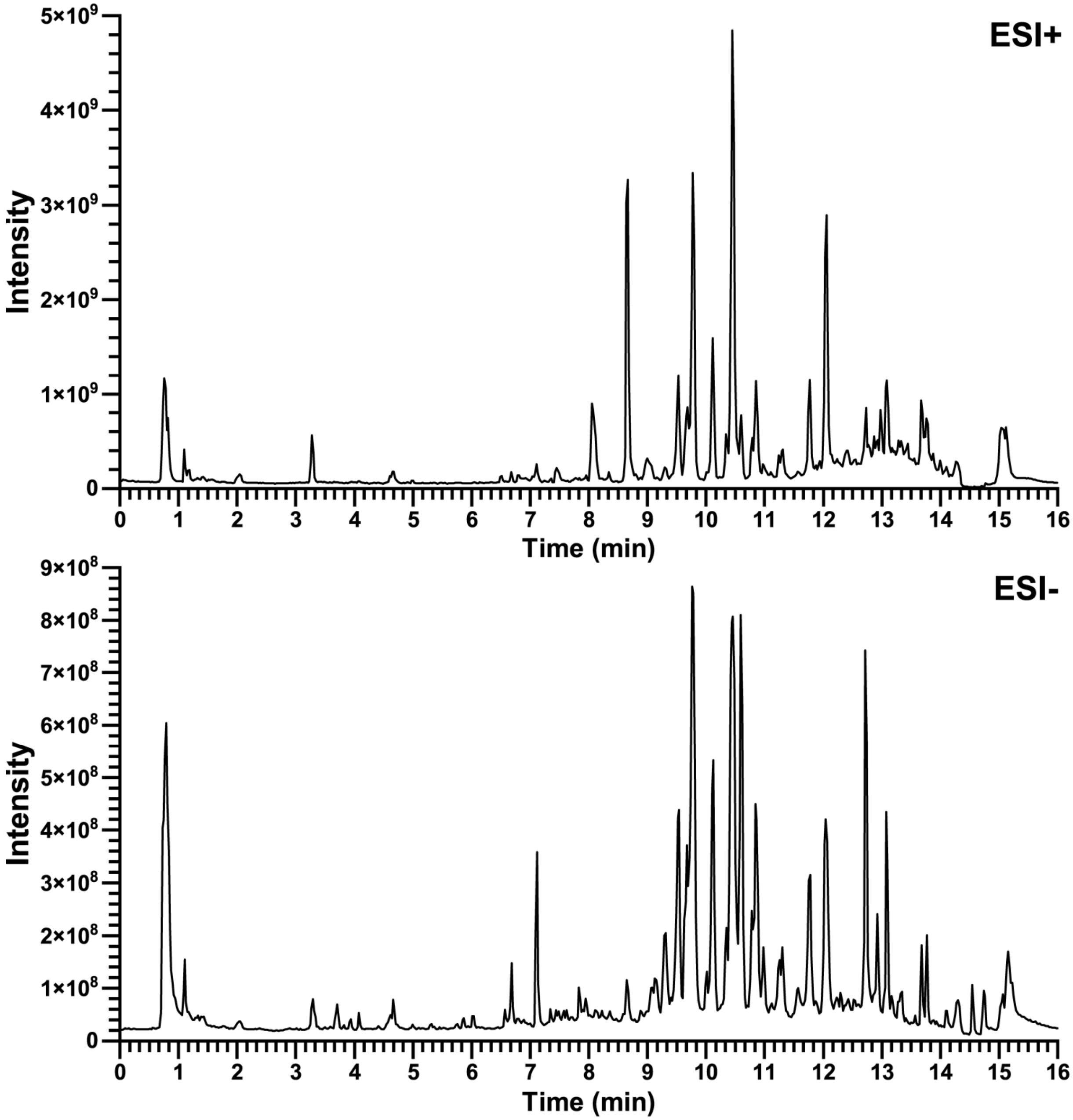

Figure S4. Total ion chromatograms of serum QC samples acquired in positive and negative ion modes.

Upper panel: TIC acquired in positive electrospray ionization mode (ESI+). Lower panel: TIC acquired in negative electrospray ionization mode (ESI-).

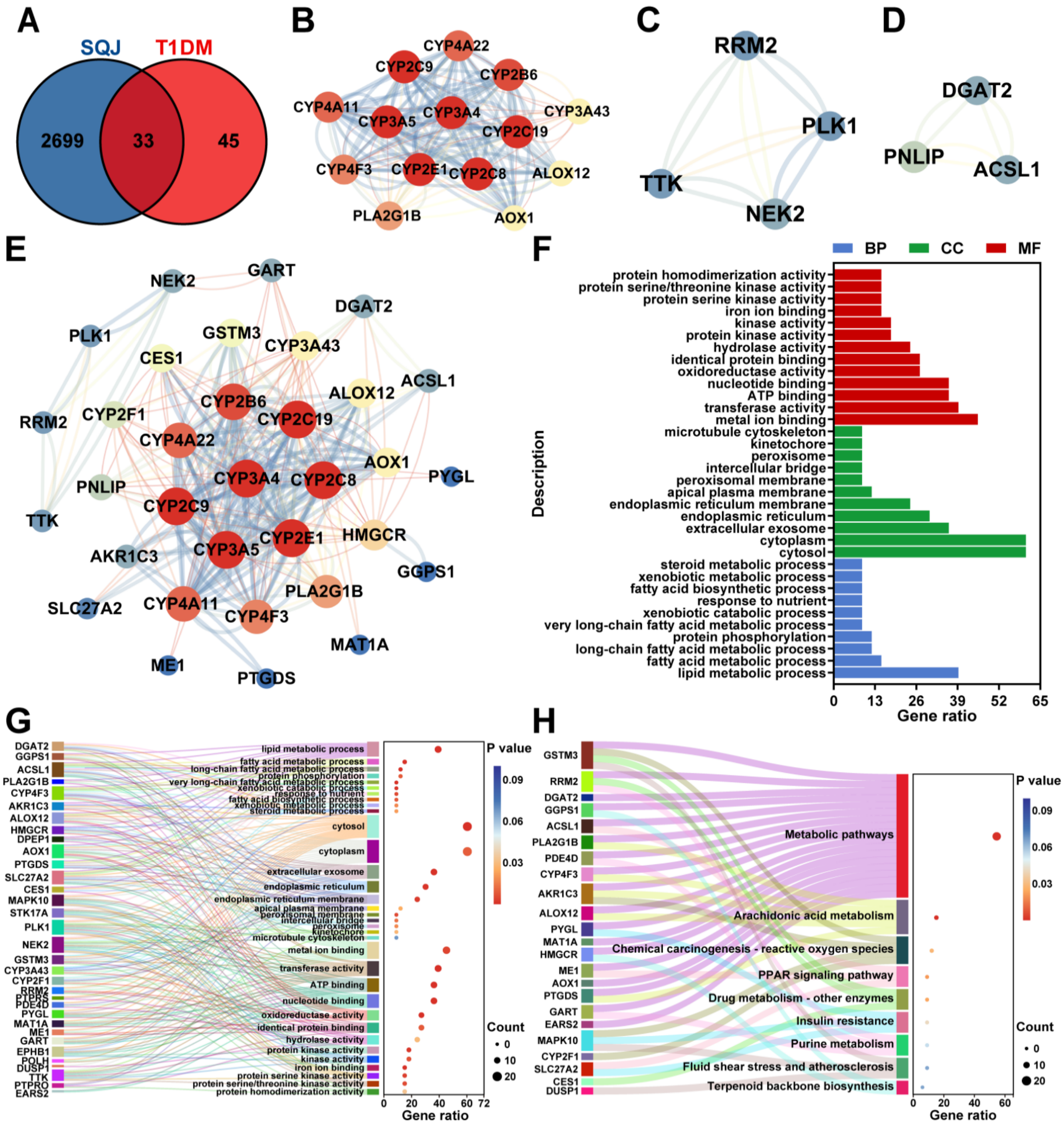

Figure S5. Exploratory network pharmacology-assisted compound–target analysis for hypothesis generation.

(A) Venn diagram showing the overlap between SQJ-associated putative targets and the exploratory diabetes-related candidate set derived from the non-redundant union of genes meeting the original nominal threshold (raw $p < 0.05$ and $|\log2FC| > 0.585$) in either GSE55098 or GSE156035. **(B–D)** Topologically significant protein-protein interaction (PPI) subnetworks identified from the overlapping targets using the MCODE plugin in Cytoscape v3.8.2. **(B)** CYP-enriched oxidoreductase/xenobiotic metabolism-related subnetwork. **(C)** Cell-cycle-related subnetwork. **(D)** Lipid metabolism-related subnetwork. Node size and color intensity indicate topological importance, and edge thickness reflects interaction confidence. **(E)** Global PPI network of the 33 overlapping targets. Node size and color intensity indicate topological importance, whereas edge thickness reflects interaction confidence. The 33-gene network is retained as an exploratory, hypothesis-generating analysis and should not be interpreted as FDR-supported or cross-dataset replicated transcriptomic evidence. **(F)** Gene Ontology (GO) enrichment analysis of the overlapping targets, including biological process (BP), cellular component (CC), and molecular function (MF) categories. Representative enriched GO terms with $p < 0.05$ are shown in the clustered bar plot. **(G)** Sankey-bubble plot linking overlapping SQJ-diabetes targets to enriched GO terms. **(H)** Sankey-bubble plot linking overlapping SQJ-diabetes targets to enriched KEGG pathways.

References

1 An, L.; Chen, H.; Zhou, J.; Ma, H.; Li, M.; Ma, J.; Li, T.; Xu, H.; Liang, H.; Wu, X. Uncovering Bioactive Constituents and Mechanisms of Dioscorea batatas Bean via Integrated UPLC-Q-TOF-MS/MS, Bioinformatics and Molecular Simulation. J. Chin. Inst. Food Sci. Technol. 2025, 25, 59–75. https://doi.org/10.16429/j.1009-7848.2025.08.006.

2 Xu, R.; Fan, J.; Dong, H.; Chen, X.; Xu, J.; Huo, J.; Ju, J. UPLC-Q-TOF-MS Analysis on Chemical Constituents of Classical Prescription Huangqi Guizhi Wuwu Tang Standard Decoction. China J. Chin. Mater. Med. 2020, 45, 5614–5630, doi:10.19540/j.cnki.cjcmm.20200915.301.

3 Sui, Z.; Hou, P.; Wang, B. Rapid Identification of the Components in Polygonatum odoratum Based on UPLC-Q-Orbitrap Platform. China Pharmacist 2022, 25, 626–630. https://doi.org/10.19962/j.cnki.issn1008-049X.2022.04.011.

4 Yan, Z.; Ge, H.; Luo, Z.; Xu, J.; Yin, X.; Peng, X.; Cai, Y.; Chen, L, Study on differences in inhibitory effects of nine-processed Polygonatum cyrtonema and raw Polygonatum cyrtonema on lipopolysaccharide-induced inflammatory responses in RAW 264.7 cells based on UPLC-Q-TOF-MS/MS network pharmacology, *Drug Eval. Res.*, 2025, **48**, 3551–3569. Available online: CNKI (accessed on 9 October 2026).

5 Qian, Y.; Pi, W.; Zhu, G.; Wei, W.; Lu, T.; Mao, C. Quality Evaluation of Raw and Processed Corni Fructus by UHPLC-QTOF-MS and HPLC Coupled with Color Determination. J. Pharm. Biomed. Anal. 2022, 218, 114842, doi:10.1016/j.jpba.2022.114842.

6 Gao, X.; He, M.; Luo, J.; Yan, Y.; Wang, Q.; Guo, J.; Luo, K.; Peng, S. Chemical Components and Potential Mechanisms of Extracts from Polygonatum sibiricum in Alleviating Hyperuricemia Based on UPLC-ESI-QTRAP-MS/MS and Network Pharmacology. Nat. Prod. Res. Dev. 2026, 38, 496–508. https://doi.org/10.16333/j.1001-6880.2026.3.005.

7 Li, C.; Lu, M.; Liao, J.; Huang, L.; Ju, W.; Zou, J. Systematic Screening and Identification of Chemical Constituents in Qikui Granules Based on UPLC-QTOF-MS/MS Analysis. J. Nanjing Univ. Tradit. Chin. Med. 2023, 39, 57–69. https://doi.org/10.14148/j.issn.1672-0482.2023.0057.

8 L. W. Sumner, A. Amberg, D. Barrett, M. H. Beale, R. Beger, C. A. Daykin, T. W.-M. Fan, O. Fiehn, R. Goodacre, J. L. Griffin, T. Hankemeier, N. Hardy, J. Harnly, R. Higashi, J. Kopka, A. N. Lane, J. C. Lindon, P. Marriott, A. W. Nicholls, M. D. Reily, J. J. Thaden, and M. R. Viant, Proposed minimum reporting standards for chemical analysis: chemical analysis working group (CAWG) metabolomics standards initiative (MSI), *Metabolomics*, 2007, **3**, 211–221.
